# Dynamic Changes in Prokaryotic and Eukaryotic Communities and Networks in Minimally Managed Cabbage-Cultivated Field Soils

**DOI:** 10.3390/genes16050482

**Published:** 2025-04-24

**Authors:** Sentaro Ito, Junya Murakami, Mio Suzuki, Yuu Hirose, Takahiro Yamauchi, Toshihiko Eki

**Affiliations:** 1 Molecular Genetics Laboratory, Department of Applied Chemistry and Life Science, Toyohashi University of Technology, 1-1 Hibarigaoka, Tempaku-cho, Toyohashi 441-8580, Japan; 2 Laboratory of Genomics and Photobiology, Department of Applied Chemistry and Life Science, Toyohashi University of Technology, 1-1 Hibarigaoka, Tempaku-cho, Toyohashi 441-8580, Japan; 3 Research Center for Agrotechnology and Biotechnology, Toyohashi University of Technology, 1-1 Hibarigaoka, Tempaku-cho, Toyohashi 441-8580, Japan

**Keywords:** DNA metabarcoding, co-occurrence network, soil biota, minimal agricultural practice

## Abstract

Background/Objectives: Taxonomic profiling of soil microbial communities is useful for assessing and monitoring the biological status of agricultural land. In this study, we aimed to investigate changes in the taxonomic structure of soil organisms in minimally managed agricultural fields. Methods: We used DNA metabarcoding to investigate both terrestrial prokaryotes and eukaryotes in cabbage-cultivated and uncultivated sites in a minimally managed agricultural field in central Japan from February to August 2021. Analyses of the relative abundances of prokaryotic and eukaryotic sequence variants (SVs) and their β-diversities, and the subsequent redundancy analysis (RDA) clarified the dynamic changes in eukaryotic communities during cultivation. We further investigated taxonomic changes in fungi-, protist-, and animal-derived SVs, abundant SVs in each eukaryotic phylum, as well as the co-occurrence networks of the top 150 SVs. Results: The results revealed that the fractions of predatory or parasitic protists and animals increased, whereas those of fungi and earthworm *Enchytraeus* spp. decreased. The fractions of abundant SVs derived from diatoms, Ciliophora, the class Vampyrellidae (Cercozoa), and mites increased and subsequently decreased during this period. These findings suggest that predatory protists and animals fed on bacteria and autotrophic eukaryotes (such as diatoms) propagated in spring, followed by their propagation and parasitism to host eukaryotes. The networks also changed, especially prokaryotic networks that markedly changed from April to May, and those of eukaryotes from May to June–August, supporting the observations mentioned above. Conclusions: These findings indicate the dynamic and sequential changes in soil communities in fields with minimal agricultural practices and could be useful for sustainable natural farming.

## 1. Introduction

Most terrestrial organisms are invisible but greatly contribute to maintaining the integrity of soil ecosystems via nutrient recycling and biological interactions. Previous studies have shown that the taxonomic composition and diversity of soil organisms, mainly microorganisms, are influenced by the physical and chemical parameters of soil, including nutrients [1], climate [2], biological interactions (including plants [3]), soil management techniques [4], soil properties, and land use [5] (including agricultural fields). In particular, changes in soil organisms caused by agricultural practices such as fertilization and cropping have been shown to influence the growth and health of plants [6,7]. For instance, biased crop cultivation, such as continuous mono-cropping, leads to imbalanced compositions of soil organisms, which increases the risk of plant diseases in agricultural fields [8,9]. Therefore, accurate monitoring of soil biota is important for healthy crop cultivation in agricultural lands to maintain and/or regulate soil organisms that are suitable for plant growth. Although soil diagnosis based on chemical parameters has been provided by agricultural companies, the conventional diagnosis of the biological status of soils has not yet been well established. DNA metabarcoding is a useful tool for this purpose because it allows the generation of accurate quantitative data on soil organisms by analyzing the taxa and relative abundance of organisms at the nucleotide sequence level [10,11]. Many studies have clarified taxonomic changes in soil organisms in various types of agricultural soils using DNA metabarcoding [12]. We previously applied 18S ribosomal RNA (rRNA) gene-derived amplicon sequencing using Illumina MiSeq to analyze soil nematodes and successfully clarified nematode communities in sweet potato-cultivated fields [13]. Using DNA metabarcoding, we further investigated both prokaryotes and eukaryotes living in two agricultural fields with different histories of maize and cabbage cropping by rotation in central Japan in 2019, clarifying the communities in detail and the biological features of the two agricultural fields with different management histories [14].

Organic cropping utilizes natural resources, such as green manure and/or natural farming, with minimal agricultural practices (including tillage and fertilizer supply), and has recently gained attention in sustainable agriculture [15]. Several studies have profiled soil biota in conventionally crop-cultivated fields, including ours; however, the biota and their dynamics in cropping fields under limited agricultural management and practices have been poorly investigated. We think that clarifying what organisms live in agricultural soils with minimal agricultural practice and how their communities change during crop cultivation is very important to understand crop–soil organism interactions, which are useful for improved organic or natural farming. Thus, from November 2020 to August 2021, we cultivated cabbage in a field without agricultural practices, except for the initial fertilizer supply, and successfully clarified the dynamics of prokaryotic and eukaryotic communities and their co-occurrence networks in the field soils for half a year (February–August 2021) using DNA metabarcoding. These findings will help us understand the communities and their dynamics in minimally managed field soils and provide us with hints to effectively perform natural farming.

## 2. Materials and Methods

### 2.1. Soil Sampling Site

**Figure 1:**
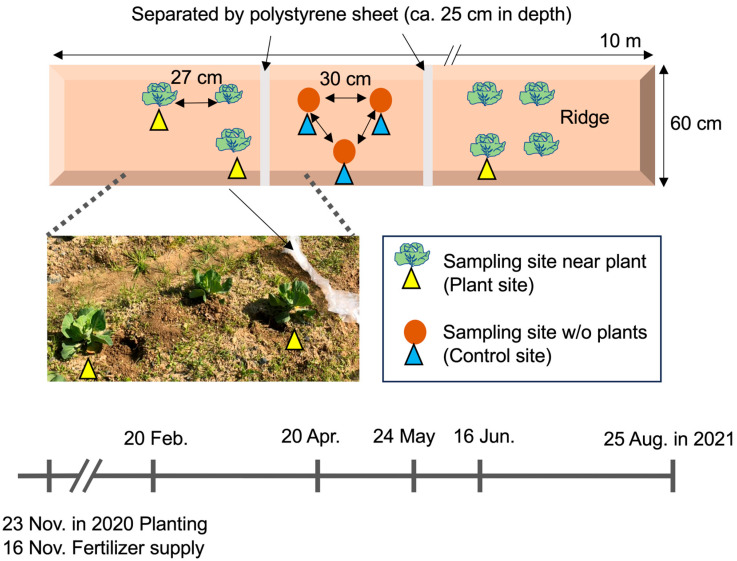
Experimental field sites and schedule of soil sampling. Cabbage was cultivated in the field in the campus of Toyohashi University of Technology from November 2020 to August 2021. The cultivated area was separated from the uncultivated area by a polystyrene sheet (approximately 25 cm in depth). Chemical fertilizer was only supplied to tilled soils prior to cultivation. Soils were isolated from the three sampling sites near the crops (plant sites) and three sampling sites in the unplanted area (control site) on the indicated dates (20 February, 20 April, 24 May, 16 June, and 25 August). The photo was taken on 20 April 2021.

This study was performed in a field managed by the Research Center for Agrotechnology and Biotechnology at the Toyohashi University of Technology campus in Toyohashi City (Aichi Prefecture), central Japan (34.70° N, 137.41° E). The field was used for sweet potato cultivation in 2017 [13] and then remained uncultivated. Cabbage (*Brassica oleracea*) was cultivated from November 2020 to August 2021. The field’s soil was relatively silty and contained stones, and the field was tilled and furrowed before cropping. Cabbage was cultivated and grown on a ridge (27 cm in distance; 60 cm × 10 m), and control sites without plants were prepared on the same ridge. The sampling sites for planted and unplanted (control) samples were separated by polystyrene sheets buried approximately 25 cm deep on 5 March 2021 (Figure 1). Chemical fertilizer (Toyotane Co, Ltd., Toyohashi, Japan) was supplied to the tilled soils on 16 November 2020 before cabbage cultivation (nitrogen, P_2_O_5_, and K_2_O at 0.75 kg/m^2^). Cabbage seedlings were planted on 23 November 2020, and plants were continuously cultivated under climatic conditions without fertilizer, water supply, pesticides, or weeding until 25 August. Surface soils near three independent plants (within 10 cm distance from the plants and 15 cm depth; that is, the plant site) and at three unplanted sites (that is, the control site) were sampled on 20 February, 20 April, 24 May, 16 June, and 25 August under clear climatic conditions. Sampling intervals were determined to analyze the changes in soil biota from early spring to mid-summer. A total of 30 sample soils were isolated from 3 independent sampling points of the control and plant sites on five sampling date and pretreated before DNA purification, as described previously [13].

### 2.2. Soil DNA Purification and Amplicon Sequencing

Purification of soil DNA and subsequent amplicon sequencing were carried out as described in our previous study [14], with slight modifications. Briefly, whole-soil DNA was purified from 10 g of fresh soil using a DNeasy PowerMAX Soil Kit (QIAGEN, Venlo, The Netherlands), and 400-μL aliquots were concentrated to 50 μL in TE buffer (pH 8.0) via ethanol precipitation with 40 μL of 3 M sodium acetate (pH 5.2) and 1 mL ethanol. Purified soil DNA was stored at −20 °C. The 16S (V3–V4 region) and 18S (V7–V8 region) ribosomal RNA (rRNA) genes were amplified from the soil DNA using the universal primers 16S_Amplicon_MiseqF and 16S_Amplicon_MiseqR (341F and 805R with tail sequences) and F1183-18S_V7-V8_MiseqF and R1631a-18S_V7-V8_MiseqR, respectively. The PCR mixture (25 μL) contained 12.5 μL of 2 × Buffer for KOD FX Neo, 5 μL of 2 mM dNTPs, 0.5 units of KOD FX Neo DNA polymerase (Toyobo, Tokyo, Japan), 2 μL of template DNA, and 0.3 mM each of the forward and reverse primers. Amplification was initiated with denaturation at 94 °C for 2 min followed by 30 cycles of denaturation at 94 °C for 10 s, annealing at 55 °C for 30 s, and extension at 68 °C for 60 s. Amplified PCR products were purified with an equal volume of RNAClean XP beads (Beckman Coulter, Brea, CA, USA), and the beads were washed with 170 mL of 80% ethanol twice and eluted with 20 mL of distilled water. Index PCR was performed in eight cycles using a Nextera XT Index Kit v2 (Illumina, San Diego, CA, USA). The amplified libraries were purified using RNAClean XP beads, as described above, and eluted with 10 mM Tris-HCl (pH 8.5). Library concentrations were quantified using a spectrophotometer, and equal amounts of each library were pooled and quantified using a Qubit dsDNA HS Assay Kit (Thermo Fisher Scientific, Waltham, MA, USA). Each 250 bp end of the pooled library was sequenced using a MiSeq Reagent Kit v2 (500 cycles; Illumina) on a MiSeq instrument (Illumina). Sequences were deposited in the DDBJ Sequence Read Archive (DRA) database under the accession number DRR582878–DRR582937.

### 2.3. Sequence Data Analysis

The sequence data of the 16S and 18S rRNA genes from 60 amplicons were independently imported into QIIME2 version 2023.9 [16] and the primer sequences were removed using the Cutadapt plugin (version 4.5) [17] with default parameters. Forward and reverse reads were joined, denoised, and chimera checked using the dada2 plugin [18]. The resultant sequence variants (SVs) of 16S and 18S rRNA genes (prokaryotic and eukaryotic SVs, respectively) were further processed using the vsearch (version 2.22.1) [19] uchime ref command with a minimum score option of –min 0.5, for the removal of chimeric sequences. The taxonomic assignment of the SVs was based on the SILVA database (version 138) [20] with a 99% clustering threshold. Finally, 16S and 18S rRNA gene-derived SVs <400 and <407 bp in length, respectively, were removed, and the remaining SVs were used for further analyses.

The phylum-level compositions of the SVs (>0.5% of the total reads) were displayed as histograms for each sample using the R packages phyloseq (version 1.46.0) [21] and ggplot2 (version 3.5.0). Line graphs of the abundant SVs (top 10 in abundance) for each phylum were prepared using Microsoft Excel for investigating the changes in their relative abundances throughout the period. For α-diversity analyses for assessing the diversities in each sample, the Shannon index plot for α-diversity was obtained using plot_richness functions in the phyloseq package in R. Difference of the Shannon indexes of animal-derived SVs between two samples was investigated by Tukey–Kramer test in R. For β-diversity analyses for investigating species diversity between sample groups, the non-metric multidimensional scaling (NMDS) plot of the Bray–Curtis distance matrix for β-diversity was obtained using ordinate and plotordination functions in the R package phyloseq. The env.fit function of the vegan package was used to plot the variable vectors for each soil chemical characteristic value in the NMDS plane on which the biological community was plotted. The pairwise.adonis2 test [22] based on Bray–Curtis distance matrices was performed to determine whether there were significant differences between two consecutive sample groups. Prokaryotic and eukaryotic networks of top 150 abundant SVs of each sample group were obtained using corMicro function (method = spearman; method.scale = TMM; p. threshold = 0.01; r. threshold = 0.8 and 0.7 for prokaryotic and eukaryotic SVs, respectively), and clustered by Phylum using PolygonClusterG function in ggClusterNet package (version 0.1.0) [23]. The resulting network diagrams were visualized using the ggplot and geom_Segmant functions for investigating the changes in network structures throughout the period. Network robustness was evaluated based on the robustness. Numbers of positive and negative links, connectance (link density), relative modularity, and network robustness were used for characterizing the networks from sample groups. Redundancy analysis (RDA) was performed using the rda function in the R package vegan (version 2.6-4) to investigate the relationship between chemical properties and prokaryotic and eukaryotic taxa in each sample.

For heatmap visualization of the chemical data in the samples, the means of measured values from the triplicates were determined in 10 samples from control and plant sites on each sampling date, and the percentage of each sample was calculated by the mean value divided by the total values of the mean values of 10 samples in each chemical parameter. A heatmap was prepared by clustering with the farthest neighbors algorism using the heatmap function in R.

Sequence identity analysis of nematode-derived SVs and abundant SVs in each eukaryotic phylum was performed using the BLASTN program against the GenBank database in May 2024 (https://blast.ncbi.nlm.nih.gov/Blast.cgi (accessed on 23 May 2024)), and the taxonomic information of the genus-identified hits with the lowest e-values was used as the taxa of those SVs. Feeding types of nematode SVs were assigned according to their closest genus, as identified by BLASTN searches based on the reference by Yeates et al. [24] and the Nematode Ecophysiological Parameter Search at the Nemaplex homepage of UC Davis, USA, in May 2024 [25]. The cp-values of the nematode families were classified as previously described by Bongers [26,27] and the nematode Ecophysiological Parameter Search [25]. The maturity index was calculated as described previously [13].

### 2.4. Soil Chemical Parameters

The following parameters were measured using triplicate sample soils dried at 60 °C overnight by the Inochio Agricultural Central Research Center (Tahara, Aichi, Japan): cation-exchange capacity, pH in water and KCl solution, nitrate nitrogen, ammonium nitrogen, exchangeable potassium, exchangeable magnesium, exchangeable calcium, Ca, K, Mg and Cl saturations, electric conductivity, available phosphorus, and humus contents.

## 3. Results

### 3.1. Prokaryotic and Eukaryotic Phylum Compositions at Control and Cabbage-Cultivated Sites During Cultivation

Cabbage cultivation was carried out from 23 November 2020 to 25 August 2021, under minimally managed conditions. The climate data (total rainfall, mean temperature, and total sunshine duration) for Toyohashi City for 2021 (Supplementary Figure S1) indicated a continuously increasing temperature from 7 °C (February) to 27 °C (August), increased rainfall in April and May, a transient decrease in June, and the highest rainfall in August. Bulk soil samples were isolated from three sites near the plants (designated as plant sites) and three unplanted sites (control sites) on the same ridge, five times from winter to summer in 2021 (Figure 1). We comparatively analyzed the soil biota from both sites to investigate the influence of plants on soil organisms and monitor the changes in the organisms in this period. We identified 4375 prokaryotic and 5165 eukaryotic sequence variants (SVs) from 30 samples isolated from the control and plant sites in triplicate in February, April, May, June, and August.

**Figure 2:**
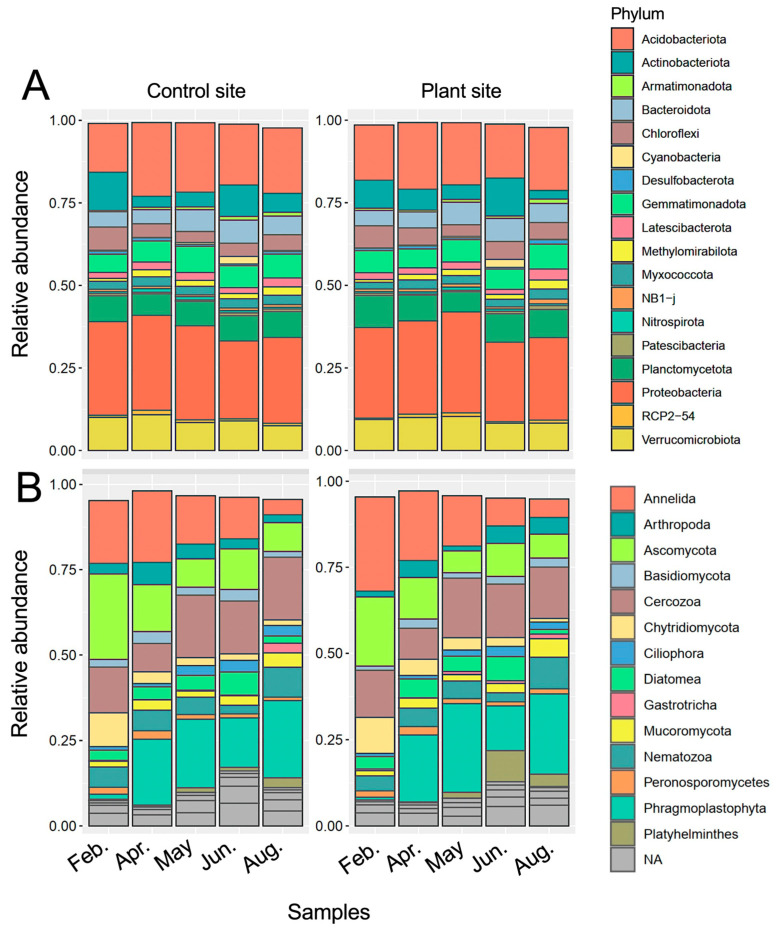
Relative abundance of prokaryotic and eukaryotic phyla in the samples. Relative abundance of prokaryotic (**A**) and eukaryotic (**B**) phyla from unplanted (control) and cabbage-cultivated (plant) sites on five different sampling dates is shown. Phyla are based on the SILVA database and indicated by colors as shown in the right of figures. NA: not assigned phylum.

Phylum compositions of prokaryotes and eukaryotes at the control and plant sites during cultivation are shown in Figure 2A,B, respectively. The prokaryotic phylum compositions at both sites were comparable and showed similar changes throughout the study period. The relative abundances of abundant SVs from two major phyla, Acidobacteriota and Proteobacteria, increased in April and then decreased, and Actinobacteriota-derived abundant SVs tended to exhibit contrasting profiles (Supplementary Table S1; Supplementary Figure S2A–C). Cyanobacteria-derived SVs were abundant in June (Supplementary Figure S2K). The eukaryotic phylum compositions of the samples markedly changed over time; for example, the fractions of Annelida decreased and those of Phragmoplastophyta increased in total eukaryotes during cultivation (Figure 2B). The relative abundances of fungi, protists, and animal-derived SVs were further investigated in the samples (Supplementary Figure S3). In fungi, the fractions of the phylum Ascomycota were the most abundant in total eukaryotic SVs, and the increase and decrease in Mucoromycota- and Chytridiomycota-derived SVs, respectively, were notable. SVs derived from the phyla Cercozoa and Annelida were the most abundant among protist- and animalia-derived SVs, respectively (Figure 2B). The phylum compositions of fungi- and protist-derived SVs were comparable between the samples from the control and plant sites, whereas those of animals, such as Annelida, changed differently between sites (Supplementary Figure S3). A detailed taxonomic analysis of eukaryotic SVs is described in the following section.

### 3.2. α- and β-Diversities of Samples in Taxonomic Groups

**Figure 3:**
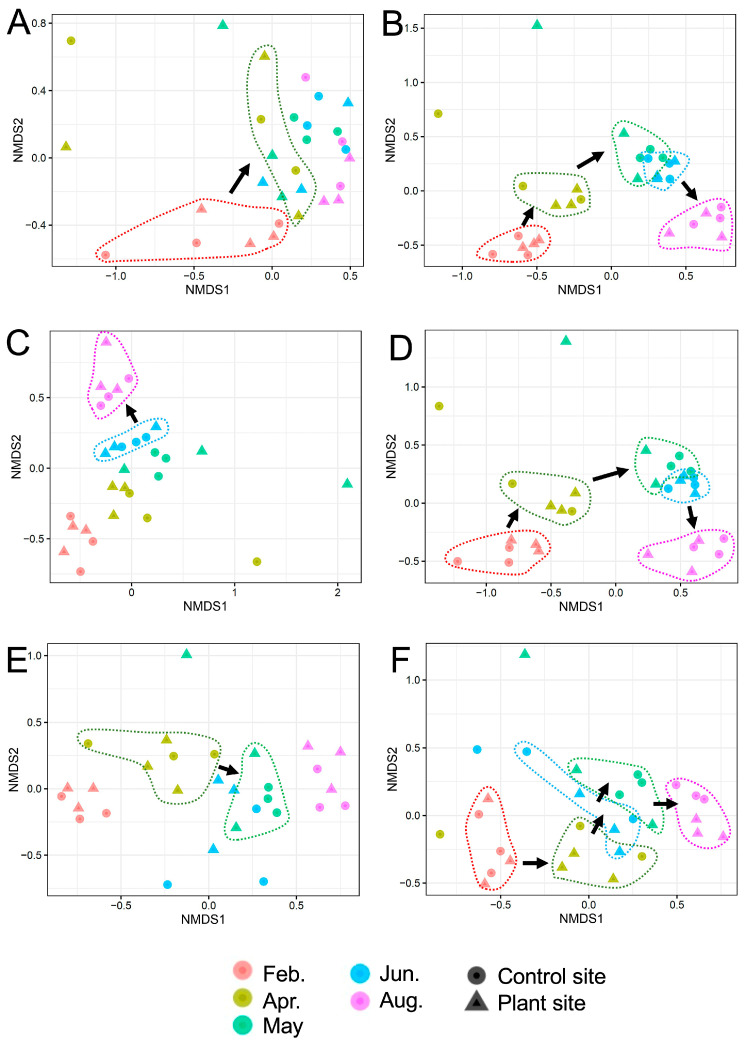
Nonmetric multidimensional scaling (NMDS) ordinations showing differences in β-diversity. The NMDS plots are based on Bray–Curtis dissimilarity among the communities of prokaryotes (**A**), eukaryotes (**B**), fungi (**C**), protists (**D**), animals (**E**), and nematodes (**F**) in the samples. Two sample groups of clustered plots with significant difference by pairwise.adonis2 test (*p* adjusted < 0.05) are indicated by dotted lines and an arrow for time course (see Supplementary Table S3).

We further analyzed changes in α-diversities based on the Shannon index and β-diversities based on the Bray–Curtis distance in the samples. The Shannon indices for prokaryotes, eukaryotes, fungi, protists, animals, nematodes, and plants are shown in the Supplementary Figure S4A–G, respectively). The α-diversity of nematodes in animals was analyzed because they represent a suitable indicator for assessing the soil environment [28]. The indices changed differently among taxonomic groups, and differences in the indices were also found between the control and plant sites. The Shannon indices in six groups, except for plants, tended to increase during the period, especially those in animals at plant sites, and significantly increased from February to August (Tukey–Kramer test, *p* < 0.01) (Supplementary Table S2, Supplementary Figure S4E). In particular, the Shannon indices of plant-removed eukaryotes showed an increase in eukaryotic SVs (Supplementary Figure S4H). The β-diversity plots of prokaryotes, eukaryotes, fungi, protists, animals, and nematodes are shown in Figure 3A–F, respectively. Unlike the prokaryotic plots assigned to two groups (February and others) (Figure 3A), those of eukaryotes, fungi, protists, and animals were separated into 2–5 clusters based on the cultivation period (Figure 3B–E). The Bray–Curtis distances of several pairs of consecutive samples were found to be significantly different by the pairwise.adonis2 test (*p* adjusted < 0.05) (Supplementary Table S3). In Figure 3, these sample groups are surrounded by dotted lines and an arrow, indicating the change in the corresponding communities. These data suggest that eukaryotic communities such as fungi, protists, and animals significantly change during cultivation.

### 3.3. Redundancy Analysis (RDA) of the Sample Groups and Soil Chemical Parameters

**Figure 4:**
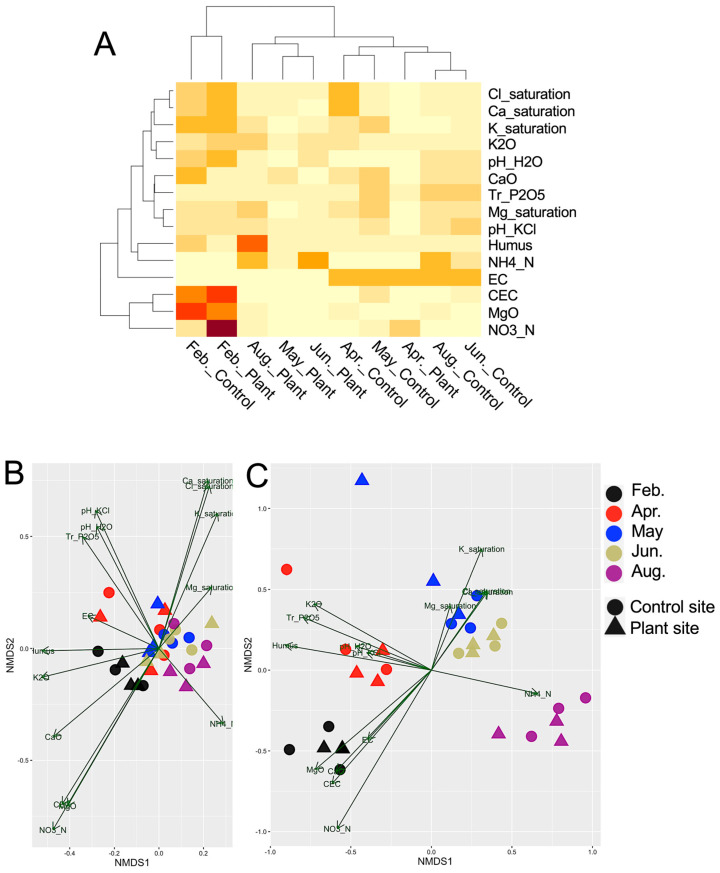
Heatmap of soil chemical parameters and redundancy analysis (RDA) of the samples. (**A**) Heatmap of soil chemical parameters in the samples. Samples are clustered by the relative contents of chemical parameters. Each parameter and the sample with its corresponding sampling site are indicated on the right and bottom of the map, respectively. Abbreviations of chemical parameters: cation-exchange capacity (CEC), pH in water and KCl solution (pH_H2O, pH_KCl), nitrate nitrogen (NO3_N), ammonium nitrogen (NH4_N), exchangeable potassium (K2O), exchangeable magnesium (MgO), exchangeable calcium (CaO), electric conductivity (EC), available phosphorus (Tr_P2O5), and humus content (Humus). (**B**,**C**) Redundancy analysis (RDA) of prokaryotic (**B**) and eukaryotic (**C**) communities in the samples showing soil chemical properties as vectors. The colors and symbols for samples and sampling sites are shown on the right of the figure. Longer arrows indicate a stronger association.

The heatmap of 15 chemical parameter values in the soil samples indicated high nitrite nitrogen, exchangeable magnesium, and cation-exchange capacity in February’s soils, presumably because of the initial fertilizer supply prior to cultivation (Figure 4A). To investigate the association of the samples with the chemical parameters, the five sample groups were clustered according to the chemical parameters determined by RDA, and the resultant plots of prokaryotic SVs were not associated with any parameters (Figure 4B). In contrast, the plots of eukaryotic SVs indicated the associations of the SVs in February with the above parameters whose contents were high in February’s soils; those in April with phosphorus and humus contents; those in May and June with potassium, magnesium, and chloride saturations; and those in August with the ammonium nitrogen content (Figure 4C), also suggesting dynamic changes in eukaryotic communities.

### 3.4. Changes in Relative Abundances of SVs in Major Eukaryotic Groups and Abundant SVs in Phyla During Cultivation

As changes in eukaryotic communities were suggested, taxonomic changes in major eukaryotic groups (such as fungi, protists, animals, plants, and nematodes) were investigated. The class compositions of fungal SVs in the phyla Ascomycota, Basidiomycota, Chytridiomycota, and Mucoromycota are shown in Supplementary Figure S5A–D, respectively. Most Basidiomycota- and Chytridiomycota-derived SVs were assigned to the Agaricomycetes and Chytridiomycetes classes, respectively. Many Ascomycota- and Mucoromycota-derived SVs were classified as Dothideomycetes, Sordariomycetes, and Glomeromycetes (Supplementary Figure S5A,D). The fractions of Ascomycota- and Chytridiomycota-derived SVs decreased, and those of Mucoromycota-derived SVs increased during this period. The β-diversity analysis of three fungal phyla-derived SVs (except for Chytridiomycota) showed three clusters of samples (February, April, April/May/June, and August) (Supplementary Figure S5E,F,H), suggesting three distinct communities in each phylum during winter (February), summer (August), and the intermediate season (April–June). The changes in relative abundances of abundant SVs in each eukaryotic phylum (listed in Supplementary Table S4) were also investigated and the SVs were classified to four groups (“increased”, “decreased”, “increased and then decreased”, and “decreased and then increased”) based on the changing profiles throughout the period (Table 1). The relative abundance of Ascomycota- (SV_8, 16), Chytridiomycota- (SV_13), and Cryptomycota-derived SVs rapidly decreased during cultivation (Supplementary Figure S6A,C,G; Table 1) and those of several abundant SVs from Basidiomycota, Aphelidea, LKM15, and Blastocladiomycota transiently increased in May and June (Supplementary Figure S6D–F,H).

Protists, which consist of predators and parasites including plant pathogens and protozoan SVs, were derived from two major kingdoms, Rhizaria and Stramenopiles, and from two minor kingdoms, Alveolata and Amoebozoa. The relative abundance of Alveolata-derived SVs continuously increased (Supplementary Figure S7A). In β-diversity analysis, protist SVs from each kingdom were separated into four sample groups (February, April, May/June, and August) (Supplementary Figure S7B–E). The fractions of SVs derived from the classes Conoidasida in Apicomplexa, Intramacronucleata in Ciliophora, and Vampyrellidae in Cercozoa increased in all eukaryotes at the control and plant sites (Supplementary Figure S7F). In addition, the fractions of Phytomyxea-derived SVs containing clubroot disease pathogens decreased from February to May and reached constant levels in June and August (Supplementary Figure S7G). The relative abundances of abundant SVs changed differently in the protist phyla (Supplementary Figure S8): the fractions of the most abundant protist SV (SV_3) in Diatomea transiently increased in April and June, whereas those of the three Vampyrellidae-derived SVs (SV_24, 38, and 41) increased from February to June and then decreased in August (Supplementary Figure S8A,B; Table 1). The abundance of Apicomplexa-derived SV_36 was significantly increased at the plant sites (Supplementary Figure S8D; Table 1).

In animals, the fractions of the most abundant earthworm SV_1 markedly decreased, especially at plant sites; those of three abundant SVs (SV_14, 29, and 35) in Arthropoda transiently increased in April–May and then decreased, and the fractions of Gastrotricha- and Platyhelminthes-derived abundant SVs tended to increase over time (Supplementary Figure S9A,B,D,E; Table 1). The relative abundances of abundant plant (Phragmoplastophyta)-derived SVs (SV_2, 4, and 11), except for SV_6, increased with cultivation, and cabbage-derived SV_4 was predominantly detected at plant sites (Supplementary Figure S9F).

The relative abundance of nematode SVs at the genus level was further investigated, which indicated clear taxonomic changes during this period (Supplementary Figure S10A). For instance, the fractions of SVs from the genera *Acrobeloides* and *Plectus* markedly decreased, especially in May, whereas those of *Prismatolaimus*- and *Dorylaimellus*-derived SVs increased in August. SVs derived from the genera *Mononchus* and *Prionchulus* were dominant in May. The feeding habitat of nematodes and maturity index are useful indicators for assessing nematode communities [26,27]. The feeding types and the colonizer–persister (cp)-values of nematode SVs were determined based on the genus closest to nematode SVs determined by a BLASTN search (Supplementary Table S5). The fractions of plant feeders (plant parasitic nematodes) in the total nematodes markedly increased during cultivation, and those at plant sites were higher than those at control sites in August (Supplementary Figure S10B). The relative abundances of fungal feeders and omnivores tended to decrease and increase, respectively, whereas that of predators increased significantly in May. The maturity indices of the samples were calculated using cp-values, and the frequencies of nematode genera were comparable at the control and plant sites and gradually increased throughout the study period (Supplementary Figure S10C), suggesting an increase in nematodes with long generation times and low reproduction rates [27] in the population.

### 3.5. Changes in Prokaryotic and Eukaryotic Co-Occurrence Networks During Cultivation

**Figure 5:**
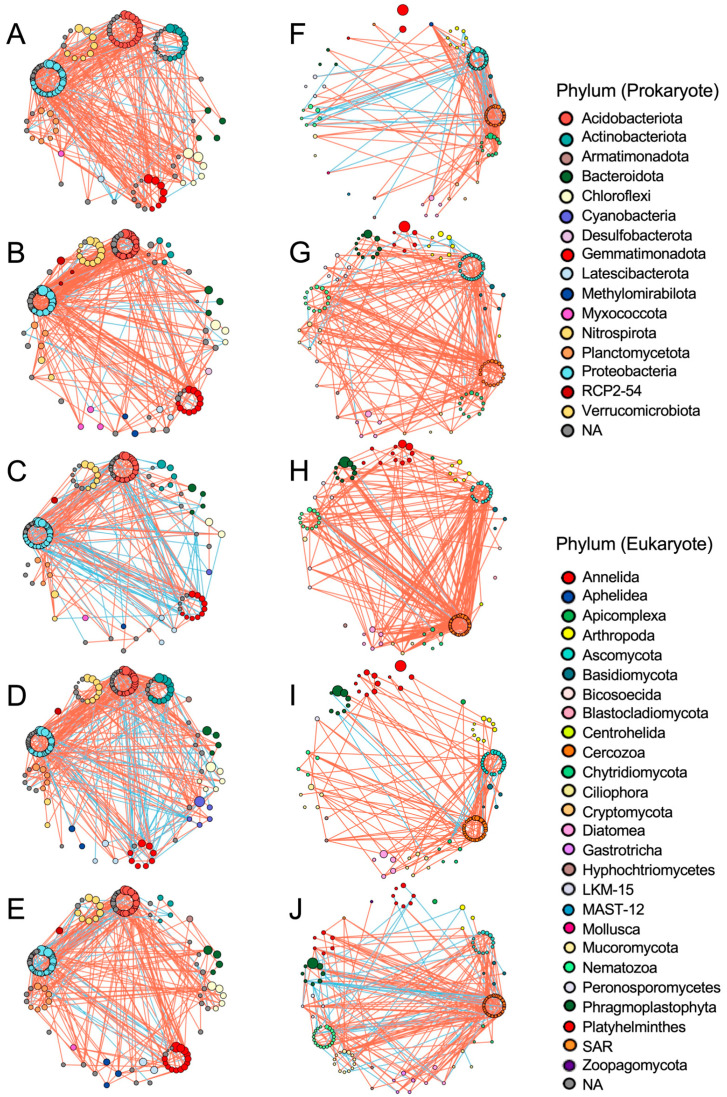
Networks of prokaryotic and eukaryotic SVs in each sample group. Co-occurrence networks of the top 150 abundant prokaryotic (**A**–**E**) and eukaryotic (**F**–**J**) SVs were prepared based on Spearman’s correlation coefficient from the samples isolated in February (**A**,**F**), April (**B**,**G**), May (**C**,**H**), June (**D**,**I**), and August (**E**,**J**). Node SVs in each phylum are indicated as colored circles and size of circles reflects its abundance. Phylum groups of node SVs are arranged clockwise. Red and blue lines indicate positive and negative correlations between node SVs. Colors for prokaryotic and eukaryotic phylum are shown in the right of the figure. NA: not assigned phylum.

We further analyzed the co-occurrence networks of the top 150 most abundant prokaryotic and eukaryotic SVs during cultivation (Figure 5; Supplementary Table S6). Node SV-formed networks were clustered and shown by their phyla, and positive and negative links are indicated by red and blue lines, respectively. In prokaryotic networks, several node SVs derived from the phyla Acidobacteriota, Gemmatimonadota, Proteobacteria, and Verrucomicrobiota were consistently observed in the five networks (Figure 5A–E). Despite the consistent dense connections between Acidobacteriota- and Proteobacteria-derived node SVs, the network structures and taxonomic compositions of the node SVs differed among the samples. For example, the number of node SVs derived from Actinobacteriota, Chloroflexi, and Planctomycetota varied in the networks and commonly decreased in the April network (Figure 5B). Cyanobacteria-derived node SVs appeared only in May and June, and Nitrospirota- and Myxococcota-derived node SVs were absent in February and August, and June, respectively. Negative and positive links suggest competition and cooperation between the linked node SVs. The numbers and connections of prokaryotic positive and negative links obviously changed during this period; the numbers of negative links markedly increased in the networks of May and June (Figure 5C,D; Supplementary Table S6). The network of April had the largest number of positive links and the smallest number of negative links (Supplementary Figure S11A), with triangular positive links among the phyla Acidobacteriota, Gemmatimonadota, and Proteobacteria being dominant (Figure 5B); however, after a month, May’s network was formed by the smallest number of node SVs and contained the largest and smallest number of negative and positive links, respectively (Figure 5C; Supplementary Table S6), indicating obvious changes in prokaryotic networks from April to May.

In eukaryotes, the network structures changed as observed in prokaryotic networks (Figure 5F–J), but the profiles of the changes in both networks are distinct. Changes in link numbers and connectance (link density) were different between the prokaryotic and eukaryotic networks (Supplementary Figure S11A–C). Negative links in the eukaryotic networks were dominant in winter (February) and summer (August). Unlike the prokaryotic networks, the eukaryotic network in May contained the largest number of links with the largest positive and the smallest negative links (Supplementary Table S6), and many positive links were formed between Cercozoa and other phylum-derived node SVs (Figure 5H). Cercozoa-derived node SVs also formed several links with other node SVs in the other four networks, suggesting the involvement of Cercozoa in the formation of eukaryotic networks. Abundant links between Ascomycota- and Cercozoa-derived node SVs were consistently found, while links from other phylum-derived node SVs varied in the networks (Figure 5F–J). Plant-derived node SVs increased in spring (February–May) and were consistently present in the networks of June and August. Interestingly, eukaryotic networks were densely formed from February to May by increasing the number of positive links approximately 2.5-fold (Supplementary Figure S11B); however, the number of links markedly decreased in the June network, followed by the August network with increased negative links (Figure 5I,J). The relative modularity of prokaryotic networks was the highest and lowest in the eukaryotic networks in April and June, respectively, whereas that of eukaryotic networks was relatively constant during this period (Supplementary Figure S11D). Additionally, network robustness was estimated by random removal of prokaryotic and eukaryotic node SVs. The resultant curves of eukaryotic node SVs decreased similarly among the samples, but those of prokaryotic node SVs were relatively variable, and the robustness in April was much higher than that in August (Supplementary Figure S11E,F).

## 4. Discussion

Recent progress in DNA sequencing has allowed us to analyze organisms living in the geosphere using DNA metabarcoding, and prokaryotic and/or eukaryotic communities have been identified in various types of soils, including agricultural soils [10,11,12]. In this study, we investigated the taxonomic composition and dynamics of soil prokaryotes and eukaryotes during cabbage cultivation under unmanaged conditions (no additional fertilizers, water supply, pesticides, or weeding) at planted and unplanted control sites. In addition, it should be noted that our results were obtained from the samples isolated from two blocks (control and plants) in an unmanaged field alone without comparing with a managed field.

In prokaryotes, we found relatively consistent phylum compositions during cultivation (Figure 2A); however, prokaryotic networks changed dynamically (Figure 5A–E), indicating that prokaryotic networks can be easily altered by climate and/or soil environmental factors, such as soil nutrients, temperature, and moisture, as previously observed [2]. This is consistent with our previous finding that soil prokaryotic networks are altered by different crops and fields under crop rotation [14]. Interestingly, the contrasting prokaryotic networks of April and May/June indicated that the bacterial communities changed rapidly from April to May (Figure 5B–D). The networks in May and June contained an increased number of negative links, indicating potential competition among soil bacteria. These findings could be explained by competition for nutrients. The nutrients supplied as fertilizer prior to cropping in winter were consumed for propagation by bacteria and other eukaryotes, such as diatoms and plants, in spring, and the propagated bacteria likely competed for the remaining nutrients in May–June (Figure 4A). We found several seasonal changes in the abundance of several SVs; for example, phototrophic cyanobacteria-derived SVs increased in abundance in May and June, presumably due to increased light, rainfall, and suitable temperature, and the propagated cyanobacteria might serve as nutrient resources for other organisms [29] (Supplementary Figure S2K). We also found that the relative abundances of abundant SVs derived from the phyla Proteobacteria and Actinobacteriota and those of Actinobacteriota changed from February to April (Supplementary Figure S2A–C); however, the reasons for this remain unknown.

Changes in the relative abundance of SVs are more notable in eukaryotes than in prokaryotes from winter to summer, which is consistent with the seasonal changes in eukaryotes in rewetted fen peatlands, as observed by Wang et al. [30]. Plant-derived abundant SVs became markedly dominant in eukaryotes during cultivation and were derived from growing weeds and cabbage (SV_4) in spring (Figure 2B; Supplementary Figure S9F). We classified several abundant SVs in each eukaryotic phylum into four groups based on the changing profiles of relative abundance during the study period (Table 1) and investigated their feeding habitat and life strategies based on the closest genera (Supplementary Table S4). The SVs in the “increased” group contain three fungal and protist and seven animal abundant SVs. Among these, two fungal SVs were assigned to parasitic fungi to plants (SV_90) [31] and amoeba (SV_407) [32], two protist SVs (SV_36 in Apicomplexa and SV_169) are potentially parasitic to other eukaryotes including animals [33,34], one nematode SV (SV_32) was assigned to plant parasite *D. parvulus*, and four SVs were classified as the predatory flagella-feeding protist *B. petiolata* (SV_614) [35] and three ciliate feeder *Stenostomum*-derived SVs (SV_71, 99, 100) [36]. These eukaryotes are likely propagated by parasites in host organisms or by feeding on prey propagated during cultivation. The abundant SVs in the “decreased” group contained many fungal SVs, especially SVs derived from Chytridiomycota and Cryptomycota (Table 1). The observed decrease in the relative abundance of fungal SVs may be explained by the relatively increased abundance of other eukaryotes, including plants propagated from spring to summer, as the absolute abundance of fungi is relatively constant throughout the year [37]. In addition, predation by fungal feeders such as protists and nematodes propagated in spring could cause decreased fungal fractions in eukaryotes. Although there are a few protists and animal SVs in this group, the most abundant SV_1 belongs to this group, and SV_1 was assigned to the earthworm *E. dichaetus*, which has been detected in Korea [38] and the Russian Far East [39]. This observation is consistent with seasonal changes in earthworms (the abundance of earthworms is the highest in spring and decreases in autumn) [40]. Interestingly, we found that the relative abundance of SV_57, assigned to the pathogenic protist genera *Pythium* [41] and *Globisporangium* [42] in Peronosporomycetes, decreased during cultivation (Supplementary Figure S8C). We also observed decreased fractions of the protist class Phytomyxea-containing the clubroot disease pathogen [43,44] (Supplementary Figure S7G). These protists infect and propagate in the host plants. It remains unclear why these pathogenic protists propagate poorly in minimally managed field soils despite the presence of host plants. Repeated cultivation of Brassicaceae plants increases the risk of expanding clubroot disease [43] via infected root residues, and the accumulation and propagation of pathogens via host infection may be insufficient to cause disease in cabbage during the given period.

The relative abundances of several abundant SVs in the phyla increased and subsequently decreased in the soil eukaryotes during cultivation. Four diatom (autotrophic microalgae)-derived SVs (SV_18, 30, 103, and 106) belong to the “increased and then decreased” group (Table 1). Because Foets et al. showed that the abundance of diatoms is influenced by soil moisture [45], it is not surprising that the fractions of these SVs increased during the rainy season around June and then decreased in summer (Supplementary Figure S8A). Two mite-derived SVs (SV_14 and 35) in Arthropoda belong to this group, which is consistent with the seasonal changes in mites observed by Bedano [46]. The abundant SVs derived from the protist class Vampyrellidae (SV_24, 38, and 41) in Cercozoa and the phylum Ciliophora also belong to this group. Protoplast-feeding protozoa belong to the class Vampyrellidae [47]. SV_24 and SV_41 are the closest to the nematode feeder *T. weberi* [48] and fungus feeder *A. impatiens* [49], respectively, and SV_38 was assigned to the Closterium feeder *V. closterii* [50]. Two Ciliophora-derived SVs (SV_116 and 159) were assigned to *O. georgiana*, which feeds on cyanobacteria and green algae [51]. Two other SVs in this group, SV_42 and SV_101, were assigned to the nematode predator *Mononchus* [52] and the earthworm predator *A. triangulatus* in Platyhelminthes [53], respectively (Table 1). The abundances of these predatory protists and animals are likely influenced by those of their prey (fungi, protists, nematodes, and earthworms), which can rapidly propagate by feeding on prey when they are abundant in soils, and the subsequent depletion of prey decreases their relative abundances. Thus, the abundant SVs in the “increased and then decreased” group were further classified into two types: the SVs whose relative abundances are mainly governed by seasonal environmental factors (climate, temperature, or rainfalls), such as diatoms, and the SVs whose relative abundances depend upon the amount of available food, such as predatory eukaryotes.

Nematode communities represent the changes in eukaryotes under limited agricultural management. In February, bacterial and fungal feeders of nematodes were abundant under bacteria- and fungi-rich conditions formed by the initial fertilizer supply, and these nematodes were fed by predators such as *Mononchus* spp. in May; plant feeders then dominated the nematode community by coupling with the propagation of plants (weeds and cabbage) in June and August (Supplementary Figure S10A,B). Increased maturity indices throughout the period indicate the increased ratio of nematodes belonging to “persisters” in the nematode population [27], which are alive under food-poor conditions presumably caused by no fertilizer supply.

We detected enhanced changes in the relative abundance of eukaryotic SVs at the cabbage cultivation sites. For instance, the fungal SV_47 assigned to *O. brassicae* [54], which parasitizes on Brassicaceae plants, exhibited an enhanced “increased and then decreased” profile of relative abundance at the plant site compared with control site (Supplementary Figure S6C), which can be explained by the propagation of this fungus in the cabbage. Interestingly, SV_147, derived from LKM15, also exhibited plant-biased changes in relative abundance (Supplementary Figure S6F), suggesting the presence of a potential parasitic fungus in cabbage. The most abundant SV_1 assigned to *E. dichaetus* was detected abundantly at the plant site, presumably due to the abundant distribution of foods around crops. This biased distribution of earthworm may account for plant-biased change in SV_101, assigned to earthworm predator *Arthurdendyus triangulates* [53] (Supplementary Figure S9A,E). SV_36, derived from the parasitic phylum Apicomplexa [34], and SV_169, belonging to potential parasitic protists in Peronosporomycetes (Oomycota) [33], may also be present in this case (Supplementary Figure S8C,D).

We also investigated changes in the co-occurrence networks of the 150 most abundant eukaryotic SVs during cultivation (Figure 5F–J). The number of negative links was highest in February in winter and August in summer, and the number of positive links was the highest in May (Supplementary Figure S11B). In contrast, the prokaryotic networks contained the lowest number of positive links and the highest number of negative links in May. Negative and positive links indicate biological competition and predation for limited food, nutrients, resources, habitats, and cooperation in propagation (via parasitism or food supply), respectively. Thus, these findings suggest that eukaryotes compete for limited amounts of food (such as bacteria, fungi) in winter and propagate cooperatively in spring by feeding on propagated bacteria or autotrophic eukaryotes. Then, expanded eukaryotes likely compete for limited amounts of food in summer. Negative links were found between Nematozoa- and Ascomycota- and Cercozoa- and Ascomycota-derived node SVs in February, suggesting fungal predation by nematodes and protists (Figure 5F). Acari-derived SV_35 in Arthropoda was negatively linked to four fungal (SV_17, 49, 88, and 408), two Cercozoa- (SV_38 and 158), and one nematode- (SV_26)-derived node SVs, which may indicate prey-predator interactions of the soil mite and these eukaryotes (Figure 5G; “Apr” sheet in the Supplementary Dataset S1). Plant-derived SVs dominated in the eukaryotic networks after April (Figure 5G–J), suggesting the involvement of plants in forming eukaryotic networks, presumably via positive and negative interactions with plant-parasitic fungi, protists, and/or nematodes, especially in August. We found that prokaryotic and eukaryotic network structures changed during cultivation, suggesting changeable biological interactions driven by potential competition and cooperation for nutrients or food among soil organisms. Additionally, the prokaryotic network in August was less robust than those in other samples and eukaryotic networks, suggesting that prokaryotic networks might be more influenced by soil environmental factors (such as temperature) than eukaryotic networks, which are governed by close biological interactions (such as parasitism and predation) as well as environmental factors.

## 5. Conclusions

In this study, we successfully clarified the dynamic taxonomic changes in eukaryotes as well as prokaryotic and eukaryotic networks in minimally managed field soils from February to August 2021. These changes are likely driven by biological interactions, such as predation and parasitism, as well as environmental soil factors, such as nutrients, moisture, and temperature. The resultant findings on taxonomic changes and biological interactions in this study will be useful for natural farming with minimal agricultural managements.

## Figures and Tables

**Table 1:** Changes in the relative abundance of abundant SVs in each eukaryotic phylum during cultivation.

Profile of Change	Group	Abundant SV	Phylum	The Closest Hit Species	Enhanced Changes at Plant Site
Increased	Fungi	SV_165	Basidiomycota	*Alloexidiopsis calcea*	Yes
		SV_90	Mucoromycota	*Cetraspora gilmorei*	
		SV_407	Zoopagomycota	*Ramicandelaber* sp.	Yes
	Protists	SV_36	Apicomplexa	*Eimeria tropidura*	Yes
		SV_614	Bicosoecida	*Bicosoeca petiolata*	
		SV_169	Peronosporomycetes	*Achlya bisexualis*/*Thraustotheca clavata*	Yes
	Animals	SV_28	Arthropoda	*Stigmalychus* spp.	Yes
		SV_32	Nematozoa	*Dorylaimellus parvulus*	
		SV_39	Nematozoa	*Prismatolaimus* spp.	
		SV_51	Gastrotricha	*Neogossea* spp.	
		SV_71	Platyhelminthes	*Stenostomum* spp.	
		SV_99	Platyhelminthes	*Stenostomum* spp.	
		SV_100	Platyhelminthes	*Stenostomum leucops*	
Decreased	Fungi	SV_121	Aphelidea	*Aphelidium parallelum*	
		SV_8	Ascomycota	*Chaetomium globosum*/*Humicola* sp.	
		SV_16	Ascomycota	*C. globosum*/*Humicola* sp.	
		SV_43	Ascomycota	*Ascobolus* spp.	
		SV_37	Basidiomycota	*Lyophyllum* sp. etc.	
		SV_13	Chytridiomycota	*Chytridium polysiphoniae*	
		SV_65	Chytridiomycota	*Rhizophydium patellarium*/*Betamyces* sp.	
		SV_94	Chytridiomycota	*Rhizophydium* sp.	
		SV_102	Chytridiomycota	*Catenomyces persicinus*	
		SV_161	Cryptomycota	*Rozella* spp.	
		SV_327	Cryptomycota	*Vinositunica* sp./*Bullera formosensis*	
		SV_421	Cryptomycota	*Rozella* spp.	
	Protists	SV_57	Peronosporomycetes	*Pythium* spp./*Globisporangium* spp.	
	Animals	SV_1	Annelida	*Enchytraeus dichaetus*	Yes
		SV_5	Annelida	*Enchytraeus bulbosus*	
Increased and then decreased	Fungi	SV_91	Aphelidea	*A. parallelum*	
		SV_17	Basidiomycota	*Trechispora* spp.	
		SV_259	Blastocladiomycota	*Paraphysoderma sedebokerense*	Yes
		SV_47	Chytridiomycota	*Olpidium brassicae*	Yes
		SV_147	LKM15	*Podoscypha* spp.	Yes
		SV_247	LKM15	*Morellospora saccamoebae*	
	Protists	SV_307	Bicosoecida	*Trieres chinensis*	
		SV_24	Cercozoa (class Vampyrellidae)	*Theratromyxa weberi*/*Arachnula impatiens*	
		SV_38	Cercozoa (class Vampyrellidae)	*Vampyrella closterii*	
		SV_41	Cercozoa (class Vampyrellidae)	*T. weberi*/*A. impatiens*	
		SV_116	Ciliophora	*Obertrumia georgiana*	
		SV_159	Ciliophora	*O. georgiana*	
		SV_189	Ciliophora	*Colpoda* spp.	Yes
		SV_168	Centrohelida	*Triangulopteris lacunata*	
		SV_18	Diatomea	*Craticula subminuscula*/*Stauroneis acuta*	
		SV_30	Diatomea	*Hantzschia amphioxys*	
		SV_103	Diatomea	*Halamphora montana*/*Amphora montana*	
		SV_106	Diatomea	*Nitzschia palea*	
	Animals	SV_10	Annelida	*Achaeta* spp./*Hemienchytraeus* spp.	
		SV_14	Arthropoda	*Alicorhagia* spp.	
		SV_29	Arthropoda	*Bourletiella hortensis*	
		SV_35	Arthropoda	*Pergamasus* spp. etc.	
		SV_42	Nematozoa	*Mononchus truncatus*	
		SV_101	Platyhelminthes	*Arthurdendyus triangulatus*	Yes
Decreased and then increased	Protists	SV_20	Cercozoa	*Rhogostoma bowseri*	
		SV_111	Ciliophora	*Kalometopia duplicata*	
	Animals	SV_23	Nematozoa	*Plectus* spp.	
		SV_26	Nematozoa	*Acrobeloides* spp.	

Note: The abundant SVs in each eukaryotic phylum whose relative abundances increased only, decreased only, increased and then decreased, or decreased and then increased are indicated in Supplementary Figures S6, S8 and S9 for fungi, protists, and animals, respectively. The closest hits of species by BLASTN search (May 2024) were derived from Supplementary Table S4. Enhanced changes at plant site are also indicated by “Yes” based on Supplementary Figures S6, S8, and S9 by comparing the changes in the relative abundance of SVs at the control and plant sites.

## Data Availability

The original contributions presented in the study are included in the article/Supplementary Materials; further inquiries can be directed to the corresponding author.

## Supplementary Materials

The following supporting information can be downloaded at: https://www.mdpi.com/article/10.3390/genes16050482/s1, Figure S1: Total rainfall and mean temperature and total sunshine duration per month at Toyohashi city in 2021; Figure S2: Changes in the relative abundances of abundant prokaryotic SVs in each phylum; Figure S3: Phylum compositions of fungi-, protists-, and animal-derived SVs across the cultivation period; Figure S4: Boxplots showing the α-diversities by the Shannon index across the five samples; Figure S5: Taxonomic compositions and nonmetric multidimensional scaling (NMDS) ordinations of four fungal phyla-derived SVs in each sample isolated from control and plant sites; Figure S6: Changes in the relative abundances of abundant SVs in each fungal phylum; Figure S7: Taxonomic compositions and Bray–Curtis dissimilarity plots of protist-derived SVs in the samples. Figure S8: Changes in the relative abundances of abundant SVs in each protistian phylum; Figure S9: Changes in the relative abundances of animal- and plant-derived abundant SVs in each phylum; Figure S10: Compositions of nematode genus-derived SVs and feeding habitat-classified SVs in total Nematozoa-derived SVs in the samples; Figure S11: The properties of co-occurrence networks (link numbers, connectance, relative modularity, and robustness); Table S1: Abundant SVs in each prokaryotic phylum and their taxa based on the SILVA database; Table S2: Tukey–Kramer test for assessing difference of α diversity of animal-derived SVs between two sample groups; Table S3: Difference of Bray-Curtis distances of SVs between two consecutive sample groups were investigated using the pairwise.adonis2 program; Table S4: Abundant SVs in each eukaryotic phylum, their taxa based on the SILVA database, and the top hits identified by BLASTN search; Table S5: Cp-values and feeding types of nematode-derived SVs; Table S6: Parameter values of prokaryotic and eukaryotic networks of top 150 abundant SVs in each sample; Dataset S1: Edge data of the eukaryotic networks in the samples.

## Abbreviations

The following abbreviations are used in this manuscript:

SV	Sequence variant
cp value	Colonizer–persister value
NMDS	Nonmetric multidimensional scaling
RDA	Redundancy analysis
