# Supplementary material for: Dynamic Changes in Prokaryotic and Eukaryotic Communities and Networks in Minimally Managed Cabbage-Cultivated Field Soils
Source: Genes (Basel). 2025 Apr 24;16(5):482. doi: 10.3390/genes16050482 (PMC12111001; doi:10.3390/genes16050482)
Supplement: Supplementary file 1 [file genes-16-00482-s001.zip › Suppl Materials/Suppl Table_Genes_rev.pdf]

Supplementary Table S1. Abundant SVs in each prokaryotic phylum and their taxa based on the SILVA database. Abundant SVs in each prokaryotic phylum and their SILVA-based taxa are shown. NA: not assigned. The top 10 SVs in abundance are indicated in the phylum. Minor and unassigned phyla-derived SVs are omitted. SVs in bold were used for Fig. S2.

ID	Phylum	Class	Order	Family	Genus	Species
**SV_4**		Vicinamibacteria	Vicinamibacterales	Vicinamibacteraceae	NA	NA
**SV_5**		Vicinamibacteria	Vicinamibacterales	Vicinamibacteraceae	NA	NA
**SV_12**		Subgroup_22	NA	NA	NA	uncultured_Holophaga
**SV_20**		Subgroup_11	NA	NA	NA	uncultured_bacterium
**SV_26**	Acidobacteriota	Vicinamibacteria	Vicinamibacterales	Vicinamibacteraceae	NA	NA
**SV_30**		Blastocatellia	Blastocatellales	Blastocatellaceae	uncultured	uncultured_Acidobacteria
**SV_33**		Thermoanaerobaculia	Thermoanaerobacul	Thermoanaerobaculaceae	Subgroup_10	NA
**SV_42**		Vicinamibacteria	Vicinamibacterales	Vicinamibacteraceae	NA	NA
**SV_43**		Blastocatellia	Pyrinomonadales	Pyrinomonadaceae	RB41	NA
SV_49		Blastocatellia	Blastocatellales	Blastocatellaceae	JGI_0001001-H03	uncultured_bacterium
**SV_9**		Actinobacteria	Propionibacteriales	Nocardioidaceae	NA	NA
**SV_19**		Actinobacteria	Micrococcales	Micrococcaceae	NA	NA
**SV_34**		Actinobacteria	Micrococcales	Micrococcaceae	NA	NA
**SV_51**		Acidimicrobiia	Microtrichales	Ilumatobacteraceae	Ilumatobacter	NA
**SV_75**	Actinobacteriota	Actinobacteria	Micrococcales	Intrasporangiaceae	NA	NA
**SV_97**		Acidimicrobiia	IMCC26256	NA	NA	uncultured_Actinomycetales
**SV_124**		Acidimicrobiia	IMCC26256	NA	NA	NA
**SV_134**		Actinobacteria	Micrococcales	Intrasporangiaceae	NA	NA
**SV_142**		Thermoleophilia	Gaiellales	Gaiellaceae	Gaiella	NA
SV_146		Actinobacteria	Frankiales	uncultured	NA	NA
**SV_85**		Fimbriimonadia	Fimbriimonadales	Fimbriimonadaceae	NA	uncultured_bacterium
**SV_157**		Fimbriimonadia	Fimbriimonadales	Fimbriimonadaceae	NA	uncultured_bacterium
**SV_188**		Fimbriimonadia	Fimbriimonadales	Fimbriimonadaceae	NA	uncultured_bacterium
**SV_438**		Fimbriimonadia	Fimbriimonadales	Fimbriimonadaceae	NA	uncultured_bacterium
**SV_491**	Armatimonadota	Fimbriimonadia	Fimbriimonadales	Fimbriimonadaceae	NA	NA
**SV_560**		Chthonomonadetes	Chthonomonadales	NA	NA	uncultured_bacterium
**SV_599**		Fimbriimonadia	Fimbriimonadales	Fimbriimonadaceae	NA	NA
**SV_647**		Fimbriimonadia	Fimbriimonadales	Fimbriimonadaceae	NA	uncultured_bacterium
**SV_702**		Fimbriimonadia	Fimbriimonadales	Fimbriimonadaceae	NA	NA
SV_798		Fimbriimonadia	Fimbriimonadales	Fimbriimonadaceae	NA	uncultured_bacterium
**SV_8**		Bacteroidia	Chitinophagales	Chitinophagaceae	uncultured	uncultured_Bacteroidetes
**SV_29**		Bacteroidia	Chitinophagales	Saprospiraceae	uncultured	NA
**SV_58**		Bacteroidia	Chitinophagales	Chitinophagaceae	uncultured	NA
**SV_128**		Bacteroidia	Chitinophagales	Saprospiraceae	uncultured	NA
**SV_191**	Bacteroidota	Bacteroidia	Cytophagales	Microscillaceae	Chryseolinea	uncultured_bacterium
**SV_194**		Bacteroidia	Cytophagales	Hymenobacteraceae	Adhaeribacter	uncultured_soil
**SV_230**		Bacteroidia	Chitinophagales	Saprospiraceae	uncultured	NA
**SV_241**		Bacteroidia	Chitinophagales	Chitinophagaceae	Aurantisolimonas	uncultured_bacterium
**SV_251**		Bacteroidia	Sphingobacteriales	AKYH767	NA	metagenome
SV_257		Bacteroidia	Cytophagales	Hymenobacteraceae	Adhaeribacter	uncultured_soil
SV_442		Bdellovibrionia	Bdellovibrionales	Bdellovibrionaceae	OM27_clade	uncultured_bacterium
SV_499		Bdellovibrionia	Bdellovibrionales	Bdellovibrionaceae	OM27_clade	uncultured_Myxococcales
SV_1059		Bdellovibrionia	Bdellovibrionales	Bdellovibrionaceae	OM27_clade	metagenome
SV_1321		Bdellovibrionia	Bdellovibrionales	Bdellovibrionaceae	OM27_clade	metagenome
SV_1536	Bdellovibrionota	Bdellovibrionia	Bacteriovoracales	Bacteriovoracaceae	Peredibacter	Peredibacter_starrii
SV_1711		Oligoflexia	0319-6G20	NA	NA	NA
SV_1775		Oligoflexia	0319-6G20	NA	NA	NA
SV_1866		Bdellovibrionia	Bdellovibrionales	Bdellovibrionaceae	OM27_clade	uncultured_bacterium
SV_1903		Bdellovibrionia	Bdellovibrionales	Bdellovibrionaceae	OM27_clade	uncultured_bacterium
SV_2293		Oligoflexia	0319-6G20	NA	NA	NA
**SV_2**		KD4-96	NA	NA	NA	NA
**SV_10**		KD4-96	NA	NA	NA	NA
**SV_60**		KD4-96	NA	NA	NA	uncultured_bacterium
**SV_79**		Gitt-GS-136	NA	NA	NA	uncultured_bacterium
**SV_104**	Chloroflexi	OLB14	NA	NA	NA	uncultured_bacterium
**SV_107**		Gitt-GS-136	NA	NA	NA	uncultured_bacterium
**SV_114**		Anaerolineae	Anaerolineales	Anaerolineaceae	uncultured	uncultured_prokaryote
**SV_120**		Gitt-GS-136	NA	NA	NA	NA
**SV_180**		Gitt-GS-136	NA	NA	NA	NA
SV_305		Anaerolineae	Ardenticatenales	uncultured	NA	uncultured_bacterium
**SV_65**		Cyanobacteriia	Cyanobacteriales	Phormidiaceae	Tychonema_CCAP_1459-11	NA
**SV_190**		Cyanobacteriia	Chloroplast	NA	NA	NA
**SV_222**		Cyanobacteriia	Cyanobacteriales	Coleofasciculaceae	NA	NA
**SV_376**		Cyanobacteriia	Cyanobacteriales	Coleofasciculaceae	Microcoleus_PCC-7113	Microcoleus_sp.
SV_469	Cyanobacteria	Cyanobacteriia	Cyanobacteriales	Coleofasciculaceae	Microcoleus_SAG_1449-1a	NA
SV_518		Cyanobacteriia	Oxyphotobacteria_I	Unknown_Family	Leptolyngbya_EcFYyyy-00	uncultured_bacterium
SV_800		Cyanobacteriia	Cyanobacteriales	Nostocaceae	NA	NA
SV_1132		Cyanobacteriia	Cyanobacteriales	Nostocaceae	Nostoc_PCC-7524	Nostoc_sp.

SV_1244		Cyanobacteriia	Cyanobacteriales	Phormidiaceae	uncultured	uncultured_bacterium
SV_1363		Cyanobacteriia	Cyanobacteriales	Nostocaceae	NA	NA
**SV_140**		uncultured	NA	NA	NA	uncultured_bacterium
**SV_300**		Desulfuromonadia	Geobacterales	Geobacteraceae	uncultured	uncultured_delta
**SV_356**		uncultured	NA	NA	NA	NA
SV_494		Desulfuromonadia	Geobacterales	Geobacteraceae	NA	NA
SV_575	Desulfobacterota	uncultured	NA	NA	NA	NA
SV_650		uncultured	NA	NA	NA	NA
SV_668		uncultured	NA	NA	NA	NA
SV_768		uncultured	NA	NA	NA	uncultured_bacterium
SV_832		Desulfuromonadia	Geobacterales	Geobacteraceae	NA	NA
SV_1057		Desulfuromonadia	Geobacterales	Geobacteraceae	NA	NA
SV_999		Lineage_IIa	NA	NA	NA	uncultured_bacterium
SV_1434		Lineage_IIa	NA	NA	NA	uncultured_bacterium
SV_1459		Lineage_IIb	NA	NA	NA	uncultured_soil
SV_1531		Elusimicrobia	Lineage_IV	NA	NA	uncultured_Termite
SV_1590	Elusimicrobiota	Lineage_IIb	NA	NA	NA	uncultured_bacterium
SV_1595		Lineage_IIa	NA	NA	NA	NA
SV_2014		Elusimicrobia	MVP-88	NA	NA	NA
SV_2146		Lineage_IIb	NA	NA	NA	uncultured_bacterium
SV_2185		Lineage_IIa	NA	NA	NA	NA
SV_2322		Elusimicrobia	Lineage_IV	NA	NA	uncultured_bacterium
SV_584		Bacilli	Bacillales	Bacillaceae	Bacillus	NA
SV_826		Bacilli	Alicyclobacillales	Alicyclobacillaceae	Tumebacillus	uncultured_bacterium
SV_977		Bacilli	Bacillales	Bacillaceae	Bacillus	NA
SV_980		Bacilli	Bacillales	Bacillaceae	Bacillus	Bacillus_luciferensis
SV_981	Firmicutes	Bacilli	Bacillales	Bacillaceae	Bacillus	NA
SV_1037		Bacilli	Bacillales	Bacillaceae	Bacillus	NA
SV_1167		Bacilli	Bacillales	Bacillaceae	Bacillus	NA
SV_1930		Clostridia	Clostridiales	Clostridiaceae	Clostridium_sensu_stricto_1	NA
SV_2278		Clostridia	Clostridiales	Clostridiaceae	Clostridium_sensu_stricto_1	NA
SV_2580		Bacilli	Alicyclobacillales	Alicyclobacillaceae	Tumebacillus	uncultured_bacterium
**SV_13**		Gemmatimonadetes	Gemmatimonadales	Gemmatimonadaceae	uncultured	uncultured_soil
**SV_25**		Gemmatimonadetes	Gemmatimonadales	Gemmatimonadaceae	uncultured	uncultured_bacterium
**SV_31**		Gemmatimonadetes	Gemmatimonadales	Gemmatimonadaceae	uncultured	uncultured_Gemmatimonadales
**SV_39**		AKAU4049	NA	NA	NA	uncultured_soil
**SV_46**	Gemmatimonadota	S0134_terrestrial_group	NA	NA	NA	uncultured_bacterium
**SV_47**		Gemmatimonadetes	Gemmatimonadales	Gemmatimonadaceae	uncultured	uncultured_bacterium
**SV_53**		Gemmatimonadetes	Gemmatimonadales	Gemmatimonadaceae	uncultured	metagenome
**SV_55**		Gemmatimonadetes	Gemmatimonadales	Gemmatimonadaceae	uncultured	NA
**SV_61**		Gemmatimonadetes	Gemmatimonadales	Gemmatimonadaceae	uncultured	uncultured_bacterium
SV_66		Gemmatimonadetes	Gemmatimonadales	Gemmatimonadaceae	uncultured	NA
**SV_35**		NA	NA	NA	NA	metagenome
**SV_52**		Latescibacteria	Latescibacterales	Latescibacteraceae	NA	uncultured_bacterium
**SV_73**		NA	NA	NA	NA	uncultured_soil
**SV_152**		NA	NA	NA	NA	metagenome
**SV_153**	Latescibacterota	NA	NA	NA	NA	uncultured_bacterium
**SV_171**		NA	NA	NA	NA	NA
**SV_263**		NA	NA	NA	NA	metagenome
**SV_280**		NA	NA	NA	NA	uncultured_bacterium
**SV_361**		NA	NA	NA	NA	metagenome
SV_452		NA	NA	NA	NA	NA
**SV_70**		Methylomirabilia	Rokubacteriales	WX65	NA	uncultured_bacterium
**SV_93**		Methylomirabilia	Rokubacteriales	NA	NA	uncultured_bacterium
**SV_98**		Methylomirabilia	Rokubacteriales	NA	NA	uncultured_bacterium
**SV_130**		Methylomirabilia	Rokubacteriales	NA	NA	uncultured_bacterium
**SV_200**	Methylomirabilota	Methylomirabilia	Rokubacteriales	NA	NA	uncultured_bacterium
**SV_211**		Methylomirabilia	Rokubacteriales	NA	NA	uncultured_bacterium
**SV_245**		Methylomirabilia	Rokubacteriales	NA	NA	uncultured_bacterium
**SV_287**		Methylomirabilia	Rokubacteriales	NA	NA	uncultured_bacterium
**SV_549**		Methylomirabilia	Rokubacteriales	NA	NA	uncultured_bacterium
SV_619		Methylomirabilia	Rokubacteriales	WX65	NA	uncultured_bacterium
**SV_86**		Polyangia	Polyangiales	BIrii41	NA	uncultured_bacterium
**SV_126**		Polyangia	Haliangiales	Haliangiaceae	Haliangium	uncultured_Myxococcales
**SV_164**		Polyangia	Polyangiales	Polyangiaceae	Pajaroellobacter	uncultured_bacterium
**SV_170**		Polyangia	Polyangiales	Polyangiaceae	Pajaroellobacter	metagenome
**SV_236**	Myxococcota	Polyangia	Haliangiales	Haliangiaceae	Haliangium	uncultured_bacterium
**SV_269**		Polyangia	Polyangiales	BIrii41	NA	uncultured_Sorangiineae
**SV_298**		Polyangia	Polyangiales	Phaselicystidaceae	Phaselicystis	uncultured_Polyangiaceae
**SV_366**		Polyangia	Haliangiales	Haliangiaceae	Haliangium	uncultured_bacterium
**SV_462**		Myxococcia	Myxococcales	Myxococcaceae	Archangium	Archangium_gephyra
SV_467		Polyangia	Haliangiales	Haliangiaceae	Haliangium	uncultured_Myxococcales

**SV_168**		NA	NA	NA	NA	uncultured_Desulfoglaeba
**SV_229**		NA	NA	NA	NA	uncultured_bacterium
**SV_274**		NA	NA	NA	NA	uncultured_bacterium
**SV_352**		NA	NA	NA	NA	uncultured_Desulfoglaeba
**SV_550**	NB1-j	NA	NA	NA	NA	uncultured_Desulfoglaeba
SV_600		NA	NA	NA	NA	uncultured_Desulfoglaeba
SV_678		NA	NA	NA	NA	uncultured_Desulfoglaeba
SV_717		NA	NA	NA	NA	uncultured_bacterium
SV_732		NA	NA	NA	NA	NA
SV_784		NA	NA	NA	NA	uncultured_Desulfoglaeba
**SV_44**		Nitrospiria	Nitrospirales	Nitrospiraceae	Nitrospira	uncultured_soil
**SV_82**		Nitrospiria	Nitrospirales	Nitrospiraceae	Nitrospira	uncultured_Green
**SV_195**		Nitrospiria	Nitrospirales	Nitrospiraceae	Nitrospira	uncultured_Green
**SV_478**		Nitrospiria	Nitrospirales	Nitrospiraceae	Nitrospira	uncultured_Green
SV_1171	Nitrospirota	Nitrospiria	Nitrospirales	Nitrospiraceae	Nitrospira	uncultured_soil
SV_1414		Nitrospiria	Nitrospirales	Nitrospiraceae	Nitrospira	NA
SV_1537		Nitrospiria	Nitrospirales	Nitrospiraceae	Nitrospira	uncultured_organism
SV_1735		Nitrospiria	Nitrospirales	Nitrospiraceae	Nitrospira	uncultured_soil
SV_2523		Nitrospiria	Nitrospirales	Nitrospiraceae	Nitrospira	uncultured_Green
SV_2689		Nitrospiria	Nitrospirales	Nitrospiraceae	Nitrospira	NA
SV_312		ABY1	Candidatus_Magas	NA	NA	uncultured_bacterium
SV_406		Parcubacteria	NA	NA	NA	uncultured_bacterium
SV_451		Parcubacteria	NA	NA	NA	uncultured_bacterium
SV_812		Parcubacteria	Candidatus_Nomur	NA	NA	uncultured_bacterium
SV_1007	Patescibacteria	Parcubacteria	NA	NA	NA	uncultured_bacterium
SV_1022		Parcubacteria	GWA2-38-13b	NA	NA	uncultured_bacterium
SV_1146		Parcubacteria	NA	NA	NA	uncultured_bacterium
SV_1301		Parcubacteria	Candidatus_Yanofs	NA	NA	uncultured_bacterium
SV_1358		Parcubacteria	Candidatus_Yanofs	NA	NA	NA
SV_1429		Parcubacteria	NA	NA	NA	uncultured_bacterium
**SV_37**		Phycisphaerae	Tepidisphaerales	WD2101_soil_group	NA	uncultured_soil
**SV_57**		Phycisphaerae	Tepidisphaerales	WD2101_soil_group	NA	uncultured_bacterium
**SV_91**		Planctomycetes	Pirellulales	Pirellulaceae	Pirellula	uncultured_soil
**SV_96**		OM190	NA	NA	NA	uncultured_bacterium
**SV_121**	Planctomycetota	Planctomycetes	Planctomycetales	Rubinisphaeraceae	SH-PL14	uncultured_Planctomycetaceae
**SV_129**		Phycisphaerae	Tepidisphaerales	WD2101_soil_group	NA	uncultured_bacterium
**SV_135**		Phycisphaerae	Tepidisphaerales	WD2101_soil_group	NA	NA
**SV_137**		Phycisphaerae	Tepidisphaerales	WD2101_soil_group	NA	uncultured_bacterium
**SV_160**		Phycisphaerae	Tepidisphaerales	WD2101_soil_group	NA	uncultured_soil
SV_162		Phycisphaerae	Tepidisphaerales	WD2101_soil_group	NA	uncultured_planctomycete
**SV_1**		Alphaproteobacteria	Azospirillales	uncultured	NA	uncultured_bacterium
**SV_3**		Gammaproteobacteria	Burkholderiales	Nitrosomonadaceae	MND1	NA
**SV_6**		Alphaproteobacteria	Dongiales	Dongiaceae	Dongia	NA
**SV_7**		Gammaproteobacteria	Burkholderiales	Nitrosomonadaceae	MND1	NA
**SV_11**	Proteobacteria	Alphaproteobacteria	Rhizobiales	Xanthobacteraceae	NA	NA
**SV_14**		Gammaproteobacteria	Burkholderiales	Nitrosomonadaceae	MND1	NA
**SV_15**		Gammaproteobacteria	Burkholderiales	SC-I-84	NA	uncultured_bacterium
**SV_16**		Gammaproteobacteria	Xanthomonadales	Xanthomonadaceae	Lysobacter	NA
**SV_21**		Gammaproteobacteria	Burkholderiales	Nitrosomonadaceae	Ellin6067	NA
SV_22		Gammaproteobacteria	CCD24	NA	NA	NA
**SV_17**		Verrucomicrobiae	Pedosphaerales	Pedosphaeraceae	uncultured	NA
**SV_18**		Verrucomicrobiae	Opitutales	Opitutaceae	Opitutus	NA
**SV_24**		Verrucomicrobiae	Pedosphaerales	Pedosphaeraceae	uncultured	metagenome
**SV_28**		Verrucomicrobiae	Pedosphaerales	Pedosphaeraceae	uncultured	metagenome
**SV_32**	Verrucomicrobiota	Verrucomicrobiae	Pedosphaerales	Pedosphaeraceae	NA	NA
**SV_40**		Verrucomicrobiae	Chthoniobacterales	Xiphinematobacteraceae	Candidatus_Xiphinematobac	NA
**SV_69**		Verrucomicrobiae	Pedosphaerales	Pedosphaeraceae	NA	uncultured_bacterium
**SV_113**		Verrucomicrobiae	Pedosphaerales	Pedosphaeraceae	NA	uncultured_bacterium
**SV_131**		Verrucomicrobiae	Pedosphaerales	Pedosphaeraceae	NA	NA
SV_149		Verrucomicrobiae	Pedosphaerales	Pedosphaeraceae	NA	uncultured_subdivision

Supplementary Table S2. Tukey-Kramer test for assessing difference of alpha diversity of animal-derived SVs between two sample groups. Difference of Shannon indexes of animal-derived SVs between two sample groups isolated from control and plant sites was investigated by Tukey-Kramer test. P adj: ajusted p-value. Less than 0.01 of p adj was considered significantly different and indicated by yellow color.

Sample category	Tested sample pairs		diff	lwr	upr	p adj
Control	Feb.	Apr.	0.197	-1.121	1.515	1.000
(uncultivated site)	Feb.	May	0.913	-0.405	2.231	0.348
	Feb.	Jun.	0.053	-1.265	1.371	1.000
	Feb.	Aug.	1.540	0.222	2.858	0.014
	Apr.	May	0.716	-0.601	2.034	0.654
	Apr.	Jun.	-0.144	-1.462	1.174	1.000
	Apr.	Aug.	1.343	0.025	2.661	0.044
	May	Jun.	-0.860	-2.178	0.458	0.423
	May	Aug.	0.627	-0.691	1.945	0.792
	Jun.	Aug.	1.487	0.169	2.805	0.019
Plant	Feb.	Apr.	0.511	-0.807	1.829	0.922
(cultivated site)	Feb.	May	1.097	-0.220	2.415	0.156
	Feb.	Jun.	1.306	-0.012	2.624	0.053
	Feb.	Aug.	1.991	0.673	3.309	0.001
	Apr.	May	0.586	-0.731	1.904	0.845
	Apr.	Jun.	0.795	-0.522	2.113	0.525
	Apr.	Aug.	1.480	0.163	2.798	0.020
	May	Jun.	0.209	-1.109	1.527	1.000
	May	Aug.	0.894	-0.424	2.212	0.374
	Jun.	Aug.	0.685	-0.633	2.003	0.704

Supplementary Table S3. Difference of Bray-Curtis distances of SVs between two consecutive sample groups were investigated using pairwiseAdnis2 program. Less than 0.05 of *P* adjusted was considered significantly different and indicated by yellow color. The SVs derived from the sample category were tested (see Fig. 3).

**Sample categry**	**Tested sample group1**	**Tested sample group2**	***F***	R^2	***P* adjusted**
Prokaryote	Feb.	Apr.	2.9928	0.2303	0.020
	Apr.	May	1.4499	0.1266	0.970
	May	Jun.	1.3045	0.1154	1.000
	Jun.	Aug.	2.4488	0.1967	0.100
Eukaryote	Feb.	Apr.	4.8310	0.3257	0.010
	Apr.	May	3.1482	0.2394	0.040
	May	Jun.	1.9982	0.1665	0.170
	Jun.	Aug.	5.9301	0.3723	0.030
Fungi	Feb.	Apr.	7.0361	0.4130	0.070
	Apr.	May	1.3577	0.1195	1.000
	May	Jun.	2.0806	0.1722	0.170
	Jun.	Aug.	6.8004	0.4048	0.040
Protists	Feb.	Apr.	5.1543	0.3401	0.020
	Apr.	May	4.2487	0.2982	0.010
	May	Jun.	1.8141	0.1536	0.300
	Jun.	Aug.	6.2686	0.3853	0.030
Animal	Feb.	Apr.	1.9768	0.1651	1.000
	Apr.	May	3.1381	0.2389	0.030
	May	Jun.	1.3773	0.1211	1.000
	Jun.	Aug.	3.7510	0.2728	0.080
Nematode	Feb.	Apr.	3.6214	0.2659	0.030
	Apr.	May	4.1043	0.2910	0.010
	May	Jun.	3.4914	0.2588	0.020
	Jun.	Aug.	6.9913	0.4115	0.020

Supplementary Table S4. Abundant SVs in each eukaryotic phylum, their taxa based on the SILVA database, and the top hits identified by BLASTN search.Abundant SVs in each eukaryotic phylum and their SILVA-based taxa are shown. The top hits by BLASTN search (May in 2024), their e-values, max scores, and accession numbers are also indicated. NA: not assigned. Accession numbers with "etc." indicate multiple hits with the same scores (these hit species are separated by forward slash). The top 10 SVs in abundance are indicated in the phylum containing more than 10 SVs, and several abundant SVs in the phylum containing less than 10 SVs. Abundant SVs in unassigned phyla are not shown. SVs in bold were used for Figs. S6, S8, and S9.

Fungi

ID	Phylum	Class	Order	Family	Genus	Species	The top hits by BLASTN search	e-value	Max score	Accession No.
**SV_91**		NA	NA	NA	uncultured	uncultured_fungus	Aphelidium parallelum	1e-174	627	LC652435.1
**SV_121**		NA	NA	NA	uncultured	uncultured_fungus	Aphelidium parallelum	1e-174	658	LC652435.1
SV_921		NA	NA	NA	Paraphelidium	NA	Urophlyctis sp.	2e-171	616	HQ888719.1 etc.
SV_986		NA	NA	NA	uncultured	uncultured_eukaryote	Amoeboaphelidium occidentale	0	710	JX967274.1
SV_1150	Aphelidea	NA	NA	NA	uncultured	uncultured_eukaryote	Aphelidium insulamus	0	699	MW186929.1
SV_1379		NA	NA	NA	uncultured	uncultured_fungus	Aphelidium insulamus	0	688	MW186929.1
SV_1471		NA	NA	NA	Paraphelidium	uncultured_eukaryote	Paraphelidium tribonematis	0	761	MK631785.1
SV_1589		NA	NA	NA	NA	NA	Amoeboaphelidium occidentale	0	737	JX967274.1
SV_1764		NA	NA	NA	Paraphelidium	uncultured_eukaryote	Paraphelidium tribonematis	0	728	MK631785.1
SV_1809		NA	NA	NA	uncultured	uncultured_fungus	Aphelidium parallelum	0	667	LC652435.1
**SV_8**		Sordariomycetes	Sordariales	Chaetomiaceae	Chaetomium	NA	Chaetomium globosum/Humicola sp.	0	763	MN602645.1 etc.
**SV_12**		Sordariomycetes	Hypocreales	Nectriaceae	Fusarium	NA	Fusarium sp.	0	758	MN602645.1 etc.
**SV_16**		Sordariomycetes	Sordariales	Chaetomiaceae	Chaetomium	NA	Chaetomium globosum/Humicola sp.	0	758	MN602645.1 etc.
**SV_21**		Dothideomycetes	Capnodiales	Cladosporiaceae	Cladosporium	NA	Cladosporium sp.	0	767	MT644477.1 etc.
**SV_27**		Sordariomycetes	Sordariales	Chaetomiaceae	Chaetomium	NA	Chaetomium sp./Madurella sp./Corynascus verrucosus/Thermothelomyces sp.	0	763	MT649557.1 etc.
**SV_31**	Ascomycota	Dothideomycetes	Pleosporales	Pleosporaceae	NA	NA	Bipolaris sp./Curvularia sp./Paradendryphiella arenariae	0	767	MT649586.1 etc.
**SV_43**		Pezizomycetes	Pezizales	Rhizinaceae	Phymatotrichopsis	uncultured_eukaryote	Ascobolus sp.	0	771	MN995515.1 etc.
**SV_44**		Sordariomycetes	Hypocreales	NA	NA	NA	Fusarium sp.	0	763	MT649540.1 etc.
SV_46		Dothideomycetes	Pleosporales	NA	NA	NA	Parastagonospora sp./Ophiosphaerella sp./Phaeosphaeriopsis sp./Setomelanomma holmii	0	767	CP039677.1 etc.
SV_48		Dothideomycetes	Pleosporales	NA	NA	NA	Phoma sp./Boeremia sp./Ascochyta sp./Microsphaeropsis olivacea	0	767	LR745531.1 etc.
**SV_17**		Agaricomycetes	NA	NA	NA	NA	Trechispora sp.	0	713	EU909231.1 etc.
**SV_37**		Agaricomycetes	Agaricales	NA	NA	NA	Lyophyllum sp./Crassisporium funariophilum/Nidula niveotomentosa/Psathyrella gracilis/Myochromella boudieri/Hebeloma angustilamellatum/Agaricus sp.	0	769	KU058570.1 etc.
**SV_149**		Agaricomycetes	Agaricales	NA	NA	NA	Collybia sp./Lyophyllum sp.	0	752	MT644912.1 etc.
**SV_165**	Basidiomycota	Agaricomycetes	Auriculariales	NA	NA	NA	Alloexidiopsis calcea	0	739	AY293130.1
**SV_167**		Agaricomycetes	Corticiales	Corticiaceae	Sistotrema	uncultured_fungus	Minimedusa polyspora	0	769	KC176294.1
SV_184		Agaricomycetes	Agaricales	NA	NA	NA	Hohenbuehelia sp.	0	769	JQ926736.2 etc.
SV_204		Agaricomycetes	Agaricales	NA	NA	NA	Stephanospora caroticolor	0	730	AF518591.1
SV_249		Agaricomycetes	NA	NA	NA	NA	Bjerkandera sp./Ganoderma sp./Phanerodontia chrysosporium/Lentinus crinitus/Hyphodermella sp. etc.	0	752	MT644869.1 etc.
SV_265		Agaricomycetes	uncultured	NA	NA	uncultured_Boletaceae	Serendipita vermifera	0	769	EU625993.1 etc.
SV_358		Agaricomycetes	Agaricales	NA	NA	NA	Eimeria lateralis	0	758	OP407718.1 etc.
**SV_259**		Blastocladiomycetes	Blastocladiales	Physodermataceae	Paraphysoderma	Paraphysoderma_sedebokerense	Paraphysoderma sedebokerense	0	747	MN203631.1 etc.
**SV_663**		Blastocladiomycetes	Blastocladiales	Physodermataceae	Paraphysoderma	Paraphysoderma_sedebokerense	Paraphysoderma sedebokerense	0	769	MN203631.1 etc.
SV_715		Blastocladiomycetes	Blastocladiales	Physodermataceae	NA	uncultured_fungus	Paraphysoderma sedebokerense	0	758	MN203631.1 etc.
SV_1820		Blastocladiomycetes	Blastocladiales	Physodermataceae	Paraphysoderma	Paraphysoderma_sedebokerense	Paraphysoderma sedebokerense	0	747	MN203631.1 etc.
SV_1927	Blastocladiomycota	Blastocladiomycetes	Blastocladiales	Physodermataceae	NA	NA	Rozella sp.	0	730	OQ702856.1
SV_1930		Blastocladiomycetes	Blastocladiales	Physodermataceae	Paraphysoderma	Paraphysoderma_sedebokerense	Paraphysoderma sedebokerense	0	706	MN203631.1 etc.
SV_2246		Blastocladiomycetes	Blastocladiales	Physodermataceae	NA	uncultured_fungus	Paraphysoderma sedebokerense	0	726	MN203631.1 etc.
SV_2429		Blastocladiomycetes	Blastocladiales	Physodermataceae	Paraphysoderma	Paraphysoderma_sedebokerense	Paraphysoderma sedebokerense	0	747	MN203631.1 etc.
SV_2618		Blastocladiomycetes	Blastocladiales	Physodermataceae	NA	NA	Paraphysoderma sedebokerense	0	715	MN203631.1 etc.
SV_3455		Blastocladiomycetes	Blastocladiales	Physodermataceae	NA	NA	Paraphysoderma sedebokerense	0	712	MN203631.1 etc.
**SV_13**		Chytridiomycetes	NA(Chytridiales)	NA	NA	NA	Chytridium polysiphoniae	0	695	AY032608.1
**SV_47**		Chytridiomycetes	Spizellomycetales	Olpidiaceae	Olpidium	NA	Olpidium brassicae	0	763	NG_017176.1
**SV_65**		Chytridiomycetes	Rhizophydiales	Rhizophydiaceae	Rhizophydium	uncultured_Rhizophydiales	Rhizophydium patellarium/Betamyces sp.	0	725	KF160860.1 etc.
**SV_94**		Chytridiomycetes	Rhizophydiales	uncultured	NA	uncultured_Chytridiomycota	Rhizophydium sp. UGA-F16	0	752	AH009034.2
**SV_102**		Chytridiomycetes	NA	NA	NA	NA	Catenomyces persicinus	0	763	AY635830.1
SV_134	Chytridiomycota	Chytridiomycetes	Rhizophydiales	uncultured	NA	uncultured_Rhizophydiales	Paranamyces uniporus	0	656	MT731025.1 etc.
SV_138		Chytridiomycetes	Rhizophydiales	uncultured	NA	Rhizophydium_sp.	Aquamyces sp./Rhizophydium sp.	0	747	MT730892.1 etc.
SV_139		Chytridiomycetes	NA	NA	NA	NA	Fimicolochytrium sp.	0	763	NG_062391.1 etc.
SV_141		Chytridiomycetes	Rhizophydiales	uncultured	NA	uncultured_fungus	Rhizophydium sp.	0	769	MT730997.1 etc.
SV_150		Chytridiomycetes	Chytridiales	Incertae_Sedis	NA	Pseudorhizidium_endosporangiatum	Delfinachytrium mesopotamicum	0	763	MT730617.1
**SV_161**		Incertae_Sedis	Incertae_Sedis	Incertae_Sedis	Rozella	uncultured_fungus	Rozella sp.	0	667	OQ702932.1 etc.
**SV_327**		NA	NA	NA	NA	uncultured_fungus	Vinositunica sp./Bullera formosensis	6e-157	568	LC431094.1 etc.
**SV_421**		Incertae_Sedis	Incertae_Sedis	Incertae_Sedis	Rozella	uncultured_fungus	Rozella sp.	0	734	KX354831.1
SV_683		Incertae_Sedis	Incertae_Sedis	Incertae_Sedis	Rozella	uncultured_fungus	Rozella sp.	0	706	OQ702917.1
SV_913		Incertae_Sedis	Incertae_Sedis	Incertae_Sedis	Paramicrosporidium	uncultured_eukaryote	Paramicrosporidium sp.	1e-169	619	MW898450.1
SV_1127	Cryptomycota	Incertae_Sedis	Incertae_Sedis	Incertae_Sedis	Rozella	uncultured_fungus	Rozella sp.	0	752	OQ702856.1
SV_1265		NA	NA	NA	NA	uncultured_fungus	Vinositunica sp.	1e-158	573	LC431094.1 etc.
SV_1376		Incertae_Sedis	Incertae_Sedis	Incertae_Sedis	Rozella	uncultured_fungus	Rozella sp.	0	706	KX354831.1
SV_1411		NA	NA	NA	NA	NA	Paraphysoderma sedebokerense/Arkaya serpentina	6e-162	584	MN203631.1 etc.
SV_1465		Incertae_Sedis	Incertae_Sedis	Incertae_Sedis	Rozella	uncultured_fungus	Rozella sp.	0	695	OQ702932.1 etc.
**SV_147**		NA	NA	NA	NA	uncultured_eukaryote	Podoscypha sp.	5e-148	538	KY464924.1 etc.
**SV_247**		NA	NA	NA	NA	metagenome	Morellospora saccamoebae	1e-178	640	MN821072.1
SV_827		NA	NA	NA	NA	NA	Morellospora saccamoebae	0	662	MN821072.1
SV_854		NA	NA	NA	NA	NA	Morellospora saccamoebae	1e-149	544	MN821072.1
SV_1420	LKM15	NA	NA	NA	NA	uncultured_eukaryote	Chytriomyces sp. JEL341	8e-161	580	DQ536482.1
SV_1491		NA	NA	NA	NA	NA	Morellospora saccamoebae	5e-158	571	MN821072.1
SV_1770		NA	NA	NA	NA	NA	Morellospora saccamoebae	5e-163	588	MN821072.1
SV_1815		NA	NA	NA	NA	NA	Morellospora saccamoebae	0	669	MN821072.1
SV_2039		NA	NA	NA	NA	metagenome	Morellospora saccamoebae	0	737	MN821072.1
SV_2115		NA	NA	NA	NA	NA	Morellospora saccamoebae	0	710	MN821072.1
**SV_9**		Incertae_Sedis	Mortierellales	Mortierellaceae	Mortierella	NA	Mortierella sp./Linnemannia elongata/Entomortierella sp./Podila verticillata	0	769	MH047197.1 etc.
**SV_59**		Glomeromycetes	Glomerales	Glomeraceae	Funneliformis	Funneliformis_mosseae	Glomus sp./Funneliformis sp.	0	771	FR750227.1 etc.
**SV_90**		Glomeromycetes	Diversisporales	Gigasporaceae	NA	NA	Cetraspora gilmorei	0	763	FR773143.1 etc.
**SV_124**		Incertae_Sedis	Mortierellales	Mortierellaceae	Mortierella	NA	Actinomortierella wolfii	0	758	KU141329.1 etc.
**SV_151**	Mucoromycota	Incertae_Sedis	Mortierellales	Mortierellaceae	Mortierella	Mortierella_sp.	Mortierella sp. MS-6	0	776	AY635828.1
SV_219		Glomeromycetes	Glomerales	uncultured	NA	NA	Glomus mycorrhizal symbiont of Marchantia foliacea	0	706	AJ699068.1 etc.
SV_241		Glomeromycetes	Diversisporales	NA	NA	Glomeromycotina_sp.	Entrophospora sp. AS-2013	0	752	JF414174.1
SV_245		Incertae_Sedis	Mortierellales	Mortierellaceae	Mortierella	Mortierella_sp.	Mortierella sp. MS-6	0	765	AY635828.1
SV_266		Glomeromycetes	uncultured	NA	NA	uncultured_fungus	Glomus macrocarpum	0	737	FR750376.1 etc.
SV_273		Glomeromycetes	Diversisporales	Acaulosporaceae	Acaulospora	Glomeromycotina_sp.	Acaulospora sp.	0	706	NG_062371.1 etc.
SV_873		Neocallimastigomycetes	Neocallimastigales	Neocallimastigaceae	uncultured	uncultured_fungus	Monoblepharis sp.	0	693	KJ668077.1 etc.
SV_1105	Neocallimastigomycota	Neocallimastigomycetes	Neocallimastigales	Neocallimastigaceae	uncultured	uncultured_fungus	Olpidium brassicae	0	697	NG_017176.1

SV_2743		Neocallimastigomycetes	Neocallimastigales	Neocallimastigaceae	uncultured	uncultured_fungus	Monoblepharis sp.	0	673	KJ668077.1 etc.
SV_3667		NA	NA	Nucleariidae	Nuclearia	NA	Entrophospora etunicata	1e-174	627	Z14008.1
SV_3923		NA	NA	Nucleariidae	Nuclearia	metagenome	Rozella sp.	3e-165	595	OQ702932.1 etc.
SV_3924		NA	NA	Nucleariidae	Nuclearia	metagenome	Rozella sp.	1e-173	623	OQ702932.1 etc.
SV_3986		NA	NA	Nucleariidae	Nuclearia	NA	Harpochytrium sp.	8e-161	580	KJ668051.1 etc.
SV_3989	Nucleariidae_and_Fonticula_group	NA	NA	Nucleariidae	Nuclearia	NA	Acaulospora sp.	6e-172	617	AJ306439.1 etc.
SV_4353		NA	NA	Nucleariidae	Nuclearia	metagenome	Harpochytrium sp.	1e-163	590	KJ668051.1 etc.
SV_4893		NA	NA	Nucleariidae	Nuclearia	NA	Pompholyxophrys sp.	2e-151	549	MK547175.1 etc.
SV_5118		NA	NA	Nucleariidae	Nuclearia	NA	Rozella sp.	7e-162	584	OQ702932.1 etc.
SV_5123		NA	NA	Nucleariidae	Nuclearia	NA	Basidioascus sp. CBS 12316	2e-166	599	LT548272.1
SV_5205		NA	NA	Nucleariidae	Nuclearia	NA	Pompholyxophrys punicea	2e-142	520	MK547175.1
**SV_407**		Incertae_Sedis	Kickxellales	Kickxellaceae	Ramicandelaber	Ramicandelaber_brevisporus	Ramicandelaber sp. KYK00187	0	754	AB287991.1
SV_729		Entomophthoromycetes	Entomophthorales	Ancylistaceae	Conidiobolus	Conidiobolus_brefeldianus	Conidiobolus brefeldianus	0	787	NG_065500.1
SV_1733		Entomophthoromycetes	Entomophthorales	Ancylistaceae	Conidiobolus	Conidiobolus_coronatus	Conidiobolus coronatus	0	793	FJ895303.1
SV_2038		Incertae_Sedis	Zoopagales	NA	NA	metagenome	Stylopage hadra	0	761	MN252062.1
SV_2213	Zoopagomycota	Entomophthoromycetes	Entomophthorales	uncultured	NA	uncultured_fungus	Neokarlingia chitinophila	8e-171	614	HQ901766.1
SV_2454		Entomophthoromycetes	Entomophthorales	Ancylistaceae	Conidiobolus	NA	Conidiobolus sp. RSL-II	0	782	FJ895304.1
SV_2482		Incertae_Sedis	Zoopagales	NA	NA	metagenome	Stylopage hadra	0	671	MN252062.1
SV_2623		Incertae_Sedis	Kickxellales	Kickxellaceae	Ramicandelaber	Ramicandelaber_brevisporus	Ramicandelaber taiwanensis	0	756	JQ004926.1 etc.
SV_3161		Incertae_Sedis	Harpellales	NA	NA	NA	Paraphysoderma sedebokerense	1e-179	643	MN203631.1 etc.
SV_3315		Basidiobolomycetes	Basidiobolales	Basidiobolaceae	Basidiobolus	NA	Basidiobolus sp.	0	771	MW130090.1 etc.

Protists

ID	Phylum	Class	Order	Family	Genus	Species	The top hits by BLASTN search	e-value	Max score	Accession No.
SV_3914	Ancyromonadida	NA	NA	NA	NA	Ancyromonas_sigmoides	Ancyromonas sp.	0	763	MW872726.1 etc.
SV_4022		NA	NA	NA	NA	Nutomonas_longa	Nutomonas longa	0	800	MW872732.1 etc.
**SV_36**		Conoidasida	Gregarinasina	Archigregarinorida	Selenidium	uncultured_alveolate	Eimeria tropidura	3e-160	579	AF324217.1
SV_818		Conoidasida	Gregarinasina	Eugregarinorida	Elev-18S-1089	uncultured_Eimeriidae	Coronoepimeritus otoensis/Quadruspinospora mexicana	0	743	MK181532.1 etc.
SV_1364		Conoidasida	Gregarinasina	Eugregarinorida	Gregarina	NA	Gregarina sp.	2e-166	599	LR814086.1 etc.
SV_1664		Conoidasida	Gregarinasina	Eugregarinorida	uncultured	uncultured_alveolate	Coronoepimeritus otoensis/Quadruspinospora mexicana	0	721	MK181532.1 etc.
SV_1835		Conoidasida	Gregarinasina	Eugregarinorida	NA	NA	Coronoepimeritus otoensis/Quadruspinospora mexicana	4e-169	608	MK181532.1 etc.
SV_2481	Apicomplexa	Conoidasida	Gregarinasina	Eugregarinorida	Elev-18S-1089	NA	Coronoepimeritus otoensis/Quadruspinospora mexicana	0	710	MK181532.1 etc.
SV_2637		Conoidasida	Gregarinasina	Eugregarinorida	NA	NA	Coronoepimeritus otoensis/Quadruspinospora mexicana	0	682	MK181532.1 etc.
SV_2716		Conoidasida	Gregarinasina	Eugregarinorida	Gregarina	NA	Gregarina sp.	8e-161	580	LR814105.1 etc.
SV_2833		Conoidasida	Gregarinasina	Eugregarinorida	Elev-18S-1089	uncultured_Eimeriidae	Coronoepimeritus otoensis/Quadruspinospora mexicana	0	710	MK181532.1 etc.
SV_3157		Conoidasida	Gregarinasina	Eugregarinorida	NA	NA	Coronoepimeritus otoensis/Quadruspinospora mexicana	2e-172	619	MK181532.1 etc.
SV_680		NA	NA	NA	Apusomonas	Apusomonas_proboscidea	Apusomonas proboscidea	0	723	OM966642.1
SV_1336		NA	NA	NA	Apusomonas	Apusomonas_proboscidea	Apusomonas proboscidea	0	758	DQ207568.1
SV_1394		NA	NA	NA	Apusomonas	Apusomonas_proboscidea	Apusomonas proboscidea	0	747	DQ207568.1
SV_1570		NA	NA	NA	Apusomonas	NA	Apusomonas proboscidea	0	713	DQ207568.1
SV_1876		NA	NA	NA	Apusomonas	Apusomonas_proboscidea	Apusomonas proboscidea	0	763	DQ207567.1
SV_1968	Apusomonadidae	NA	NA	NA	Apusomonas	Apusomonas_proboscidea	Apusomonas proboscidea	0	774	DQ207567.1
SV_2236		NA	NA	NA	Apusomonas	NA	Podomonas kaiyoae/Apusomonas proboscidea	0	652	DQ207568.1 etc.
SV_2608		NA	NA	NA	Apusomonas	NA	Apusomonas australiensis	0	706	OM966637.1
SV_2625		NA	NA	NA	Apusomonas	Apusomonas_proboscidea	Apusomonas proboscidea	0	752	DQ207568.1
SV_2627		NA	NA	NA	Apusomonas	Apusomonas_proboscidea	Apusomonas proboscidea	0	723	OM966642.1
**SV_307**		NA	NA	NA	Bicosoeca	metagenome	Trieres chinensis	1e-153	556	MG835771.1
**SV_614**		NA	NA	NA	Bicosoeca	uncultured_eukaryote	Bicosoeca petiolata	0	676	AY520444.1
**SV_643**		NA	NA	NA	NA	NA	Adriamonas peritocrescens	0	654	AF243501.1
**SV_750**		uncultured	NA	NA	NA	uncultured_microeukaryote	Pseudobodo sp.	8e-156	564	LC683683.1 etc.
SV_824	Bicosoecida	NA	NA	NA	NA	NA	Nanos amicus	2e-136	499	FJ971853.1
SV_1094		NA	NA	NA	Bicosoeca	metagenome	Bicosoeca kenaiensis	2e-162	586	KM816648.1
SV_1169		NA	NA	NA	NA	NA	Adriamonas peritocrescens	0	654	AF243501.1
SV_1210		K4	NA	NA	NA	uncultured_freshwater	Adriamonas peritocrescens	0	654	AF243501.1
SV_1309		NA	NA	NA	NA	NA	Nanos amicus	2e-142	520	FJ971853.1
SV_1321		NA	NA	NA	Bicosoeca	metagenome	Bicosoeca kenaiensis	2e-157	569	KM816648.1
**SV_168**		NA	NA	Heterophryidae	NA	metagenome	Triangulopteris lacunata	0	760	OL739463.1
**SV_239**		NA	NA	Heterophryidae	NA	metagenome	Khitsovia mutabilis	0	776	OP101622.1
SV_535		NA	NA	NA	NA	NA	Chlamydaster sterni	0	732	AF534709.1
SV_539		NA	NA	NA	NA	NA	Chlamydaster sterni	0	728	AF534709.1
SV_555		NA	NA	Heterophryidae	NA	metagenome	Triangulopteris lacunata	0	752	OL739463.1
SV_585	Centrohelida	NA	NA	NA	NA	NA	Chlamydaster sterni	0	719	AF534709.1
SV_627		NA	NA	NA	NA	NA	Chlamydaster sterni	0	725	AF534709.1
SV_726		NA	NA	NA	NA	NA	Chlamydaster sterni	0	713	AF534709.1
SV_761		NA	NA	Heterophryidae	NA	metagenome	Khitsovia mutabilis	0	765	OP101622.1
SV_1104		NA	NA	NA	NA	NA	Chlamydaster sterni	0	723	AF534709.1
**SV_20**		Thecofilosea	NA	NA	NA	NA	Rhogostoma bowseri	0	767	MN860289.1 etc.
**SV_24**		Vampyrellidae	NA	NA	NA	NA	Theratromyxa weberi/Arachnula impatiens	0	713	GQ377666.1 etc.
**SV_38**		Vampyrellidae	NA	NA	NA	NA	Vampyrella closterii	0	701	OQ591878.1
**SV_41**		Vampyrellidae	NA	NA	NA	Arachnula_impatiens	Theratromyxa weberi/Arachnula impatiens	0	725	GQ377666.1 etc.
**SV_52**		Vampyrellidae	NA	NA	NA	Platyreta_germanica	Platyreta germanica	0	760	AY941201.1
**SV_60**	Cercozoa	Cercomonadidae	NA	NA	Cercomonas	uncultured_eukaryote	Eocercomonas exploratorii	0	717	HM536154.1
SV_62		Glissomonadida	NA	NA	Heteromita	NA	Neoheteromita sp./Heteromita sp.	0	763	OR731273.1 etc.
SV_66		Vampyrellidae	NA	NA	Leptophrys	Leptophrys_vorax	Leptophrys vorax	0	710	HE609038.1 etc.
SV_67		Imbricatea	NA	Euglyphida	Euglypha	Euglypha_rotunda	Euglypha rotunda	0	761	AJ418784.1
SV_68		Vampyrellidae	NA	NA	NA	Arachnula_impatiens	Theratromyxa weberi/Arachnula impatiens	0	708	GQ377666.1 etc.
**SV_111**		Intramacronucleata	Conthreep	Colpodea	Colpoda	metagenome	Kalometopia duplicata	0	697	KJ873050.1
**SV_116**		Intramacronucleata	Conthreep	Nassophorea	Obertrumia	NA	Obertrumia georgiana	0	721	X65149.1
**SV_159**		Intramacronucleata	Conthreep	Nassophorea	Obertrumia	NA	Obertrumia georgiana	0	726	X65149.1
**SV_187**		Intramacronucleata	Conthreep	Colpodea	Colpoda	uncultured_eukaryote	Colpoda sp.	0	774	MK801290.1 etc.
**SV_189**		Intramacronucleata	Conthreep	Colpodea	NA	NA	Colpoda sp.	0	774	KJ607917.1 etc.
SV_217	Ciliophora	Intramacronucleata	Conthreep	Colpodea	Colpoda	metagenome	Kalometopia duplicata	0	765	KJ873050.1
SV_220		Intramacronucleata	Conthreep	Oligohymenophorea	Frontonia	NA	Frontonia tchibisovae	0	675	DQ885987.1 etc.
SV_269		Intramacronucleata	Conthreep	Colpodea	NA	NA	Pseudoplatyophrya nana	0	773	AF060452.1
SV_315		Intramacronucleata	Conthreep	Colpodea	Platyophrya	NA	Platyophrya bromelicola	0	730	EU039905.1
SV_317		Intramacronucleata	Conthreep	Colpodea	Platyophrya	metagenome	Platyophrya bromelicola	0	736	EU039905.1
**SV_3**		Bacillariophyceae	NA	NA	Hantzschia	Hantzschia_amphioxys	Hantzschia amphioxys	0	773	MN696729.1 etc.
**SV_18**		Bacillariophyceae	NA	NA	NA	NA	Craticula subminuscula/Stauroneis acuta	0	721	KT072989.1 etc.
**SV_30**		Bacillariophyceae	NA	NA	NA	NA	Hantzschia amphioxys	0	761	MN696729.1 etc.
**SV_103**		Bacillariophyceae	NA	NA	Amphora	Amphora_montana	Halamphora montana/Amphora montana	0	767	KX120666.1 etc.
**SV_106**		Bacillariophyceae	NA	NA	Nitzschia	NA	Nitzschia palea	0	773	PP158997.1 etc.
**SV_133**	Diatomea	Bacillariophyceae	NA	NA	NA	Fistulifera_saprophila	Fistulifera sp.	0	774	MH997844.1 etc.
SV_213		Bacillariophyceae	NA	NA	Fistulifera	Mayamaea_atomus	Mayamaea permitis/Fistulifera pelliculosa	0	754	AM501969.1 etc.
SV_292		Bacillariophyceae	NA	NA	Fistulifera	Mayamaea_atomus	Mayamaea permitis/Fistulifera pelliculosa	0	754	AM501969.1 etc.
SV_296		Bacillariophyceae	NA	NA	Fistulifera	Mayamaea_atomus	Mayamaea permitis/Fistulifera pelliculosa	0	749	AM501969.1 etc.
SV_329		Bacillariophyceae	NA	NA	NA	NA	Craticula subminuscula/Stauroneis acuta	0	715	KT072989.1 etc.
SV_553		Dinophyceae	NA	NA	NA	NA	Aduncodinium glandula	0	767	LK934662.8

SV_1442	Dinoflagellata	NA	NA	NA	NA	NA	Sourniaea diacantha/Gonyaulax whaseongensis	5e-118	438	MT039425.1 etc.
SV_1667		Dinophyceae	Gymnodiniphycida	Suessiaceae	Biecheleria	Woloszynskia_pascheri	Borghiella sp./Woloszynskia pascheri	0	761	MN604303.1 etc.
SV_2030		Dinophyceae	NA	NA	NA	NA	Esoptrodinium sp.	0	734	JQ439944.1 etc.
SV_2120		Dinophyceae	Gymnodiniphycida	Tovelliaceae	Esoptrodinium	Esoptrodinium_sp.	Esoptrodinium sp.	0	734	JQ439944.1 etc.
SV_2255		Dinophyceae	NA	NA	NA	NA	Pfiesteria piscicida	0	773	DQ991382.1 etc
SV_2330		Dinophyceae	NA	NA	NA	NA	Pfiesteria sp. B112456	0	773	AF218805.2
SV_2359		Dinophyceae	NA	NA	NA	NA	Aduncusdinium glandulum	0	734	LK934662.8
SV_2680		Dinophyceae	NA	NA	NA	NA	Aduncusdinium glandulum	0	660	LK934662.8
SV_2782		Dinophyceae	NA	NA	NA	NA	Barrufeta sp./Aduncodinium sp./Stoeckeria sp./Gymnodinium sp.	0	734	MH732694.1 etc.
SV_4257	Gracilipodida	NA	NA	NA	Telaepolella	Telaepolella_tubasferens	Milligania stylosa	2e-137	503	HM640763.1 etc.
SV_4472		NA	NA	NA	Telaepolella	Telaepolella_sp.	Nudifila producta	7e-132	484	HQ121434.1
SV_4481		NA	NA	NA	Telaepolella	Telaepolella_sp.	Pterosperma sp. CCMP1384	1e-123	457	KF899835.1
SV_4658		NA	NA	NA	Telaepolella	Telaepolella_sp.	Pennarella elegantia	7e-132	484	PP109353.1
SV_4663		NA	NA	NA	Telaepolella	Telaepolella_sp.	Micromonas sp.	4e-129	475	MT117943.1 etc.
SV_5316		NA	NA	NA	Telaepolella	Telaepolella_sp.	Basidiobolus sp.	3e-111	416	MW130090.1 etc.
**SV_97**	Hyphochytriomycetes	NA	Hyphochytriales	NA	NA	uncultured_eukaryote	Rhizidiomyces apophysatus	0	760	AF163295.1
**SV_117**		NA	Hyphochytriales	NA	Hyphochytrium	Hyphochytrium_catenoides	Hyphochytrium catenoides	0	776	X80344.1
SV_429		NA	Hyphochytriales	NA	NA	uncultured_eukaryote	Rhizidiomyces apophysatus	0	737	AF163295.1
SV_3656		NA	Hyphochytriales	NA	NA	NA	Rhizidiomyces apophysatus	1e-159	577	AF163295.1
SV_4521		NA	Hyphochytriales	NA	NA	NA	Rhizidiomyces apophysatus	3e-160	579	AF163295.1
SV_4719		NA	Hyphochytriales	NA	Hyphochytrium	Hyphochytrium_catenoides	Hyphochytrium catenoides	1e-164	593	AF163294.1
SV_5232		NA	Hyphochytriales	NA	Hyphochytrium	Hyphochytrium_catenoides	Hyphochytrium catenoides	1e-149	544	X80344.1
SV_803	Klebsormidiophyceae	NA	NA	NA	NA	NA	Klebsormidium sp.	0	778	MT425960.1 etc.
SV_201	MAST-12	MAST-12C	NA	NA	NA	uncultured_Eimeriidae	Oblongichytrium sp./Pseudophyllomitus vesiculosus/Mastreximonas tlaamin	5e-148	538	KY980042.1 etc.
SV_409		NA	NA	NA	NA	uncultured_stramenopile	Thalassomyxa sp. MVa1x	1e-109	411	KC779515.1
SV_742		MAST-12C	NA	NA	NA	NA	Oblongichytrium sp./Pseudophyllomitus vesiculosus/Mastreximonas tlaamin	2e-146	532	KY980042.1 etc.
SV_841		MAST-12C	NA	NA	NA	uncultured_Eimeriidae	Oblongichytrium sp.	1e-149	544	KY980042.1
SV_912		MAST-12C	NA	NA	NA	NA	Haptoglossa sp.	7e-147	534	KT257372.1 etc.
SV_1117		MAST-12C	NA	NA	NA	uncultured_Eimeriidae	Oblongichytrium sp./Pseudophyllomitus vesiculosus/Mastreximonas tlaamin	5e-148	538	KY980042.1 etc.
SV_3163		MAST-12C	NA	NA	NA	metagenome	Oblongichytrium sp.	2e-146	532	GQ354272.1 etc.
SV_3865		MAST-12C	NA	NA	NA	NA	Lobulomycetales sp.	2e-167	603	OQ702874.1 etc.
SV_3897		MAST-12C	NA	NA	NA	NA	Thalassomyxa sp./Vampyrella sp.	2e-141	516	KC779515.1 etc.
**SV_34**	Peronosporomycetes	NA	NA	NA	NA	NA	Globisporangium glomeratum	0	780	HQ643543.2
**SV_53**		NA	NA	NA	uncultured	metagenome	Myzocytiopsis sp./Lagenidium sp./Phytopythium mirpurense	0	689	KT257374.1 etc.
**SV_57**		NA	NA	NA	Pythium	Pythium_sp.	Pythium sp./Globisporangium sp.	0	782	MT193308.1 etc.
**SV_157**		NA	NA	NA	uncultured	metagenome	Pythium sp./Globisporangium sp.	0	695	KT257379.1 etc.
**SV_169**		NA	NA	NA	NA	NA	Thraustotheca clavata/Achlya bisexualis	0	773	KP098373.1 etc.
SV_211		NA	NA	NA	Pythium	Pythium_sp.	Pythium sp.	0	774	AY598681.2 etc.
SV_268		NA	NA	NA	Pythium	Pythium_sp.	Lagenidium sp./Lagena radicicola	0	769	KJ716870.1 etc.
SV_284		NA	NA	NA	Pythium	NA	Lagenidium giganteum	0	763	KT257365.1 etc.
SV_430		NA	NA	NA	NA	NA	Pythiopsis sp./Achlya sparrowii/Leptolegnia caudata/Saprolegnia sp./Protoachlya paradoxa	0	773	MK850088.1 etc.
SV_437		NA	NA	NA	Pythium	Pythium_sp.	Lagenidium sp./Lagena radicicola	0	780	KJ716873.1 etc.
SV_4846	Preaxostyla	NA	NA	NA	Paratrimastix	Paratrimastix_pyriformis	Kinopus chlorellivorus	1e-153	556	MW694332.1
SV_928	Protalveolata	Perkinsidae	A31	NA	NA	NA	Rana sphenocephala pathogen MJY-2007	0	662	EF675616.1
SV_2045		Perkinsidae	A31	NA	NA	uncultured_eukaryote	Pararosarium dinoexitiosum/Rana sphenocephala pathogen MJY-2007	8e-166	597	MZ663830.1 etc.
SV_2580		Perkinsidae	A31	NA	NA	NA	Pararosarium dinoexitiosum	0	651	MZ663830.1 etc.
SV_2854		Perkinsidae	A31	NA	NA	uncultured_microeukaryote	Rana sphenocephala pathogen MJY-2007	0	647	EF675616.1
SV_2902		Perkinsidae	A31	NA	NA	NA	Rana sphenocephala pathogen MJY-2007	2e-171	616	EF675616.1
SV_2983		Perkinsidae	A31	NA	NA	NA	Rana sphenocephala pathogen MJY-2007	1e-174	627	EF675616.1
SV_3106		Perkinsidae	A31	NA	NA	uncultured_microeukaryote	Rana sphenocephala pathogen MJY-2007	1e-178	640	EF675616.1
SV_3136		Perkinsidae	A31	NA	NA	uncultured_microeukaryote	Rana sphenocephala pathogen MJY-2007	0	651	EF675616.1
SV_3833		Perkinsidae	A31	NA	NA	NA	Rana sphenocephala pathogen MJY-2007	2e-157	569	EF675616.1
SV_4151		Colpodellida	NA	NA	Colpodella	uncultured_freshwater	Alphamonas edax/Olpidium brassicae	0	651	AY234843.1 etc.
SV_1306	Protosporangiida	Protosporangiidae	NA	NA	Protosporangium	metagenome	Asterodiscus tamaricis	2e-52	220	NG_061222.1
SV_1371		Protosporangiidae	NA	NA	Protosporangium	metagenome	Pertusaria saximontana	4e-49	209	AF113720.1
SV_1841		Protosporangiidae	NA	NA	Protosporangium	metagenome	Pertusaria saximontana	4e-54	222	AF113720.1
SV_3213		Protosporangiidae	NA	NA	Protosporangium	metagenome	Tilletiaria anomala/Phragmotaenium derxii/Tilletia iowensis/Heterodoassansia hygrophilae/Hydnum sp.	2e-117	436	NG_061031.1 etc.
SV_1477	Rigifilida	NA	NA	NA	NA	Micronuclearia_podoventralis	Micronuclearia podoventralis	0	745	AY268038.1
SV_1541		NA	NA	NA	NA	Micronuclearia_podoventralis	Micronuclearia podoventralis	0	767	AY268038.1
SV_1794		NA	NA	NA	NA	Micronuclearia_podoventralis	Micronuclearia podoventralis	0	678	AY268038.1
SV_2129		NA	NA	NA	NA	Micronuclearia_podoventralis	Micronuclearia podoventralis	0	712	AY268038.1
SV_4066		NA	NA	NA	NA	Micronuclearia_podoventralis	Micronuclearia podoventralis	0	750	AY268038.1
SV_300	SAR	Labyrinthulomycetes	NA	NA	Sorodiplophrys	metagenome	Sorodiplophrys stercorea	2e-126	466	KU728177.1 etc.
SV_494			NA	NA	Sorodiplophrys	metagenome	Crustomastix sp.	2e-106	399	KY980137.1 etc.
SV_578			NA	NA	Sorodiplophrys	uncultured_eukaryote	Sorodiplophrys stercorea	2e-137	503	KU728177.1 etc.
SV_613			NA	NA	Sorodiplophrys	metagenome	Sorodiplophrys stercorea	1e-129	477	KU728177.1 etc.
SV_652			NA	NA	Sorodiplophrys	metagenome	Sorodiplophrys stercorea	1e-118	440	KU728177.1 etc.
SV_709			NA	NA	Sorodiplophrys	metagenome	Sorodiplophrys stercorea	4e-144	525	KU728177.1 etc.
SV_766			NA	NA	Sorodiplophrys	metagenome	Nerada mexicana	4e-104	392	AY520453.1
SV_806			NA	NA	Sorodiplophrys	uncultured_freshwater	Sorodiplophrys stercorea	0	675	KU728177.1 etc.
SV_908			NA	NA	Sorodiplophrys	metagenome	Sorodiplophrys stercorea	4e-124	459	KU728177.1 etc.
SV_923			NA	NA	Sorodiplophrys	uncultured_freshwater	Sorodiplophrys stercorea	3e-175	628	KU728177.1 etc.
SV_5161	Schizoplasmodiida	NA	NA	NA	Phalansterium	uncultured_eukaryote	Cercomonas sp.	8e-156	564	PP174308.1 etc.

Animalia

ID	Phylum	Class	Order	Family	Genus	Species	The top hits by BLASTN search	e-value	Max score	Accession No.
**SV_1**	Annelida	Clitellata	Haplotaxida	NA	NA	NA	Enchytraeus dichaetus	0	773	MN248684.1
**SV_5**		Clitellata	Haplotaxida	NA	NA	NA	Enchytraeus bulbosus	0	763	GU901825.1
**SV_10**		Clitellata	Haplotaxida	NA	NA	NA	Achaeta sp./Hemienchytraeus sp	0	767	GU901905.1 etc.
**SV_25**		Clitellata	Haplotaxida	NA	NA	Amynthas_sp.	Amynthas sp.	0	771	KF179538.1 etc.
SV_64		Clitellata	Haplotaxida	NA	NA	NA	Fridericia bulboides	0	758	GU901881.1
SV_73		Clitellata	Haplotaxida	NA	NA	NA	Fridericia sp.	0	769	KX618771.1 etc.
SV_98		Clitellata	Haplotaxida	NA	NA	Aeolosoma_sp.	Aeolosoma sp.	0	750	HQ691213.1
SV_119		Clitellata	Haplotaxida	NA	NA	Rheomorpha_neiswestonovae	Rheomorpha neiswestonovae	0	756	AY527049.1
SV_171		Clitellata	NA	NA	NA	NA	Helobdella sp./Oosthuizobdella garoui/Haementeria sp.	0	665	MN312167.1 etc.
SV_199		Clitellata	Haplotaxida	NA	NA	NA	Enchytraeus bulbosus	0	769	GU901872.1
**SV_14**	Arthropoda	Arachnida	NA	NA	NA	Limulidae_environmental	Alicorhagia sp.	0	773	KP325047.1 etc.
**SV_28**		Arachnida	NA	NA	NA	Stigmalychus_sp.	Stigmalychus sp.	0	773	KY922137.1 etc.
**SV_29**		Ellipura	Collembola	NA	NA	NA	Bourletiella hortensis	0	767	KY230700.1
**SV_35**		Arachnida	NA	NA	NA	NA	Pergamasus sp./Vulgarogamasus sp./Aclerogamasus similis/Parasitus kraepelini/Phorytocarpais fimetorum	0	758	FJ911803.1 etc.
SV_54		Arachnida	NA	NA	NA	NA	Phauloppia lucorum/Oribatula tibialis	0	706	EU432198.1 etc.

SV_75		Ellipura	Collembola	NA	NA	NA	Isotomurus sp.	0	734	KY230739.1 etc.
SV_110		Arachnida	NA	NA	NA	NA	Cocceupodes sp.	0	671	MN241880.1
SV_115		Ellipura	Collembola	NA	NA	Lepidocyrtus_sp.	Lepidocyrtus sp.	0	765	KC236249.1
SV_142		Arachnida	NA	NA	NA	NA	Filieupodes cf. filiformis FP101	3e-155	562	MN241882.1
SV_191		Arachnida	NA	NA	NA	NA	Amblyseius sp./Neoseiulus sp./Euseius sojaensis	0	758	KP276469.1 etc.
**SV_51**	Gastrotricha	NA	Chaetonotida	NA	NA	NA	Neogossea sp.	0	780	KR822111.1 etc.
**SV_179**		NA	Chaetonotida	NA	NA	NA	Heterolepidoderma striatum	0	732	OQ358140.1
SV_277		NA	Chaetonotida	NA	NA	Chaetonotus_cf.	Chaetonotus cf. oculifer	0	784	MN496180.1 etc.
SV_299		NA	Chaetonotida	NA	NA	Chaetonotus_cf.	Chaetonotus aff.	0	784	MN496201.1 etc.
SV_378		NA	Chaetonotida	NA	NA	NA	Heterolepidoderma striatum	0	754	OQ358140.1
SV_379		NA	Chaetonotida	NA	NA	NA	Chaetonotus aff.	0	726	MN496229.1 etc.
SV_849		NA	Chaetonotida	NA	NA	NA	Chaetonotus sp.	0	780	MN496226.1 etc.
SV_1290		NA	Chaetonotida	NA	NA	NA	Chaetonotus sp.	0	750	OM421721.1 etc.
SV_1398		NA	Chaetonotida	NA	NA	NA	Heterolepidoderma striatum	0	749	OQ358140.1
SV_1452		NA	Chaetonotida	NA	NA	NA	Bodomorpha sp.	0	763	HM536172.1
SV_433	Mollusca	Gastropoda	NA	NA	NA	NA	Cocceupodes sp./Filieupodes sp.	7e-122	451	MN241880.1 etc.
SV_876		Gastropoda	Heterobranchia	NA	NA	NA	Succinea sp./Monadenia fidelis/Arion hortensis/Bradybaena similaris etc.	0	773	MN186472.1 etc.
SV_3777		Gastropoda	Heterobranchia	NA	NA	NA	Oxychilus alliarius/Vitrina pellucida/Ariophanta bistrialis/Euconulus fulvus/Geomalacus sp.	0	721	MN022707.1 etc.
**SV_23**	Nematozoa	Chromadorea	Araeolaimida	NA	NA	NA	Plectus sp.	0	763	LC186814.1 etc.
**SV_26**		Chromadorea	Rhabditida	NA	NA	NA	Acrobeloides sp.	0	763	MK636581.1 etc.
**SV_32**		Enoplea	NA	NA	NA	NA	Dorylaimellus parvulus	0	763	AY911968.1
**SV_39**		Enoplea	Triplonchida	NA	NA	Prismatolaimus_intermedius	Prismatolaimus sp.	0	763	LC186851.1 etc.
**SV_40**		NA	NA	NA	NA	NA	Diphtherophora sp. Shahrood	1e-168	606	KY115102.1
**SV_42**		Enoplea	NA	NA	NA	Mononchus_truncatus	Mononchus truncatus	0	761	KJ636355.1 etc.
SV_58		Enoplea	NA	NA	NA	Mylonchulus_hawaiiensis	Mylonchulus montanus/Prionchulus oleksandri	0	765	AY919209.1 etc.
SV_84		Chromadorea	Tylenchida	NA	NA	NA	Filenchus sp.	0	767	MN542201.1 etc.
SV_87		Enoplea	NA	NA	NA	Aquatides_christei	Aquatides sp./Clavicaudoides sp.	0	769	AY552967.1 etc.
SV_122		Enoplea	NA	NA	NA	NA	Dorylaimoides sp./Tylencholaimus mirabilis/Mesodorylaimus sp.	0	752	KU662325.1 etc.
**SV_71**	Platyhelminthes	Catenulida	NA	Stenostomidae	NA	Stenostomum_sp.	Stenostomum sp.	0	756	FJ384833.1 etc.
**SV_99**		Catenulida	NA	Stenostomidae	NA	Stenostomum_sp.	Stenostomum sp.	0	739	FJ384833.1 etc.
**SV_100**		Catenulida	NA	Stenostomidae	NA	NA	Stenostomum leucops	0	701	KP113676.1 etc.
**SV_101**		Rhabditophora	Tricladida	NA	NA	Arthurdendyus_triangulatus	Arthurdendyus triangulatus	1e-179	643	OR797297.1
SV_107		Rhabditophora	Tricladida	NA	NA	NA	Arthurdendyus triangulatus	5e-178	638	OR797297.1
SV_108		Rhabditophora	Tricladida	NA	NA	NA	Girardia sp.	0	747	MN652491.1 etc.
SV_130		Rhabditophora	Tricladida	NA	NA	NA	Arthurdendyus triangulatus	5e-178	638	OR797297.1
SV_146		Rhabditophora	Rhabdocoela	NA	NA	NA	Bryoplana xerophila	0	713	KC529489.1
SV_188		Catenulida	NA	Catenulidae	NA	Catenula_turgida	Retronectes sp.	0	649	OR096307.1 etc.
SV_223		Rhabditophora	Rhabdocoela	NA	NA	Acrochordonoposthia_conica	Acrochordonoposthia conica	0	798	KC529487.1
SV_589	Rotifera	Monogononta	Ploimida	NA	NA	NA	Lecane sp.	0	760	MT025820.1 etc.
SV_1479		Monogononta	Ploimida	NA	NA	NA	Cephalodella gibba	0	726	AY218114.1
SV_1543		Monogononta	Ploimida	NA	NA	NA	Synchaeta sp./Asplanchna girodi	0	754	KY751522.1 etc.
SV_1940		Monogononta	Ploimida	NA	NA	uncultured_eukaryote	Rhinoglena frontalis/Encentrum semiplicatum	0	732	MT522683.1 etc.
SV_1993		Monogononta	Ploimida	NA	NA	uncultured_eukaryote	Bryceella stylata	0	710	MT522631.1
SV_2285		Monogononta	Ploimida	NA	NA	NA	Encentrum semiplicatum	0	699	MT522645.1
SV_2780		Bdelloidea	Adinetida	NA	NA	Bdelloidea_environmental	Rotaria sp./Habrotrocha sp./Adineta vaga	0	761	JX494744.2 etc.
SV_2921		Monogononta	Ploimida	NA	NA	NA	Mikrocodides chlaena	0	732	MT522662.1
SV_2944		Monogononta	Ploimida	NA	NA	NA	Brachionus zahniseri	0	721	MT522630.1
SV_3022		Monogononta	Ploimida	NA	NA	NA	Kellicottia bostoniensis	0	737	MT522652.1
SV_1468	Tardigrada	Eutardigrada	Parachela	NA	NA	NA	Doryphoribius sp.	0	761	MT872215.1
SV_3197		Eutardigrada	Parachela	NA	NA	NA	Apodibius confusus	0	769	KC582830.1

Plants

ID	Phylum	Class	Order	Family	Genus	Species	The top hits by BLASTN search	e-value	Max score	Accession No.
**SV_2**	Phragmoplastophyta	Embryophyta	NA	NA	NA	Oryza_sativa	Oryza sp./Setaria viridis/Panicum hallii etc.	0	773	XR_008019782.1 etc.
**SV_4**		Embryophyta	NA	NA	NA	NA	Brassica napus/Arabidopsis thaliana	0	769	CP151913.1 etc.
**SV_6**		Embryophyta	NA	NA	NA	NA	Cerastium arcticum	0	771	EF127844.1
**SV_11**		Embryophyta	NA	NA	NA	NA	Bidens sp.	0	771	MN535784.1 etc.
**SV_33**		Embryophyta	NA	NA	NA	Triticum_aestivum	Hordeum vulgare subsp. vulgare	0	773	XR_006618434.1
**SV_50**		Embryophyta	NA	NA	NA	NA	Sisyrinchium campestre	0	769	MT610957.1
SV_72		Embryophyta	NA	NA	NA	NA	Silene littorea	0	767	MN325962.1
SV_76		Embryophyta	NA	NA	NA	NA	Diplostephium sp./Linochilus sp./Solidago sp./Artemisia dracunculus etc.	0	771	MT610965.1 etc.
SV_78		Embryophyta	NA	NA	NA	NA	Ptychomitrium gardneri/Ceratodon sp./Fissidens subbasilaris/Tortella tortuosa	0	778	MN307174.1 etc.
SV_81		Embryophyta	NA	NA	NA	NA	Panax sp.	0	765	MK408812.1 etc.

Supplementary Table S5. Cp-values and feeding types of nematode-derived SVs. Class, order, and species of nematode-derived SVs based on the SILVA database. The genus of the hit with the lowest e-value by BLASTN search (May 2024) is shown. The genera derived from the hits with the same lowest e-values are separated by forward slash. The cp-value and feeding type were determined based on the genus of top hit by BLASTN search in the Nematode Ecophysiological Parameter Search at the Nemaplex homepage of UC Davis, USA (http://nemaplex.ucdavis.edu/Ecology/EcophysiologyParms/EcoParameterMenu.html). NA: not assigned. ND: no data. ap: animal-parasitic nematodes. m: marine nematode. The feeding type of the family was used if the genera are absent in nematode genus list (i.e., cp-value is zero).

ID	Class	Order	Species	Genus of top hits by BLASTN search	cp-value	Feeding type
SV_23	Chromadorea	Araeolaimida	NA	*Plectus*	2	3
SV_26	Chromadorea	Rhabditida	NA	*Acrobeloides*	2	3
SV_32	Enoplea	NA	NA	*Dorylaimellus*	5	1
SV_39	Enoplea	Triplonchida	Prismatolaimus_intermedius	*Prismatolaimus*	3	3
SV_40	NA	NA	NA	*Diphtherophora*	3	2
SV_42	Enoplea	NA	Mononchus_truncatus	*Mononchus*	4	5
SV_58	Enoplea	NA	Mylonchulus_hawaiiensis	*Prionchulus*	4	5
SV_84	Chromadorea	Tylenchida	NA	*Filenchus*	2	2
SV_87	Enoplea	NA	Aquatides_christei	*Clavicaudoides/Aquatides*	5	5
SV_122	Enoplea	NA	NA	*Dorylaimoides*	4	8
SV_132	Enoplea	Triplonchida	Odontolaimus_sp.	*Odontolaimus*	3	3
SV_155	Enoplea	NA	NA	*Amblydorylaimus/Eudorylaimus/Talanema*	4	8
SV_175	Chromadorea	Tylenchida	NA	*Meloidogyne*	3	1
SV_198	Chromadorea	Monhysterida	Paralamyctes_environmental	*Eumonhystera*	2	3
SV_200	Enoplea	Triplonchida	Squillidae_environmental	*Prismatolaimus*	3	3
SV_205	Enoplea	NA	NA	*Axonchoides*	5	1
SV_231	Enoplea	Triplonchida	Odontolaimus_sp.	*Odontolaimus*	3	3
SV_234	Chromadorea	Tylenchida	Pratylenchus_delattrei	*Pratylenchus*	3	1
SV_237	Chromadorea	Chromadorida	Prodesmodora_circulata	*Achromadora*	3	5
SV_250	Chromadorea	Monhysterida	Paralamyctes_environmental	*Eumonhystera*	2	3
SV_276	Enoplea	NA	Mylonchulus_arenicolus	*Mylonchulus*	4	5
SV_320	Chromadorea	Tylenchida	Aphelenchus_avenae	*Aphelenchus*	2	2
SV_331	Chromadorea	Tylenchida	Filenchus_discrepans	*Filenchus*	2	2
SV_332	Enoplea	Triplonchida	Prismatolaimus_intermedius	*Prismatolaimus*	3	3
SV_360	Chromadorea	Tylenchida	Ditylenchus_sp.	*Ditylenchus*	2	2
SV_371	Chromadorea	Tylenchida	NA	*Ditylenchus*	2	2
SV_396	Enoplea	NA	NA	*Tylencholaimus*	4	2
SV_405	Chromadorea	Monhysterida	NA	*Eumonhystera*	2	3
SV_413	Enoplea	NA	Limulidae_environmental	*Aporcelaimellus*	5	8
SV_419	Chromadorea	Tylenchida	Pratylenchus_delattrei	*Pratylenchus*	3	1
SV_450	Chromadorea	Tylenchida	NA	*Aphelenchoides*	2	2
SV_454	Enoplea	Triplonchida	Prismatolaimus_intermedius	*Prismatolaimus*	3	3
SV_457	Enoplea	NA	Eudorylaimus_carteri	*Thonus*	4	5
SV_468	Chromadorea	Monhysterida	NA	*Sabatieria*	2	m
SV_492	Chromadorea	Rhabditida	NA	*Eucephalobus/Cephalobus*	2	3
SV_541	Enoplea	Triplonchida	Prismatolaimus_intermedius	*Prismatolaimus*	3	3
SV_550	Enoplea	NA	Mononchus_aquaticus	*Mononchus*	4	5
SV_579	Enoplea	Triplonchida	Odontolaimus_sp.	*Odontolaimus*	3	3
SV_599	Enoplea	NA	NA	*Dorylaimoides*	4	8
SV_600	Chromadorea	Monhysterida	NA	*Eumonhystera*	2	3
SV_646	Enoplea	NA	NA	*Pararhyssocolpus*	4	8
SV_670	Chromadorea	Chromadorida	Achromadora_cf	*Achromadora*	3	5
SV_672	Enoplea	Triplonchida	Prismatolaimus_cf.	*Prismatolaimus*	3	3
SV_694	Enoplea	NA	NA	*Mesodorylaimus*	4	8
SV_752	Chromadorea	Monhysterida	NA	*Eumonhystera*	2	3
SV_764	Chromadorea	Rhabditida	Oscheius_tipulae	*Oscheius*	1	3
SV_802	Enoplea	Triplonchida	Prismatolaimus_intermedius	*Prismatolaimus*	3	3
SV_804	Chromadorea	Tylenchida	Aphelenchoides_fujianensis	*Aphelenchoides*	2	2
SV_828	Chromadorea	Araeolaimida	Anaplectus_grandepapillatus	*Plectus*	2	3
SV_845	Enoplea	Triplonchida	NA	*Diphtherophora*	3	2
SV_846	Chromadorea	Tylenchida	NA	*Aphelenchoides*	2	2
SV_875	Chromadorea	Tylenchida	NA	*Meloidogyne*	3	1
SV_885	Chromadorea	Tylenchida	Meloidogyne_incognita	*Meloidogyne*	3	1
SV_898	Enoplea	NA	Mononchus_aquaticus	*Mononchus*	4	5
SV_902	Enoplea	NA	NA	*Bastiania*	3	3

SV_927	Chromadorea	Rhabditida	Cephalobus_cubaensis	*Cephalobus*	2	3
SV_934	Chromadorea	Tylenchida	NA	*Aphelenchoides*	2	2
SV_949	Chromadorea	Tylenchida	Irantylenchus_vicinus	*Irantylenchus*	2	1
SV_960	Chromadorea	Tylenchida	Filenchus_discrepans	*Filenchus*	2	2
SV_991	Chromadorea	Tylenchida	NA	*Basiria*	2	1
SV_1022	Chromadorea	Tylenchida	NA	*Basiria*	2	1
SV_1046	Chromadorea	Araeolaimida	NA	*Plectus*	2	3
SV_1047	Chromadorea	Tylenchida	Aphelenchus_avenae	*Aphelenchus*	2	2
SV_1054	Chromadorea	Tylenchida	NA	*Lelenchus*	2	1
SV_1055	Chromadorea	Tylenchida	NA	*Meloidogyne*	3	1
SV_1059	Enoplea	NA	NA	*Microdorylaimus*	4	8
SV_1084	Chromadorea	Tylenchida	NA	*Aphelenchoides*	2	2
SV_1100	Chromadorea	Chromadorida	Prodesmodora_circulata	*Achromadora*	3	5
SV_1134	Chromadorea	Tylenchida	Basiria_sp.	*Basiria*	2	1
SV_1162	Chromadorea	Tylenchida	Aphelenchoides_bicaudatus	*Aphelenchoides*	2	2
SV_1174	Enoplea	Triplonchida	Odontolaimus_sp.	*Diphtherophora*	3	2
SV_1175	Chromadorea	Monhysterida	Paralamyctes_environmental	*Eumonhystera*	2	3
SV_1192	Enoplea	Triplonchida	NA	*Odontolaimus*	3	3
SV_1199	Enoplea	NA	Mylonchulus_brachyuris	*Mylonchulus*	4	5
SV_1207	Enoplea	NA	NA	*Mesodorylaimus*	4	8
SV_1211	Chromadorea	Tylenchida	NA	*Lelenchus*	2	1
SV_1220	Enoplea	NA	NA	*Discolaimus*	4	8
SV_1256	Chromadorea	Tylenchida	NA	*Lelenchus*	2	1
SV_1304	Enoplea	NA	NA	*Aporcelaimellus*	5	8
SV_1307	Chromadorea	Tylenchida	Pratylenchus_penetrans	*Pratylenchus*	3	1
SV_1335	Chromadorea	Tylenchida	Filenchus_discrepans	*Filenchus*	2	2
SV_1404	Chromadorea	Tylenchida	NA	*Geocenamus*	3	1
SV_1433	Enoplea	NA	NA	*Tylencholaimellus*	4	2
SV_1562	Enoplea	NA	Eudorylaimus_carteri	*Eudorylaimus*	4	8
SV_1574	Chromadorea	Monhysterida	NA	*Eumonhystera*	2	3
SV_1597	Enoplea	Triplonchida	NA	*Diphtherophora*	3	2
SV_1613	Chromadorea	Tylenchida	Aphelenchoides_fujianensis	*Aphelenchoides*	2	2
SV_1622	Chromadorea	Tylenchida	Basiria_sp.	*Basiria*	2	1
SV_1635	Chromadorea	Chromadorida	Prodesmodora_circulata	*Achromadora*	3	5
SV_1652	Enoplea	NA	Mylonchulus_brachyuris	*Mylonchulus*	4	5
SV_1657	Chromadorea	Tylenchida	NA	*Tylenchus/Filenchus*	2	1, 2
SV_1695	Enoplea	NA	NA	*Clavicaudoides*	5	5
SV_1753	Chromadorea	Tylenchida	Malenchus_sexlineatus	*Malenchus*	2	1
SV_1760	Enoplea	NA	Nematoda_sp.	*Gastromermis*	0	ap
SV_1785	Chromadorea	Tylenchida	Filenchus_misellus	*Filenchus*	2	2
SV_1810	Chromadorea	Rhabditida	Poikilolaimus_oxycercus	*Poikilolaimus*	1	3
SV_1860	Chromadorea	Chromadorida	Achromadora_cf	*Achromadora*	3	5
SV_1879	Chromadorea	Tylenchida	Pratylenchus_delattrei	*Pratylenchus*	3	1
SV_1922	Enoplea	Triplonchida	Prismatolaimus_intermedius	*Prismatolaimus*	3	3
SV_1986	Chromadorea	Tylenchida	NA	*Lelenchus*	2	1
SV_1992	Enoplea	NA	NA	*Nematimermis*	0	ND
SV_2013	Chromadorea	Tylenchida	Aphelenchoides_bicaudatus	*Aphelenchoides*	2	2
SV_2021	Chromadorea	Monhysterida	NA	*Eumonhystera*	2	3
SV_2036	Enoplea	NA	NA	*Paractinolaimus*	5	5
SV_2043	Chromadorea	Tylenchida	NA	*Ditylenchus*	2	2
SV_2055	Enoplea	NA	Eudorylaimus_carteri	*Prodorylaimus*	4	8
SV_2087	Chromadorea	Tylenchida	NA	*Lelenchus*	2	1
SV_2155	Enoplea	NA	NA	*cf. Tylencholaimus/cf. Calodorylaimus*	4	2
SV_2170	Chromadorea	Monhysterida	NA	*Eumonhystera*	2	3
SV_2174	Chromadorea	Araeolaimida	NA	*Plectus*	2	3
SV_2202	Chromadorea	Monhysterida	NA	*Eumonhystera*	2	3
SV_2212	Chromadorea	Tylenchida	NA	*Nothotylenchus*	2	2
SV_2242	Enoplea	NA	NA	*Microdorylaimus*	4	8
SV_2271	Chromadorea	Araeolaimida	Anaplectus_grandepapillatus	*Anaplectus*	2	3
SV_2301	Enoplea	NA	NA	*Tylencholaimus*	4	2
SV_2304	Chromadorea	Tylenchida	NA	*Lelenchus*	2	1
SV_2348	Chromadorea	Tylenchida	Aphelenchoides_sp.	*Aphelenchoides*	2	2

SV_2356	Chromadorea	Chromadorida	Achromadora_cf	*Achromadora*	3	5
SV_2423	Chromadorea	Tylenchida	NA	*Miculenchus*	2	1
SV_2470	Enoplea	NA	NA	*Laimydorus*	4	8
SV_2478	Chromadorea	Tylenchida	Ditylenchus_halictus	*Ditylenchus*	2	2
SV_2510	Chromadorea	Tylenchida	Aphelenchoides_bicaudatus	*Aphelenchoides*	2	2
SV_2518	Chromadorea	Tylenchida	Ditylenchus_halictus	*Ditylenchus*	2	2
SV_2538	Chromadorea	Monhysterida	uncultured_eukaryote	*Eumonhystera*	2	3
SV_2629	Enoplea	NA	NA	*Prionchulus*	4	5
SV_2643	Chromadorea	Tylenchida	NA	*Filenchus*	2	2
SV_2648	Chromadorea	Monhysterida	Nematoda_environmental	*Eumonhystera*	2	3
SV_2658	Chromadorea	Chromadorida	NA	*Achromadora*	3	5
SV_2662	Enoplea	NA	NA	*Heterodorus/Longidorella/Microdorylaimus/Allodorylaimus/Eudorylaimus*	4	8
SV_2686	Chromadorea	Tylenchida	NA	*Discopersicus/Basiria*	2	1
SV_2758	Enoplea	Triplonchida	NA	*Trischistoma*	3	5
SV_2760	Enoplea	NA	NA	*Amblydorylaimus/Eudorylaimus/Talanema*	4	8
SV_2781	Chromadorea	Tylenchida	NA	*Psilenchus*	2	1
SV_2812	Enoplea	NA	NA	*Tylencholaimus*	4	2
SV_2858	Chromadorea	Tylenchida	NA	*Ficophagus/Schistonchus*	2	1
SV_2864	Chromadorea	Monhysterida	NA	*Eumonhystera*	2	3
SV_2867	Chromadorea	Tylenchida	NA	*Meloidogyne*	3	1
SV_2923	Chromadorea	Tylenchida	NA	*Boleodorus*	2	1
SV_2933	Chromadorea	Rhabditida	NA	*Cephalobus*	2	3
SV_2934	Chromadorea	Araeolaimida	Anaplectus_grandepapillatus	*Anaplectus*	2	3
SV_2939	Enoplea	Triplonchida	Tripylina_sp.	*Tripylina*	3	5
SV_2977	Chromadorea	Tylenchida	NA	*Meloidogyne*	3	1
SV_3050	Chromadorea	Chromadorida	NA	*Achromadora*	3	5
SV_3056	Chromadorea	Chromadorida	NA	*Achromadora*	3	5
SV_3090	Chromadorea	Rhabditida	NA	*Distolabrellus*	1	3
SV_3093	Chromadorea	Tylenchida	Pratylenchus_delattrei	*Pratylenchus*	3	1
SV_3104	Chromadorea	Tylenchida	NA	*Psilenchus*	2	1
SV_3112	Chromadorea	Tylenchida	Filenchus_misellus	*Filenchus*	2	2
SV_3201	Chromadorea	Rhabditida	NA	*Panagrolaimus*	1	3
SV_3233	Enoplea	NA	NA	*Axonchium*	5	1
SV_3305	Chromadorea	Tylenchida	NA	*Labrys*	2	1
SV_3311	Chromadorea	Tylenchida	Pratylenchus_delattrei	*Pratylenchus*	3	1
SV_3317	Chromadorea	Rhabditida	Mesorhabditis_sp.	*Mesorhabditis*	1	3
SV_3380	Enoplea	Triplonchida	Trischistoma_taiguensis	*Trischistoma*	3	5
SV_3382	Chromadorea	Rhabditida	Mesorhabditis_sp.	*Mesorhabditis*	1	3
SV_3397	Chromadorea	Tylenchida	NA	*Pratylenchus*	3	1
SV_3399	Chromadorea	Araeolaimida	NA	*Plectus*	2	3
SV_3414	Chromadorea	Tylenchida	NA	*Pratylenchus*	3	1
SV_3502	Chromadorea	Rhabditida	NA	*Cervidellus/Heterocephalobus*	2	3
SV_3512	Chromadorea	Tylenchida	NA	*Labrys*	2	1
SV_3566	Chromadorea	Araeolaimida	Anaplectus_grandepapillatus	*Anaplectus*	2	3
SV_3627	Chromadorea	Rhabditida	NA	*Helicotylenchus*	3	1
SV_3635	Chromadorea	Tylenchida	NA	*Pratylenchus*	3	1
SV_3650	Chromadorea	Rhabditida	Propanagrolaimus_sp.	*Panagrolaimus*	1	3
SV_3684	Chromadorea	Monhysterida	NA	*Eumonhystera*	2	3
SV_3748	Chromadorea	Tylenchida	NA	*Malenchus*	2	1
SV_3797	Enoplea	NA	NA	*Clarkus/Actus*	4	5
SV_3838	Chromadorea	Monhysterida	Eumonhystera_cf.	*Eumonhystera*	2	3
SV_3948	Chromadorea	Monhysterida	NA	*Eumonhystera*	2	3
SV_3960	Chromadorea	Tylenchida	Filenchus_discrepans	*Sakia*	2	1
SV_4048	Enoplea	NA	NA	*Xiphinemella*	4	2
SV_4127	Chromadorea	Monhysterida	NA	*Eumonhystera*	2	3
SV_4247	Chromadorea	Tylenchida	Filenchus_vulgaris	*Mylonchulus*	4	5
SV_4260	Chromadorea	Rhabditida	NA	*Acrobeles*	2	3
SV_4288	Chromadorea	Rhabditida	NA	*Acrobeles*	2	3
SV_4322	Chromadorea	Monhysterida	uncultured_eukaryote	*Eumonhystera*	2	3
SV_4507	Chromadorea	Tylenchida	Aphelenchus_avenae	*Aphelenchus*	2	2
SV_4790	Chromadorea	Tylenchida	NA	*Meloidogyne*	3	1

SV_4795	Chromadorea	Tylenchida	Filenchus_vulgaris	*Filenchus*	2	2
SV_4810	Chromadorea	Tylenchida	NA	*Meloidogyne*	3	1
SV_4811	Chromadorea	Tylenchida	NA	*Meloidogyne*	3	1
SV_4812	Chromadorea	Tylenchida	NA	*Meloidogyne*	3	1
SV_4829	Enoplea	NA	NA	*Aporcella/Dorylaimellus*	5	1, 8
SV_4853	Chromadorea	Rhabditida	Cephalobus_cubaensis	*Cephalobus*	2	3
SV_4854	Chromadorea	Tylenchida	NA	*Filenchus*	2	2
SV_4875	Enoplea	NA	NA	*Clavicaudoides*	5	5
SV_4918	Chromadorea	NA	NA	*Achromadora*	3	5
SV_5078	Enoplea	NA	NA	*Microdorylaimus/Eudorylaimus*	4	8
SV_5101	Chromadorea	Rhabditida	Cephalobus_cubaensis	*Cephalobus*	2	3

Supplementary Table S6. Parameter values of prokaryotic and eukaryotic networks of top 150 abundant SVs in each sample. The values of the parameters of the prokaryotic and eukaryotic co-occurence networks of top 150 abundant SVs are shown in each sample. Networks were prepared as described in the Methods section.

Prokaryotic network (top150 abundant SVs, r = 0.8, p = 0.01)

Papameters	February	April	May	June	August
Number of links	316	346	262	344	298
Number of positive links	268	314	165	247	246
Number of negative links	48	32	97	97	52
Number of vertices	132	133	131	129	139
Connectance (link_density)	0.037	0.039	0.031	0.042	0.031
Average degree (Average K)	4.788	5.203	4.000	5.333	4.288
Average path length	6.032	5.204	4.807	6.432	5.715
Diameter	16.024	14.967	13.131	22.467	15.958
Mean clustering coefficient (Average.CC)	0.541	0.577	0.646	0.600	0.548
Number of clusters	12	10	13	13	10
Centralization degree	0.070	0.059	0.077	0.099	0.049
Centralization betweenness	0.228	0.119	0.087	0.177	0.121
Centralization closeness	1.371	1.347	1.231	1.346	1.385
Relative modularity (RM)	0.727	0.975	0.931	0.702	0.732
The number of keystone nodes	1	5	1	2	1

Eukaryotic network (top150 abundant SVs, r = 0.7, p = 0.01)

Papameters	February	April	May	June	August
Number of links	288	325	485	266	314
Number of positive links	208	302	478	246	222
Number of negative links	80	23	7	20	92
Number of vertices	135	137	135	126	137
Connectance (link_density)	0.032	0.035	0.054	0.034	0.034
Average degree (Average K)	4.267	4.745	7.185	4.222	4.584
Average path length	5.720	5.081	4.281	5.779	6.617
Diameter	14.075	12.279	11.318	15.033	17.843
Mean clustering coefficient (Average.CC)	0.518	0.643	0.573	0.628	0.550
Number of clusters	9	12	10	11	9
Centralization degree	0.080	0.046	0.088	0.078	0.099
Centralization betweenness	0.242	0.182	0.091	0.218	0.252
Centralization closeness	1.420	1.275	1.329	1.319	1.439
Relative modularity (RM)	0.896	1.087	1.142	0.993	0.909
The number of keystone nodes	1	4	4	0	0
