# Supplementary material for: Dynamic Changes in Prokaryotic and Eukaryotic Communities and Networks in Minimally Managed Cabbage-Cultivated Field Soils
Source: Genes (Basel). 2025 Apr 24;16(5):482. doi: 10.3390/genes16050482 (PMC12111001; doi:10.3390/genes16050482)
Supplement: Supplementary file 1 [file genes-16-00482-s001.zip › Suppl Materials/Sup Figs_Genes.pdf]

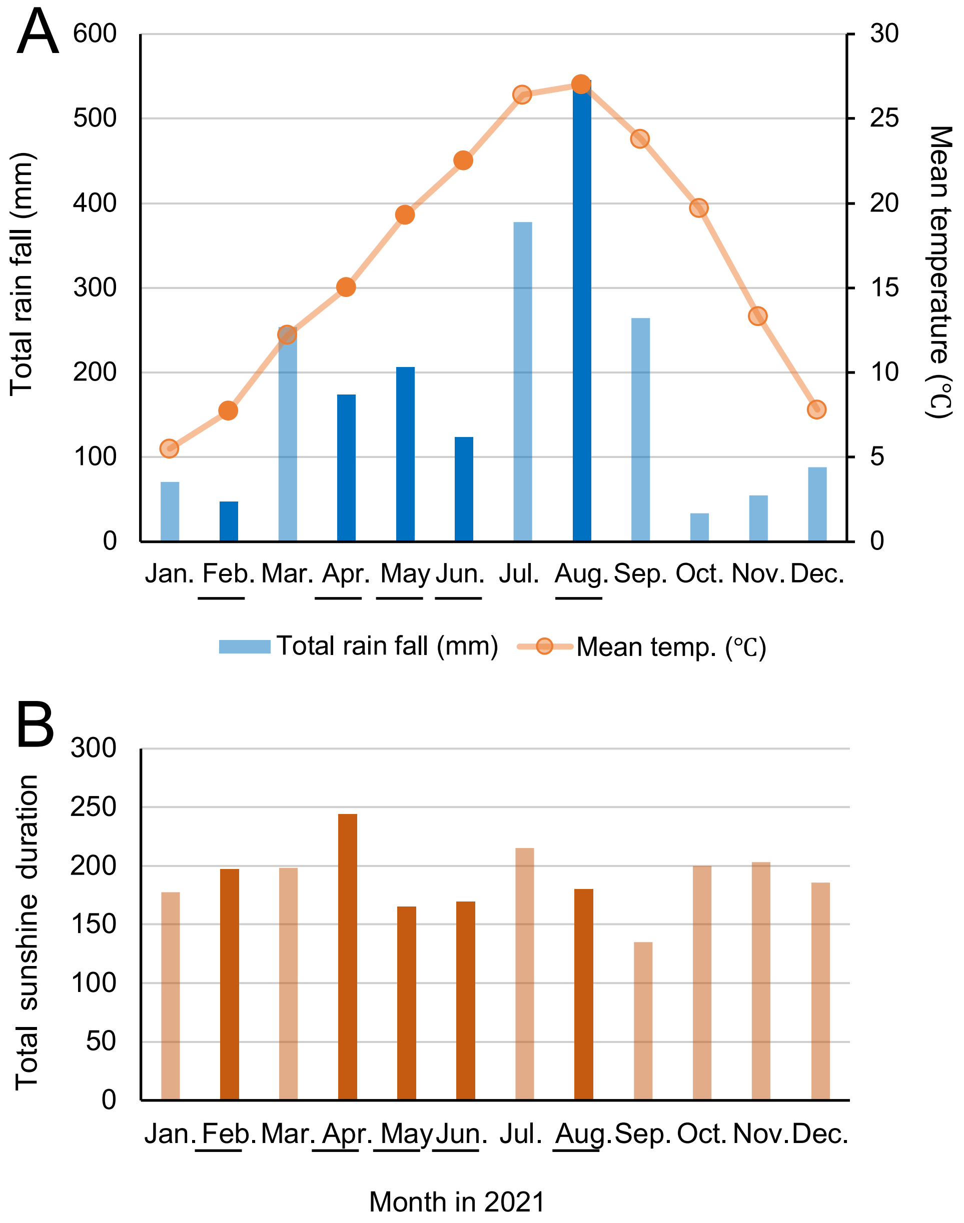

Figure S1. Total rainfall and mean temperature and total sunshine duration per month at Toyohashi city in 2021. The total rainfall (mm) and mean temperature (°C) per month, and total sunshine duration (h) in Toyohashi city in 2021 are shown by bar and line graphs in panel (A) and (B), respectively. Data was obtained from home page of Japan Meteorological Agency (https://www.data.jma.go.jp/obd/stats/etrn/view/monthly_a1.php?prec_no=51&block_no=0470&year=2021&month=&day=&view=p1). Note: Total sunshine durations of January to March were measured by different type of sunshine meter.

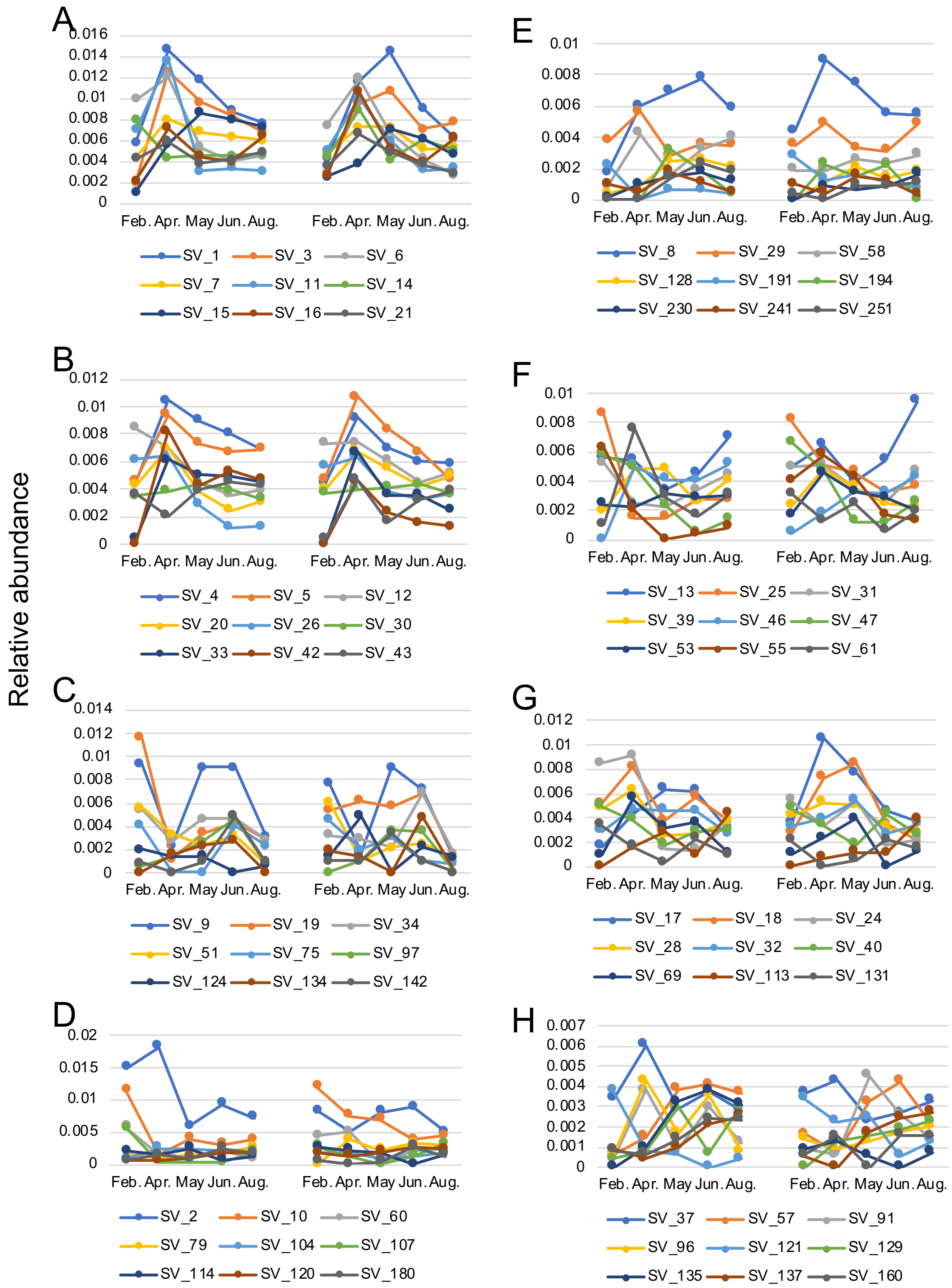
Relative abundance
A
0.016
0.014
0.012
0.01
0.008
0.006
0.004
0.002
0
Feb. Apr. May Jun. Aug. Feb. Apr. May Jun. Aug.
SV_1 SV_3 SV_6
SV_7 SV_11 SV_14
SV_15 SV_16 SV_21
B
0.012
0.01
0.008
0.006
0.004
0.002
0
Feb. Apr. May Jun. Aug. Feb. Apr. May Jun. Aug.
SV_4 SV_5 SV_12
SV_20 SV_26 SV_30
SV_33 SV_42 SV_43
C
0.014
0.012
0.01
0.008
0.006
0.004
0.002
0
Feb. Apr. May Jun. Aug. Feb. Apr. May Jun. Aug.
SV_9 SV_19 SV_34
SV_51 SV_75 SV_97
SV_124 SV_134 SV_142
D
0.02
0.015
0.01
0.005
0
Feb. Apr. May Jun. Aug. Feb. Apr. May Jun. Aug.
SV_2 SV_10 SV_60
SV_79 SV_104 SV_107
SV_114 SV_120 SV_180
E
0.01
0.008
0.006
0.004
0.002
0
Feb. Apr. May Jun. Aug. Feb. Apr. May Jun. Aug.
SV_8 SV_29 SV_58
SV_128 SV_191 SV_194
SV_230 SV_241 SV_251
F
0.01
0.008
0.006
0.004
0.002
0
Feb. Apr. May Jun. Aug. Feb. Apr. May Jun. Aug.
SV_13 SV_25 SV_31
SV_39 SV_46 SV_47
SV_53 SV_55 SV_61
G
0.012
0.01
0.008
0.006
0.004
0.002
0
Feb. Apr. May Jun. Aug. Feb. Apr. May Jun. Aug.
SV_17 SV_18 SV_24
SV_28 SV_32 SV_40
SV_69 SV_113 SV_131
H
0.007
0.006
0.005
0.004
0.003
0.002
0.001
0
Feb. Apr. May Jun. Aug. Feb. Apr. May Jun. Aug.
SV_37 SV_57 SV_91
SV_96 SV_121 SV_129
SV_135 SV_137 SV_160

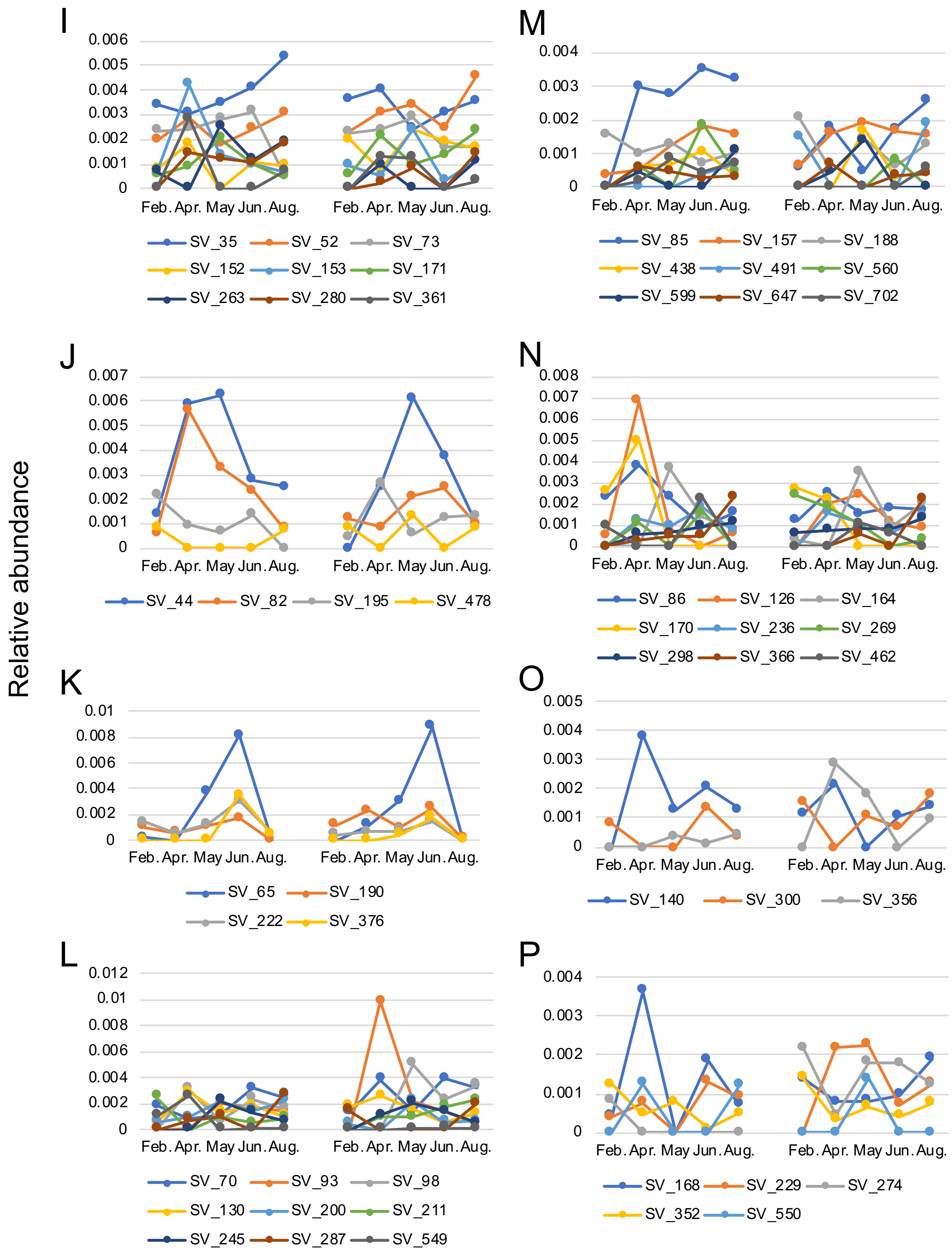

Figure S2. Changes in the relative abundances of abundant prokaryotic SVs in each phylum. The relative abundances of abundant SVs shown in the bottom of the panels of Proteobacteria (A), Acidobacteriota (B), Actinobacteriota (C), Chloroflexi (D), Bacteroidota (E), Gemmatimonadota (F), Verrucomicrobiota (G), Planctomycetota (H), Latescibacterota (I), Nitrospirota (J), Cyanobacteria (K), Methylomirabilota (L), Armatimonadota (M), Myxococcota (N), Desulfobacterota (O), and NB1-j (P), are indicated during the cultivation period (see Table S1). The left and right line graphs indicate the results from control and plant sites, respectively.

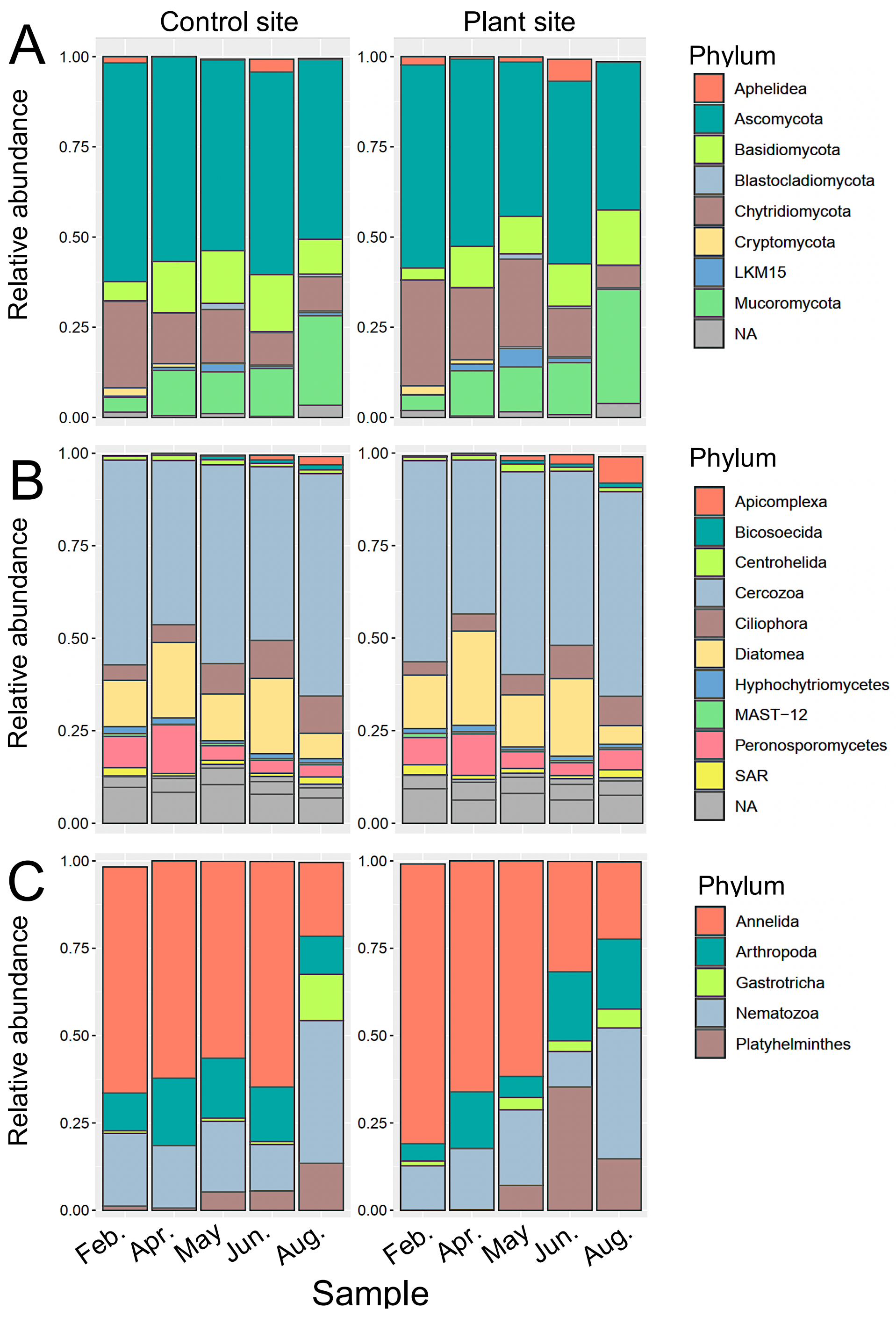

Figure S3. Phylum compositions of fungi-, protists-, and animal-derived SVs across the cultivation period. Compositions at control and plant sites are shown on the left and right, respectively. The SILVA-based taxa are indicated by the colors shown on the right of the figures. The relative abundances of the SVs at phylum levels in each sample are shown by bar charts: kingdom Fungi (A), protists (i.e., kingdoms Alveolata, Amoebozoa, Rhizaria, and Stramenopiles, phyla Nucleariidae_and_Fonticula_group, and Preaxostyla, and classes Choanoflagellida, Filasterea, and Ichthyosporea) (B), and kingdom Animalia (C), respectively with a relative abundance more than 0.5%. NA, not assigned phylum.

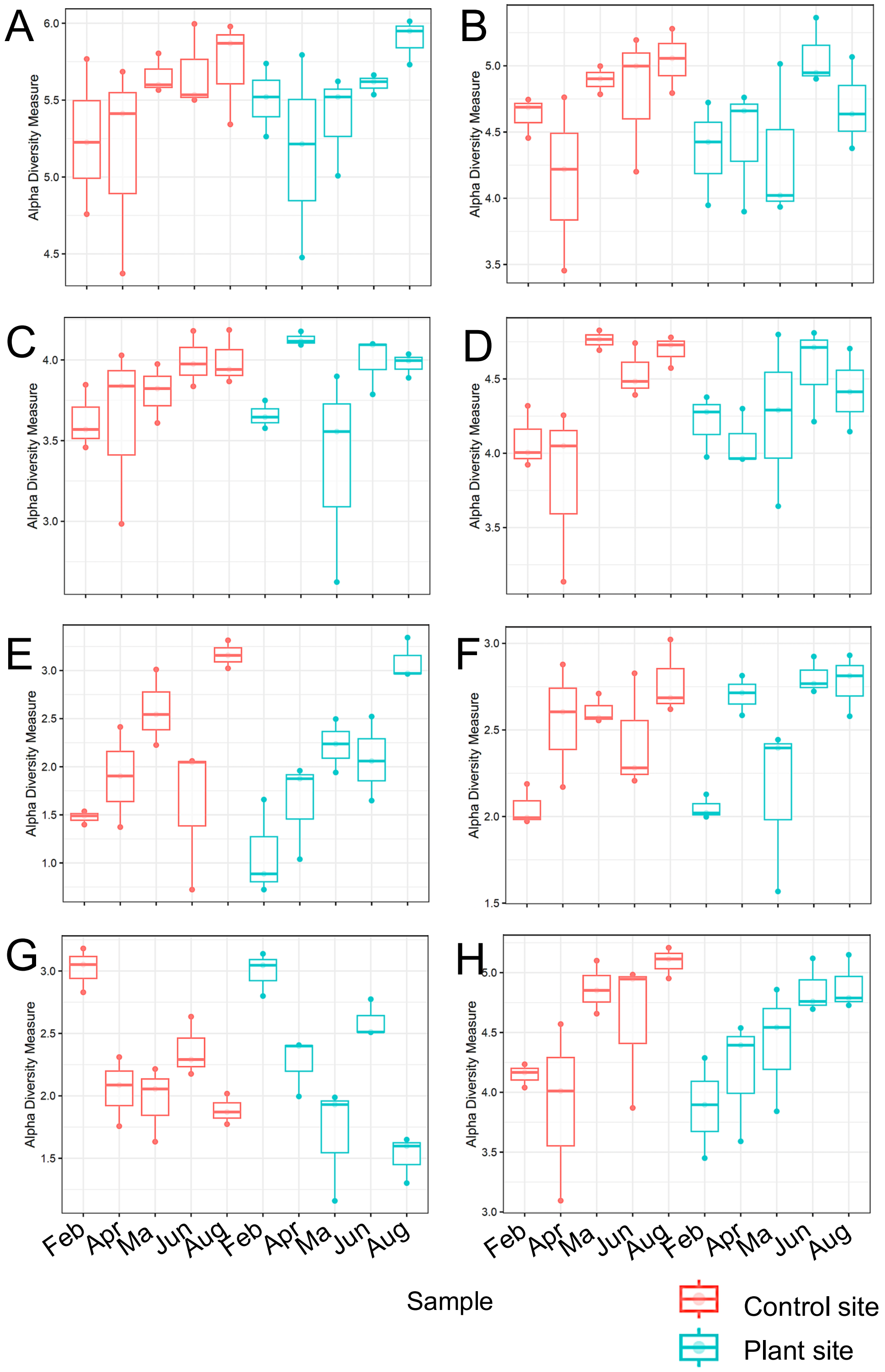

Figure S4. Boxplots showing the α-diversities by the Shannon index across the five samples. The α-diversities of prokaryote (A), eukaryote (B), fungi (C), protists (D), animals (E), nematodes (F), plants (G), and eukaryotes without plants (H) across the cultivation period (month) are indicated in each sample derived from control (left) and plant (right) sites by boxplots. The taxa of fungi, protists, and animals are referred to in the legend for Fig. S3, and those of nematodes and plants represent the SVs derived from phyla Nematozoa and Phragmoplastophyta, respectively.

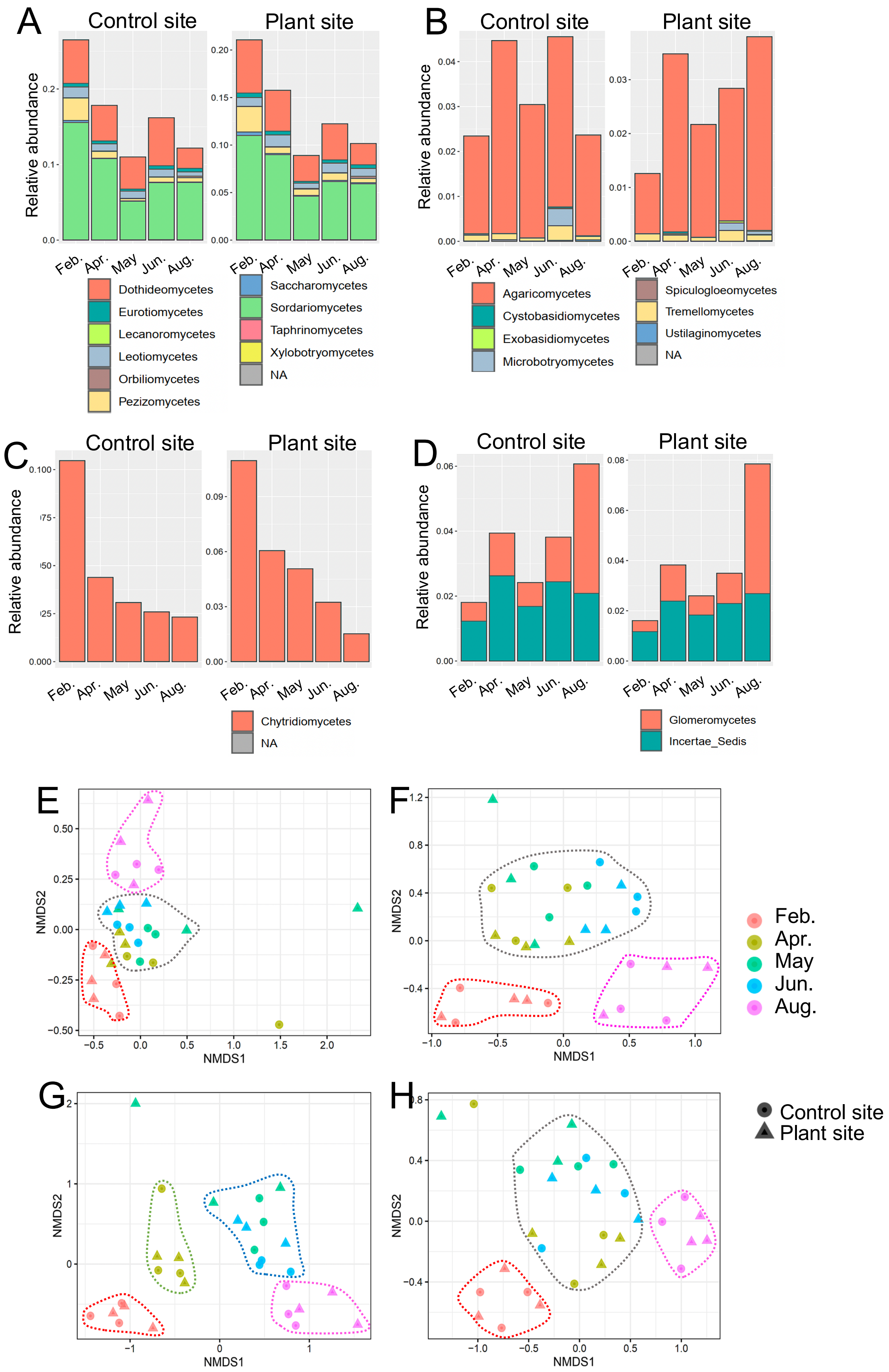
A
Control site
Plant site
Relative abundance
Feb.
Apr.
May
Jun.
Aug.
Dothideomycetes
Eurotiomycetes
Lecanoromycetes
Leotiomycetes
Orbiliomycetes
Pezizomycetes
Saccharomycetes
Sordariomycetes
Taphrinomycetes
Xylobotryomycetes
NA
B
Control site
Plant site
Relative abundance
Agaricomycetes
Cystobasidiomycetes
Exobasidiomycetes
Microbotryomycetes
Spiculogloeomycetes
Tremellomycetes
Ustilaginomycetes
NA
C
Control site
Plant site
Relative abundance
Chytridiomycetes
NA
D
Control site
Plant site
Relative abundance
Glomeromycetes
Incertae_Sedis
E
F
G
H
NMDS1
NMDS2
Feb.
Apr.
May
Jun.
Aug.
Control site
Plant site

Figure S5. Taxonomic compositions and nonmetric multidimensional scaling (NMDS) ordinations of four fungal phyla-derived SVs in each sample isolated from control and plant sites. The relative abundances of fungal SVs at the class level derived from phyla Ascomycota (A), Basidiomycota (B), Chytridiomycota (C), and Mucoromycota (D) are shown by bar charts in each sample from control (left) and plant (right) sites. Classes based on the SILVA database are indicated by the colors shown on the bottom of the bar graphs. NA and Incertae_Sedis: unassigned class. NMDS ordinations showing differences in β-diversity, based on the Bray–Curtis dissimilarity in fungal SVs derived from phyla Ascomycota (E), Basidiomycota (F), Chytridiomycota (G), and Mucoromycota (H) in the samples. The samples designated as Feb., Apr., May, Jun. and Aug., which were isolated on February, April, May, June and August in 2021, are shown by colored symbols on the right of figures. Samples derived from control (circles) and plant (triangles) sites are also shown. Clusters of samples are surrounded by dotted lines in the figures.

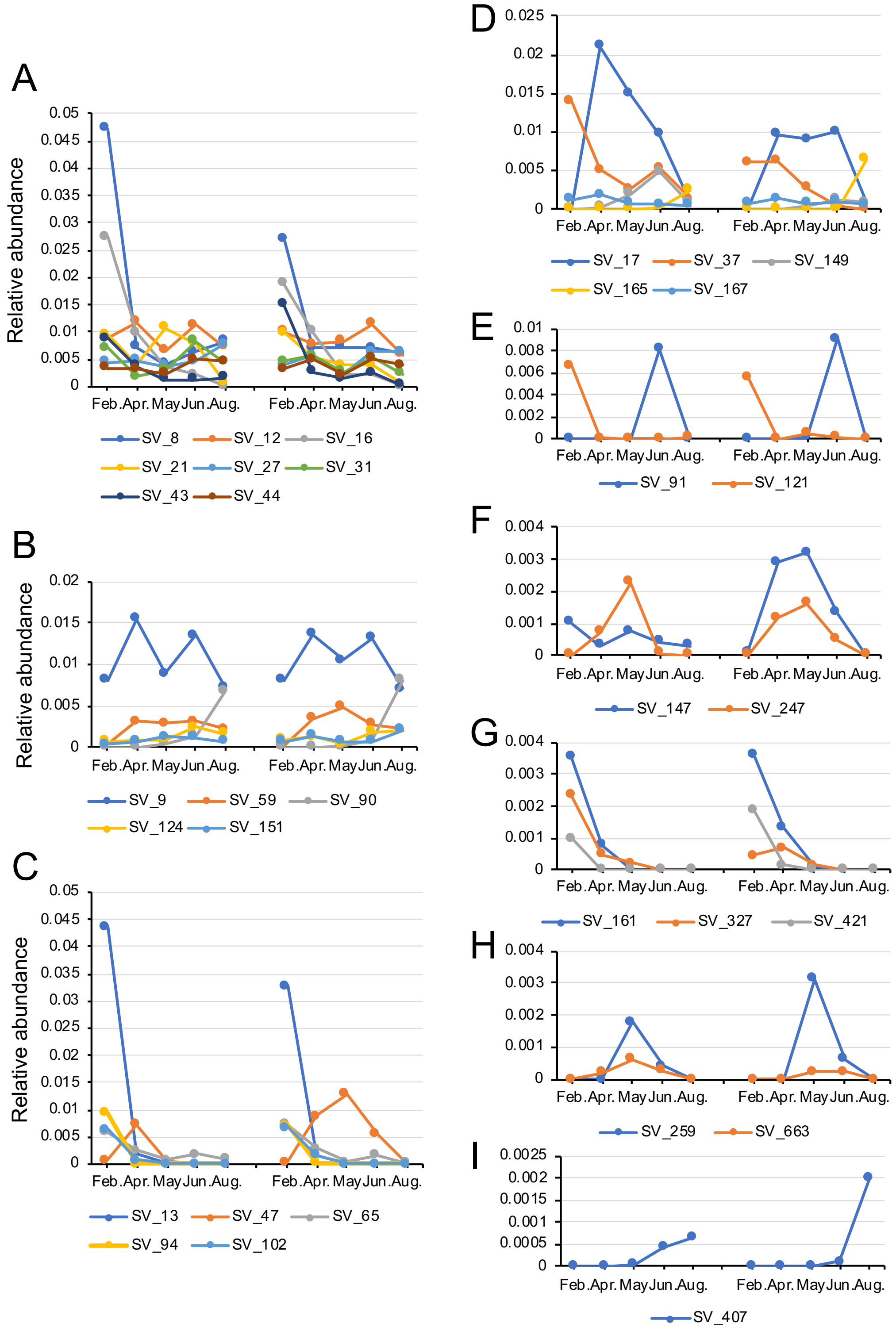

Figure S6. Changes in the relative abundances of abundant SVs in each fungal phylum. The relative abundances of abundant fungal SVs (shown in the bottom of the panels) in total eukaryotic SVs, which are derived from Ascomycota (A), Mucoromycota (B), Chytridiomycota (C), Basidiomycota (D), Aphelidea (E), LKM15 (F), Cryptomycota (G), Blastocladiomycota (H), and Zoopagomycota (I), are indicated in the samples (see Table S2). The left and right line graphs indicate the relative abundances of SVs at control and plant sites, respectively.

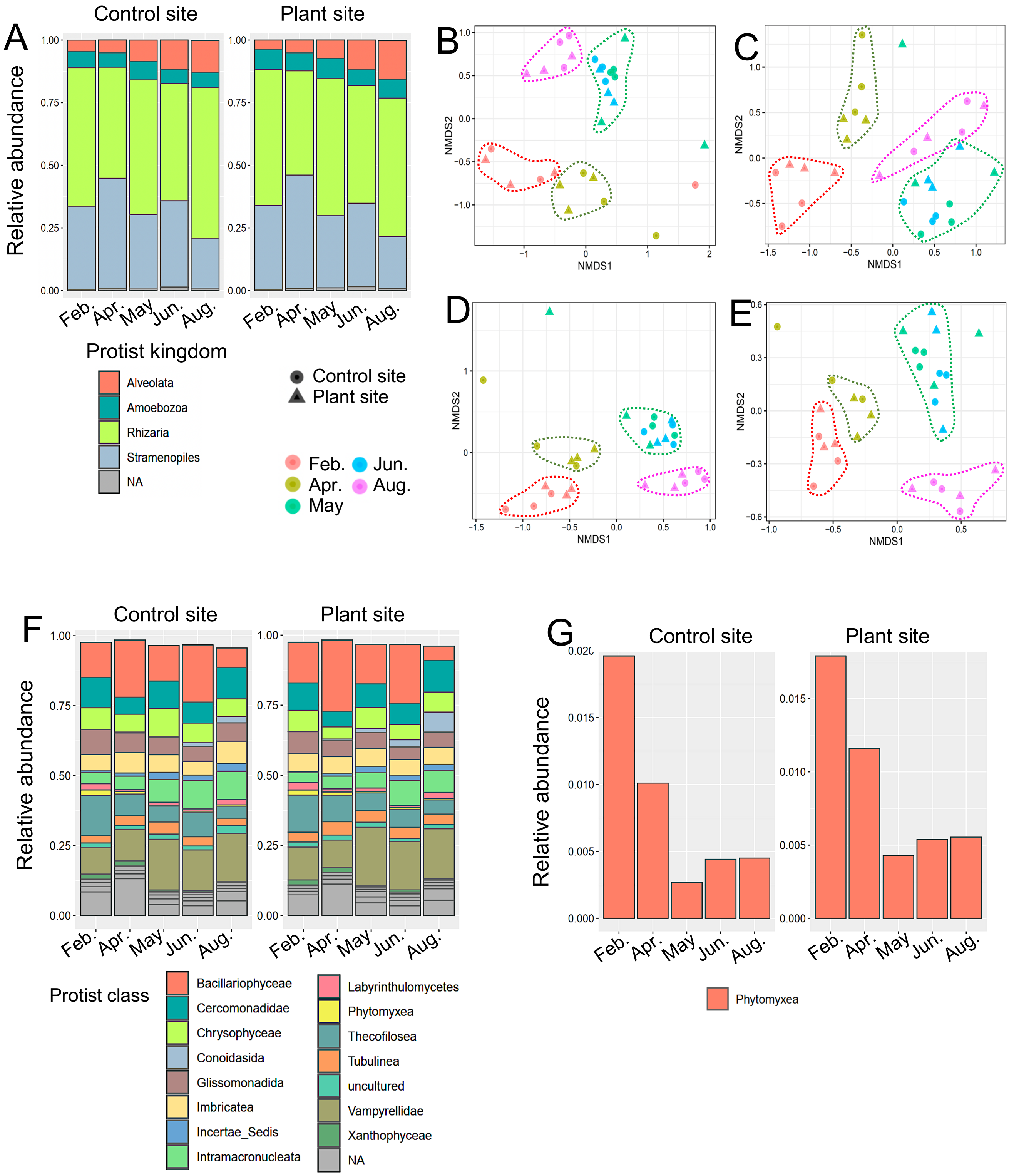

Figure S7. Taxonomic compositions and Bray–Curtis dissimilarity plots of protist-derived SVs in the samples. (A) The relative abundances of kingdoms-derived SVs in total protists-derived SVs are shown in the samples. The taxa based on the SILVA database are indicated by the colors shown on the right of the bar graphs. NA: unassigned protist clades. The Bray–Curtis dissimilarity plots of SVs assigned to kingdoms Alveolata (B), Amoebozoa (C), Rhizaria (D), and Stramenopiles (E) are shown. Each sample

and sampling site are indicated by colors and symbols (circles: control site; triangles: plant site) as shown on the left side of the figures. The compositions of protists class-derived SVs (F) in total protists SVs, and the relative abundances of Phytomyxea-derived SVs in total eukaryotic SVs (G) are indicated by bar charts in the samples. Bar charts of the samples derived from control and plant sites are shown on the left and right, respectively. NA and Incertae_Sedis: unassigned protist classes. uncultured: uncultured protists.

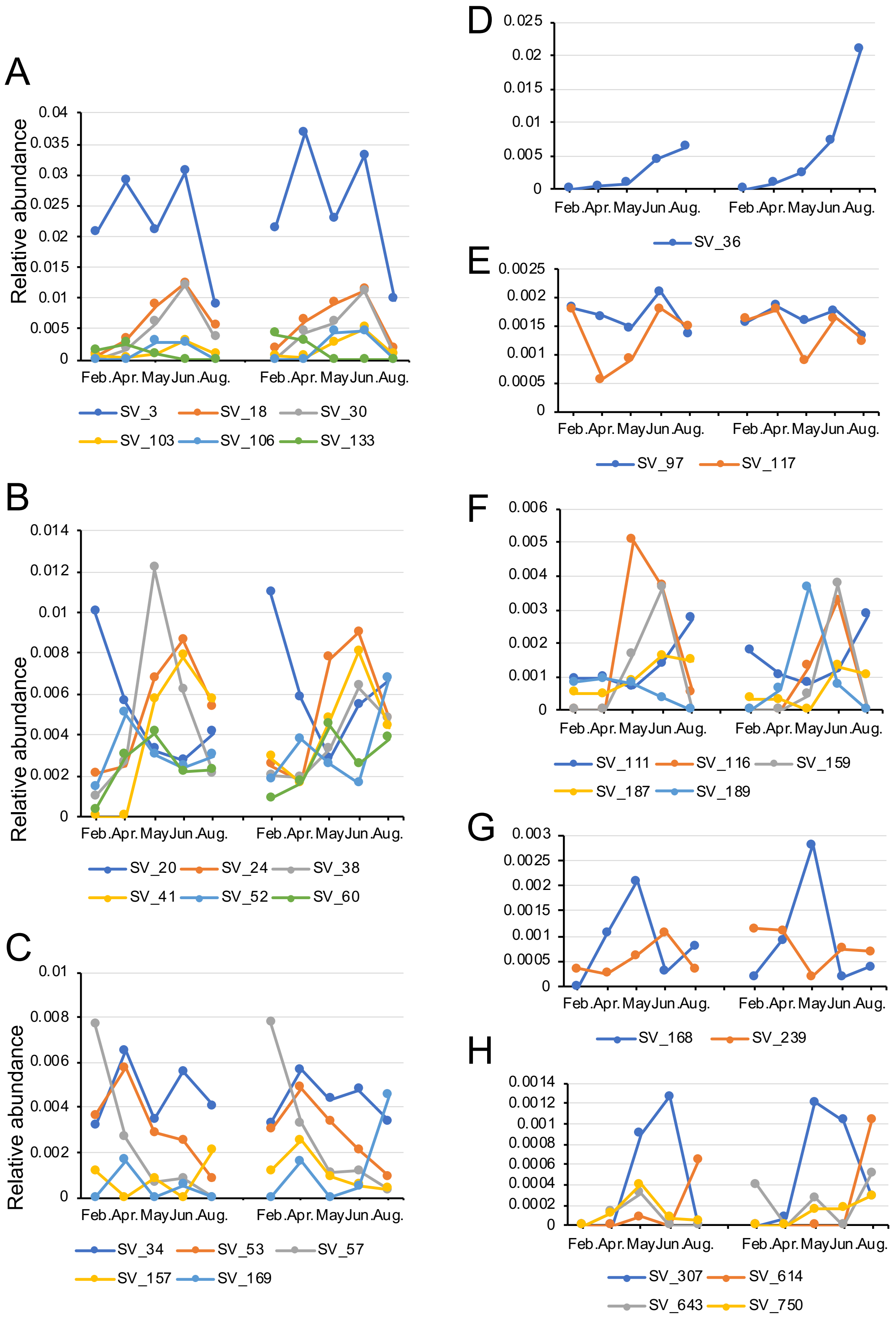

Figure S8. Changes in the relative abundances of abundant SVs in each protistian phylum. The relative abundances of abundant SVs shown in the bottom of the panels of Diatomea (A), Cercozoa (B), Peronosporomycetes (C), Apicomplexa (D), Hyphochytriomycetes (E), Ciliophora (F), Centrohelida (G), and Bicosoecida (H), in total eukaryotic SVs are indicated by line graphs in the samples (see Table S2). The left and right graphs indicate the results from control and plant sites, respectively.

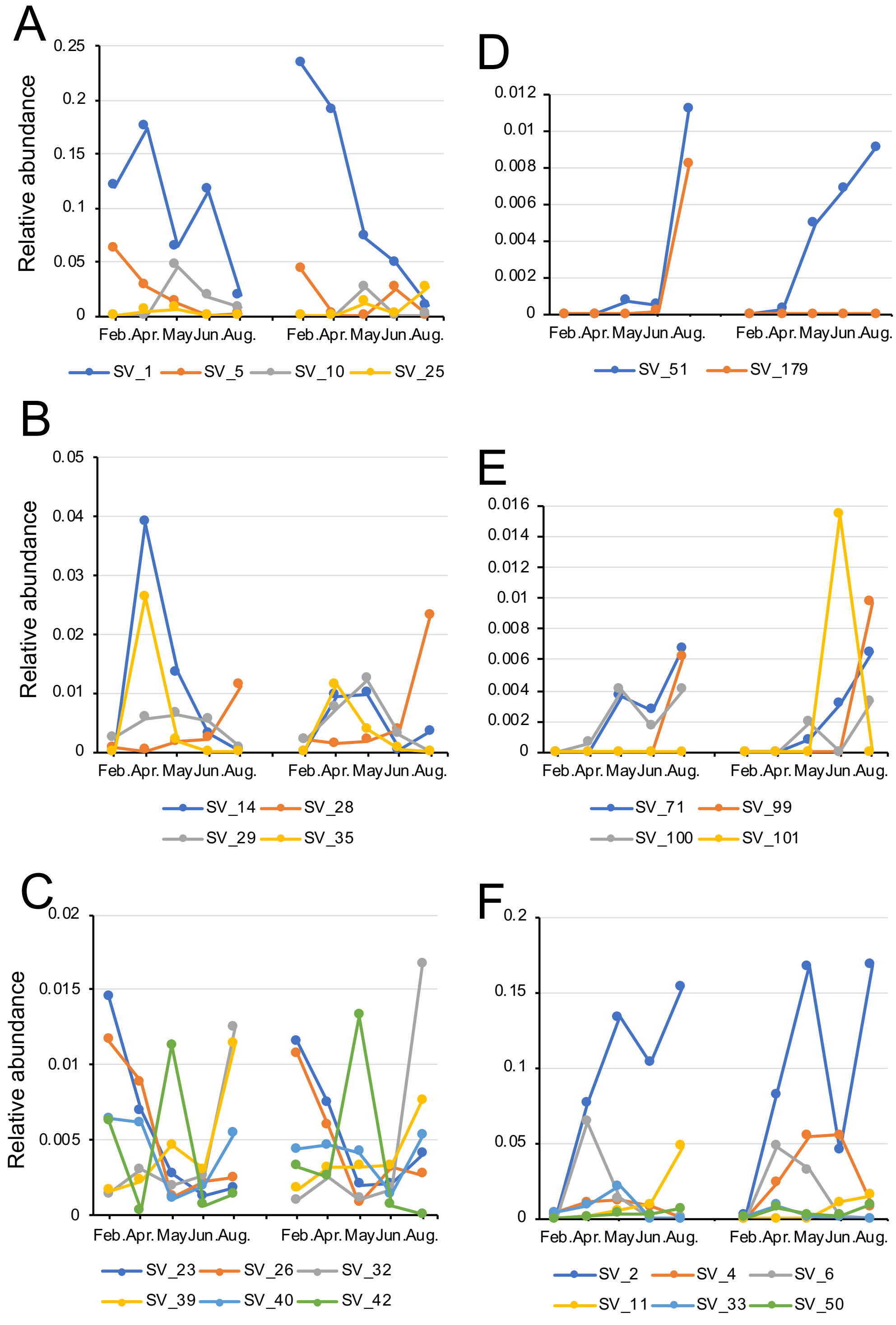

Figure S9. Changes in the relative abundances of animal- and plant-derived abundant SVs in each phylum. The relative abundances of abundant SVs shown on the bottom of the panels of Annelida (A), Arthropoda (B), Nematozoa (C), Gastrotricha (D), Platyhelminthes (E), and Phragmoplastophyta (plants) (F), in total eukaryotic SVs are indicated by line graphs in the samples (see Table S2). The left and right graphs indicate the results from control and plant sites, respectively.

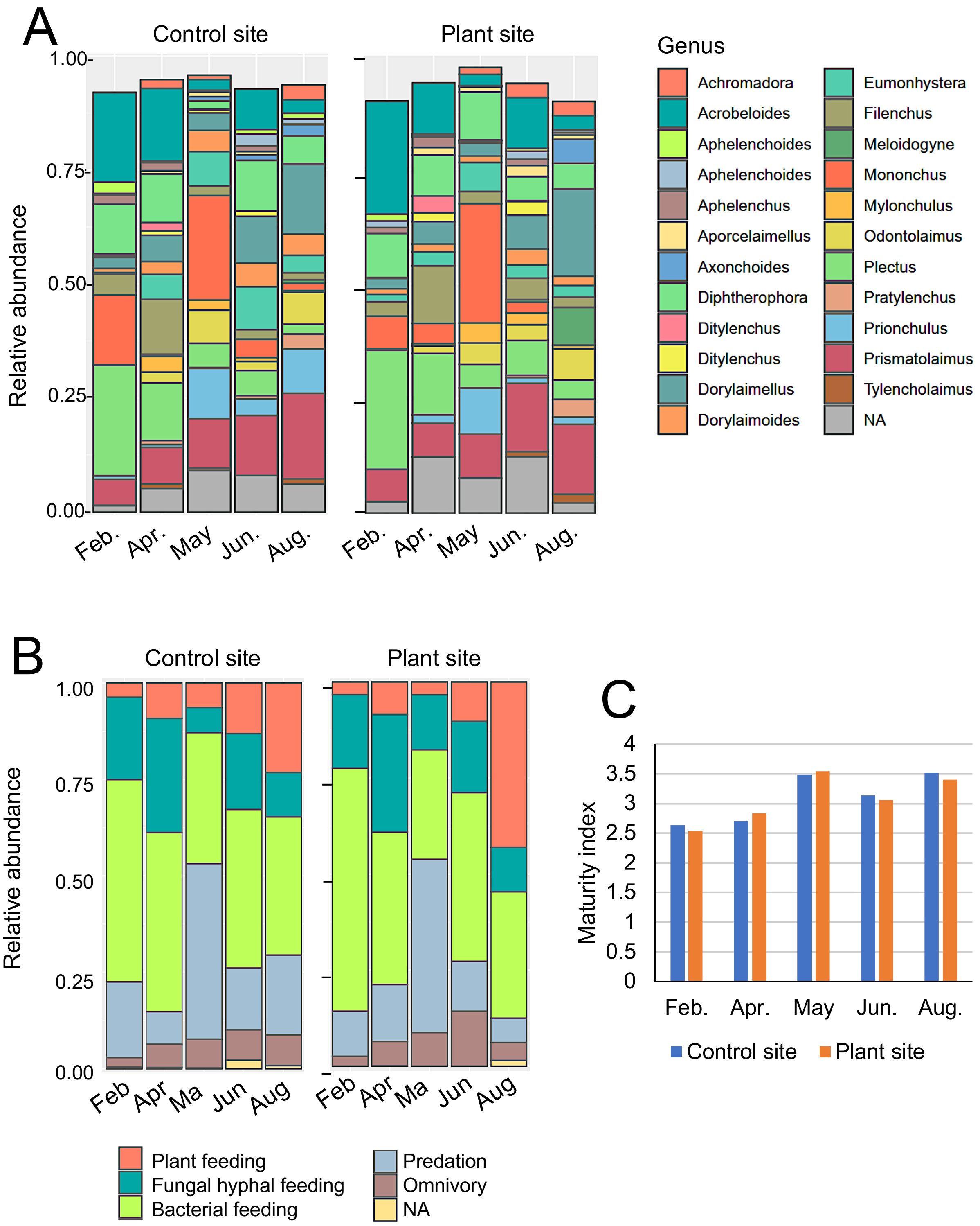

Figure S10. Compositions of nematode genus-derived SVs and feeding habitat-classified SVs in total Nematozoa-derived SVs in the samples. The former and latter are shown in the panel (A) and (B), and the results from the control and plant sites are shown on the left and right, respectively. The SILVA database-based genera are shown on the right of (A). NA: unassigned nematode genus. Feeding types of SVs are derived from Table S3 as shown on the bottom of (B). NA: unassigned feeding habitat. (C) Maturity index of nematode-derived SVs are indicated by bar charts in the samples derived from control and plant sites.

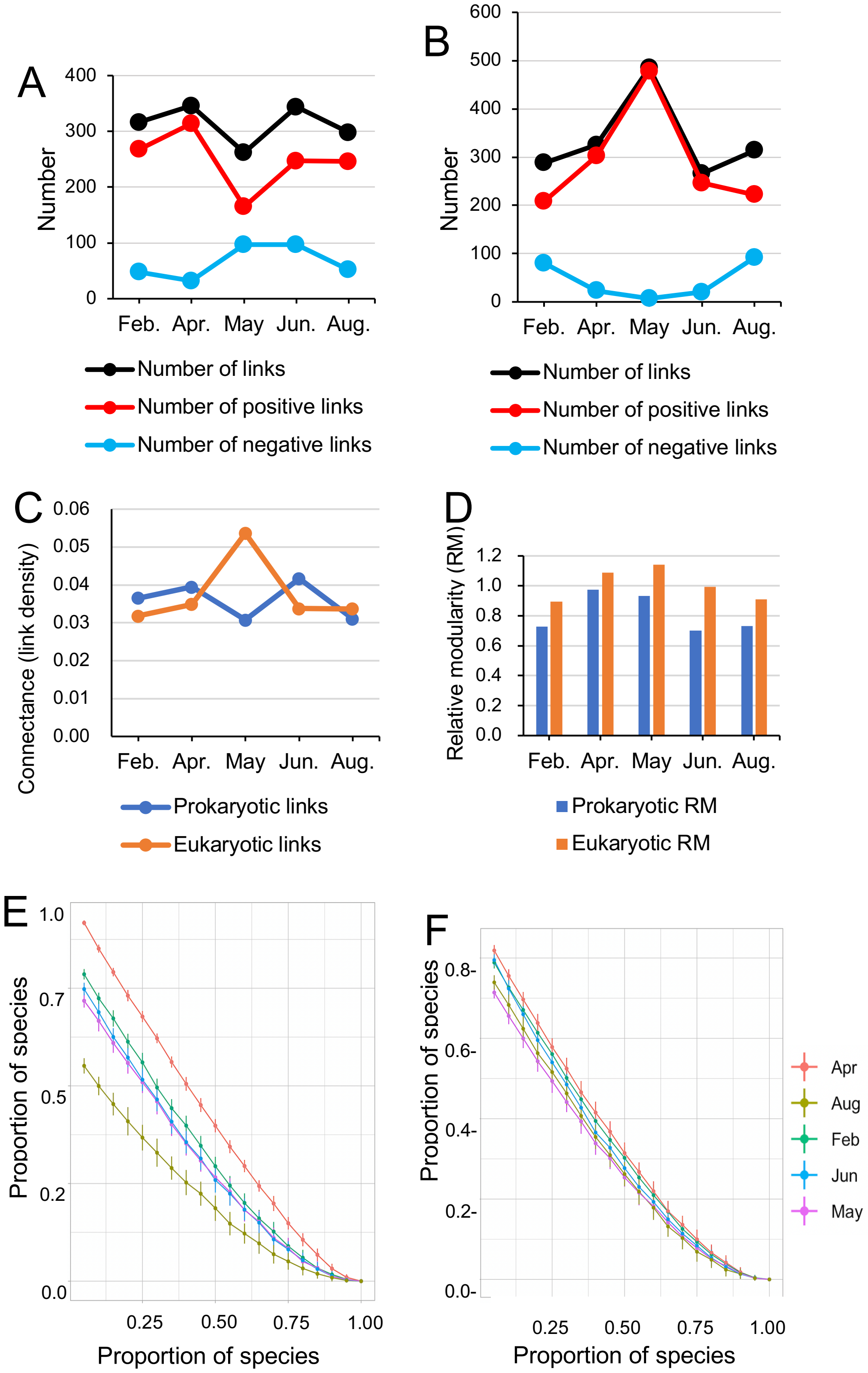

Figure S11. The properties of co-occurrence networks. Numbers of links (black lines), positive (red lines) and negative (blue lines) links in prokaryotic (A) and eukaryotic (B) networks are indicated in each samples. Connectance (link density) and relative modularity of prokaryotic (blue bars) and eukaryotic (orange bars) networks in the samples are shown. Robustness of the networks in the samples were investigated and the proportions of remained in prokaryotic (E) and eukaryotic (F) networks are indicated.
